# Mutational constraints on RSV F and its neutralization by antibodies

**DOI:** 10.1038/s41586-026-11030-4

**Published:** 2026-09-16

**Authors:** Cassandra A. L. Simonich, Teagan E. McMahon, Gavin Juviler, Lucas Kampman, Helen Y. Chu, Jesse D. Bloom

**Affiliations:** 1 Basic Sciences and Computational Biology Divisions, Fred Hutchinson Cancer Center, Seattle, WA USA; 2 Department of Pediatrics, University of Washington, Seattle, WA USA; 3 Pediatric Infectious Diseases Division, Seattle Children’s Hospital, Seattle, WA USA; 4 Division of Allergy and Infectious Diseases, University of Washington School of Medicine, Seattle, WA USA; 5 Howard Hughes Medical Institute, Seattle, WA USA

**Keywords:** Virology, Vaccines, Molecular evolution

## Abstract

New antibodies targeting the F protein of respiratory syncytial virus (RSV) have substantially reduced infant hospitalizations^1^. However, viral resistance is a concern: one antibody failed clinical trials because of a resistant strain^2^, and sporadic resistance mutations to the most widely used antibody (nirsevimab) have been identified^3–6^. Here we define how RSV F mutations affect antibody neutralization. We first provide a biophysical model of how the buffering of bivalent IgG binding combines with the lower Fab potency of nirsevimab to subtype B to make resistance to this antibody more common in subtype B than A strains. We then perform pseudovirus deep mutational scanning to safely measure how nearly all mutations to F affect its cell entry function and neutralization by IgG and Fab forms of nirsevimab, clesrovimab and several other key antibodies. We use these measurements to enable real-time surveillance of RSV sequences for antibody resistance, and show that resistant strains have arisen sporadically but are at present rare. Overall, our work shows how Fab potency and epitope specificity combine to determine how viral mutations affect antibody neutralization, enables monitoring for natural RSV strains resistant to antibodies of public-health importance, and can help guide development of future antibodies with resilience to viral escape.

## Abstract (web summary)

How respiratory syncytial virus mutations affect antibody neutralization is defined, and a biophysical model of the mechanism is provided, to show how Fab potency and epitope specificity combine to determine the effects of viral mutations on antibody neutralization.

## Main

Respiratory syncytial virus (RSV) is a leading cause of infant hospitalization in the USA and infant mortality globally^7,8^. Two monoclonal antibodies, nirsevimab and clesrovimab, were recently licensed and recommended to prevent severe RSV disease in infants^9^. Nirsevimab has demonstrated roughly 75–80% effectiveness against RSV-associated hospitalizations of infants^10^ and (along with maternal vaccination) has reduced infant hospitalizations in countries where it is widely used^1^. More antibodies are under development including for low-income and middle-income countries where RSV is a main cause of infant mortality^11,12^.

Nirsevimab and clesrovimab target the prefusion conformation of the RSV fusion protein (F)^13,14^, binding the apex antigenic region Ø and the lateral-face antigenic region IV, respectively. Although F is less variable than the surface proteins of influenza and SARS-CoV-2, it still evolves sufficiently fast to pose challenges for monoclonal antibodies^15,16^. Regeneron’s anti-F antibody suptavumab failed a phase 3 clinical trial from 2015 to 2017 because it was escaped by mutations in circulating RSV-B strains^2^. Whereas nirsevimab has been in use in the USA for 3 years and retained high effectiveness, sporadic resistant strains have been identified, particularly in breakthrough infections^3–6,16–20^. Therefore, surveillance of RSV sequences for potential escape mutations is an important public-health priority^4,6,20,21^. However, surveillance is hindered by incomplete knowledge of how F mutations affect key antibodies, as previous studies have identified only a limited set of resistance mutations by passaging laboratory-adapted RSV in the presence of antibody^5,14,22^ or looking for mutations in antibody-binding footprints^4–6,17,20^.

Here we provide a complete quantitative understanding of how RSV mutations affect antibodies by combining a biophysical model of bivalent IgG neutralization with deep mutational scanning measurements of how all amino acid mutations affect F’s cell entry function and neutralization by the IgG and Fab forms of key antibodies. Our work explains why nirsevimab resistance mutations are more common in one of the two RSV subtypes, defines the potential for escape from existing and candidate clinical antibodies, and informs a sequence-surveillance platform to rapidly identify antibody-resistant natural RSV strains.

## Bivalent IgG binding buffers resistance

Surveillance of individuals who received prophylaxis and experimental studies performed for resistance analysis for Food and Drug Administration (FDA) licensure have found that viral mutations that escape nirsevimab are more common and have a higher impact in subtype B than subtype A RSV strains^3–6,16–20^. This difference has been puzzling, as nirsevimab has a near-identical binding footprint on the F proteins of subtypes A and B^13^, which have similar structures^23,24^ and roughly 90% protein sequence identity^25^.

**Fig. 1:**
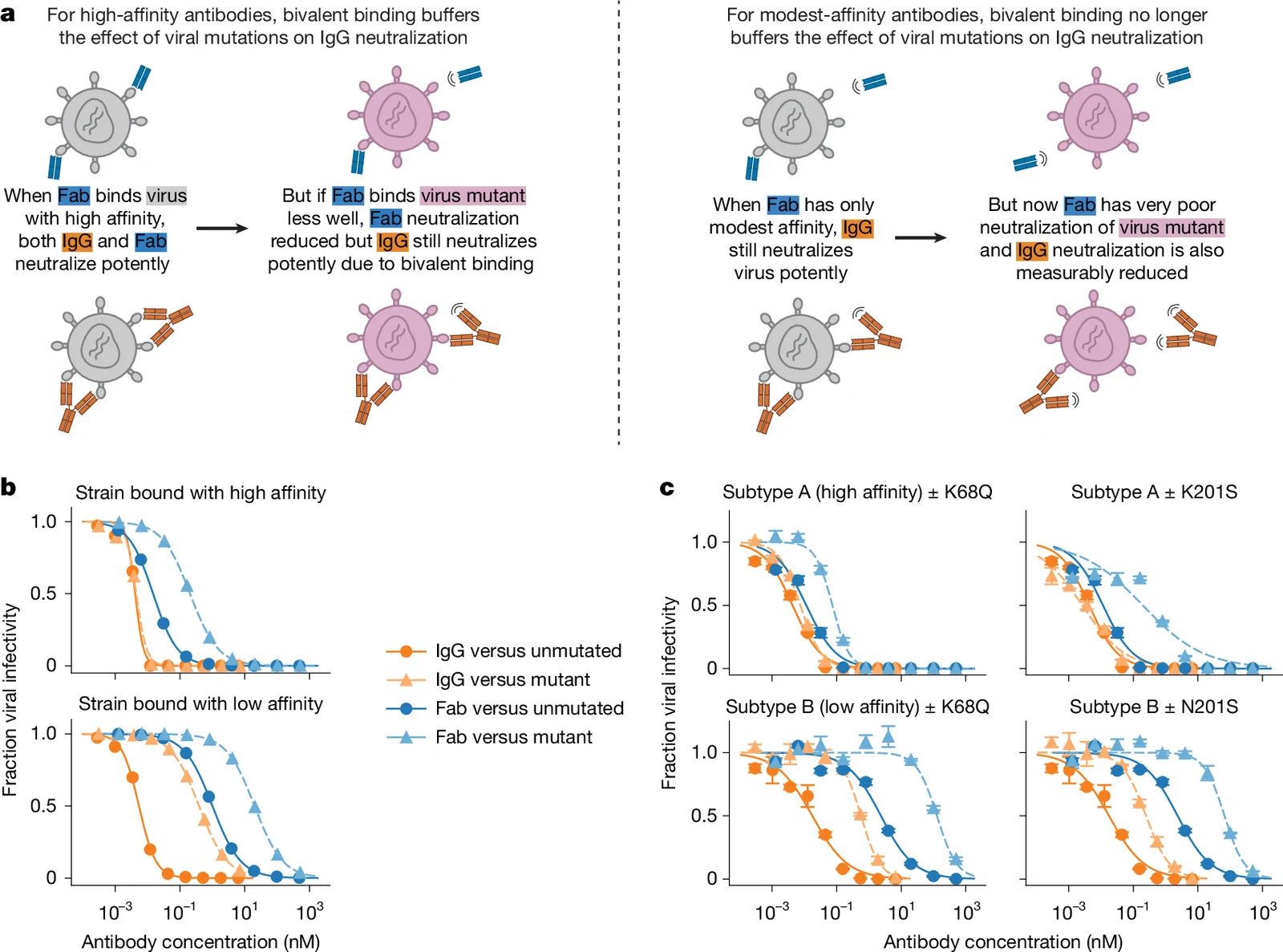
RSV F mutations cause more nirsevimab escape in subtype B versus A because bivalent IgG binding buffering of mutations depends on Fab affinity. **a**, Biophysical model of how bivalent IgG binding buffers mutations that reduce monovalent Fab affinity when the Fab has sufficiently high affinity for the viral antigen, but this buffering vanishes if the Fab affinity is more modest. **b**, Predictions of a quantitative biophysical model (Supplementary Information) of how a viral mutation that reduces Fab affinity affects both IgG and Fab neutralization of a viral strain to which the Fab has high or low affinity. When the Fab has high affinity, mutations that reduce affinity do not appreciably reduce IgG neutralization. **c**, Experimental data showing how two known RSV subtype B nirsevimab escape mutations, K68Q and (K/N)201S, affect neutralization by nirsevimab IgG and Fab of pseudoviruses expressing RSV F from subtype A (Long strain) or subtype B (B1 strain). For all plotted neutralization curves, points represent the mean and s.e.m. of at least two replicate measurements. Nirsevimab has a higher affinity for subtype A than subtype B^19,26^. Consistent with the biophysical model, the mutations reduce Fab neutralization for both subtypes, but only reduce IgG neutralization for subtype B.

We hypothesized that the greater prevalence of escape mutations in RSV-B is because nirsevimab binds with lower affinity to the F from subtype B versus A^19,26^. IgG antibodies, which are produced by the human body and the form in which nirsevimab is administered, have two antigen-binding arms, but antibodies can be artificially produced in a Fab form with just one binding arm. We built a biophysical model of how mutations that reduce the Fab affinity for a viral antigen affect neutralization by both Fab and bivalent IgG (Supplementary Information). The key insight is that when the Fab affinity is sufficiently high, viral mutations that decrease Fab affinity do not measurably decrease IgG neutralization because bivalent binding keeps nearly all the IgG bound to even the mutant virus^27–29^ (Fig. 1a). We used the model to predict how the IgG and Fab forms of an antibody would neutralize a virus to which the Fab had high or moderate affinities, using parameter estimates that roughly reflect nirsevimab against RSV subtypes A and B (Fig. 1b).

We then experimentally measured nirsevimab’s neutralization of pseudovirus expressing F from subtype A or B with two previously described nirsevimab escape mutations K68Q and (K/N)201S (ref. ^20^) (Fig. 1c). The F-protein mutations reduced Fab neutralization of both subtypes but only reduced IgG neutralization for subtype B, consistent with the biophysical model (Fig. 1b,c). These results indicate that nirsevimab escape mutations are more prevalent in subtype B than A because bivalent IgG binding buffers viral mutations in subtype A due to nirsevimab’s higher affinity for that subtype^19,26^. A corollary, which we build on in this paper, is that it can be more generalizable across viral strains to measure how mutations affect Fab neutralization, because the buffering effect of bivalent binding manifests for IgG but not Fab.

## Functional constraint on F

**Extended Data Fig. 1:**
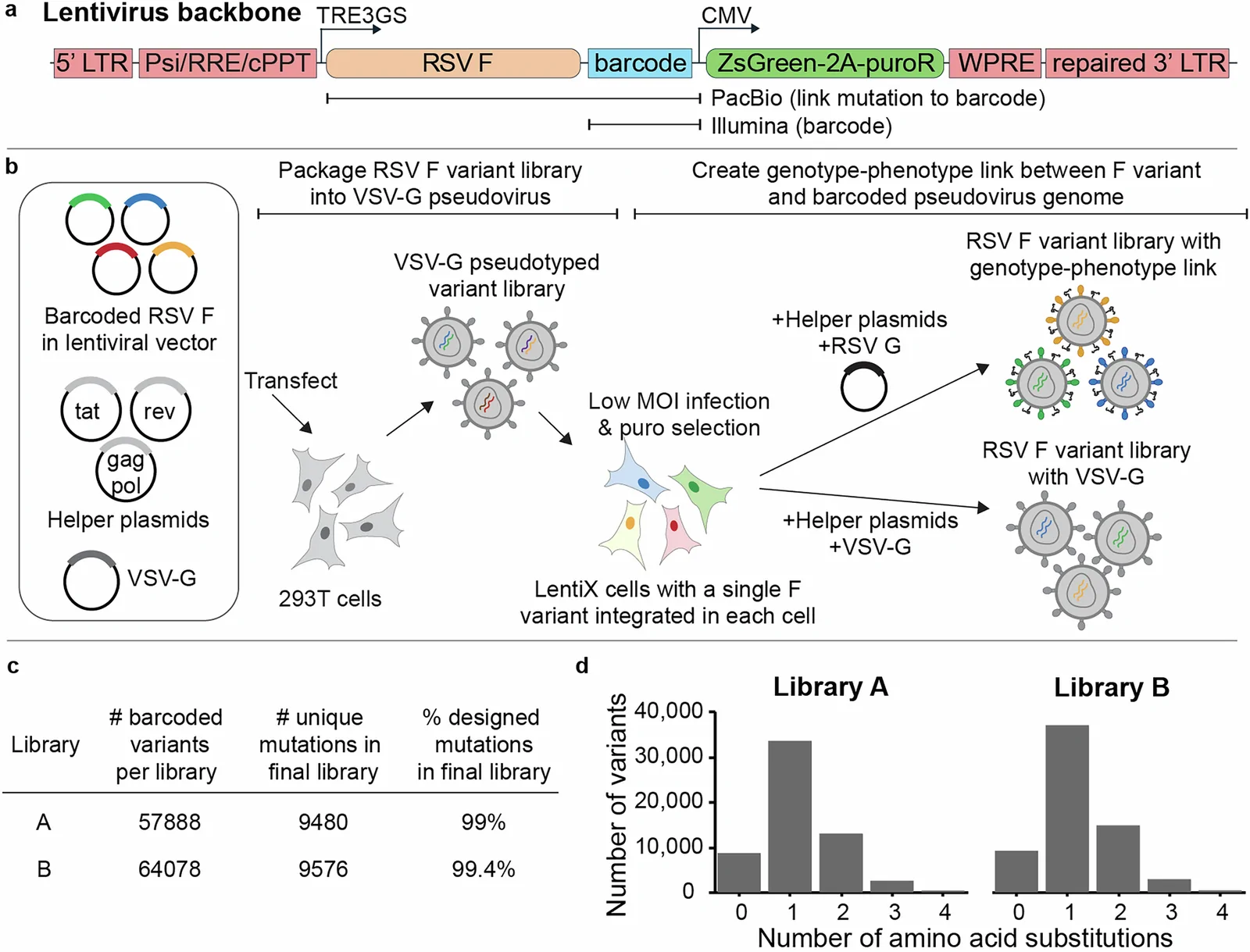
Overview of RSV F deep mutational scanning. (**a**) Lentivirus genome used to make genotype-phenotype linked pseudovirus libraries. Long-read PacBio sequencing is used to link each barcode to a F mutant. Short-read Illumina sequencing is used to quantify the frequency of F variants in experiments. (**b**) Process to generate pseudovirus libraries. We produce VSV-G-pseudotyped lentiviral particles encoding F in their genomes. LentiX cells are transduced by these particles at low MOI (multiplicity of infection, <0.01) so most infected cells receive only one genome integration. Cells with integrated proviruses are selected using puromycin. The library of F-pseudotyped particles is produced by transfecting lentiviral helper plasmids and RSV G. VSV-G pseudovirus is produced as a control to measure library composition independent of F function. PacBio sequencing is performed after integration into cells to account for lentiviral recombination. (**c**) Number of barcoded variants, number of unique F amino acid mutations represented at least once among these variants, and percent of all possible F ectodomain mutations in each of the two pseudovirus libraries. (**d**) Distribution of number of mutations per F variant.

**Extended Data Fig. 2:**
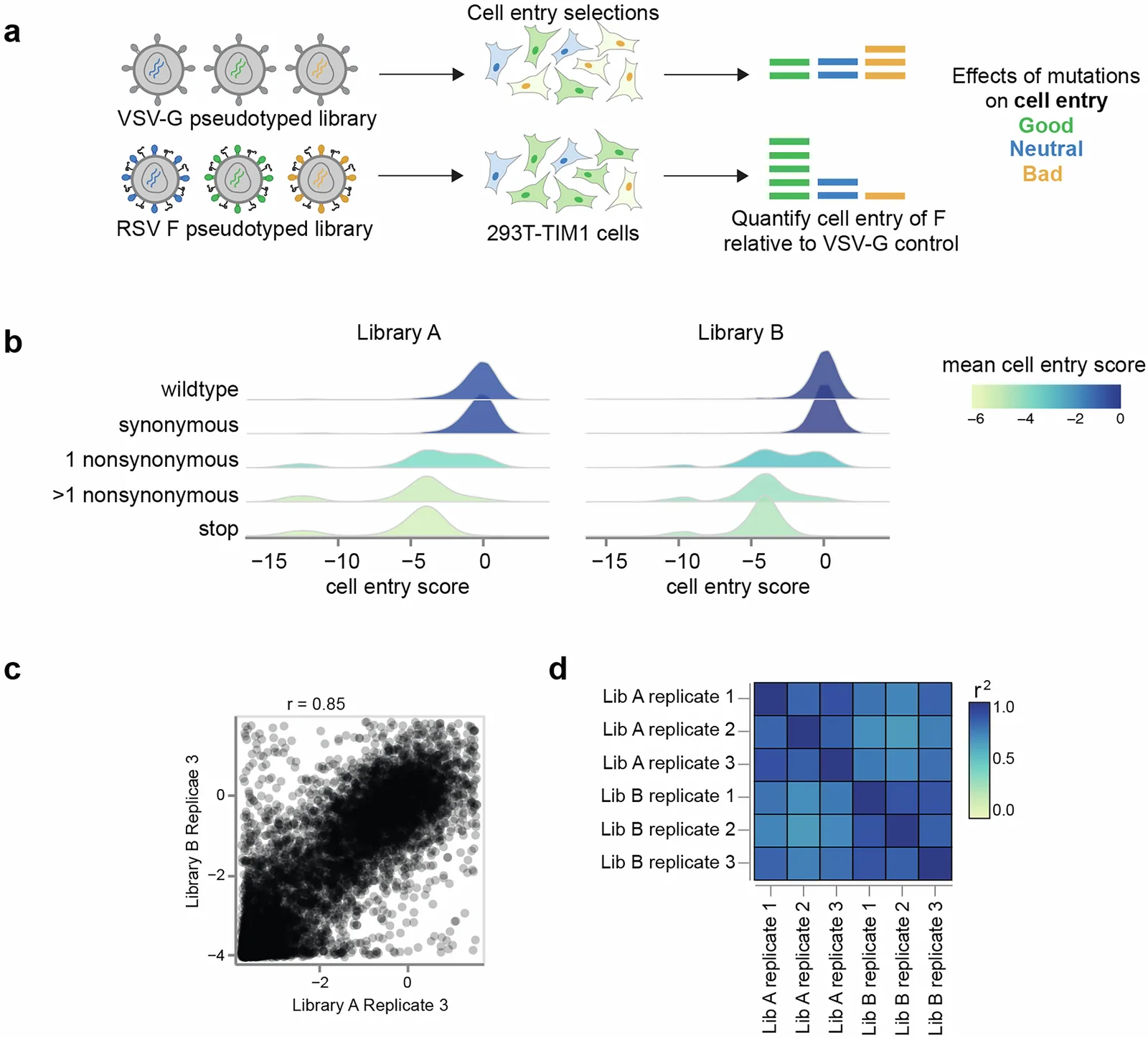
Measurement of how mutations affect F-mediated cell entry. (**a**) Workflow for measuring effects of mutations on cell entry. Pseudovirus libraries expressing RSV F and G or VSV-G are used to infect 293T-TIM1 cells. After infection, lentivirus genomes are isolated from infected cells and barcodes are sequenced to quantify the ability of each F variant to infect cells. Cell entry is quantified as the log2 ratio of the counts of the barcode in the F-mediated entry versus VSV-G control experiments after normalizing these counts to those for the unmutated F protein. So negative values indicate impaired entry and values of zero indicate no effect. (**b**) Distributions of cell entry scores for the two replicate libraries. Histograms are separated by the type of codon mutation in the mutant. (**c**) Pearson correlation (r) of the effect of mutations on cell entry between a single experimental measurement made using each of the two independent libraries. (**d**) Three experimental replicates were done for each of the two libraries. Correlations of the effects of mutations are shown for each pair of replicates.

We used pseudovirus deep mutational scanning^30^ to measure the ability of all amino acid mutants of F to enable pseudotyped lentiviral particles to infect 293T cells expressing the TIM1 attachment factor^31^ (Methods and Extended Data Figs. 1 and 2). These pseudoviruses encode no viral proteins other than F and so can only undergo a single round of cell entry, meaning that they are not fully infectious biological agents capable of causing disease, and so provide a safe way to study mutations to F. We performed the deep mutational scanning using F from the laboratory-adapted subtype A RSV strain termed ‘Long’ after the name of the patient from whom it was isolated in the 1950s (ref. ^32^). We created two independent F-expressing pseudovirus libraries, each of which contained nearly all possible amino acid mutations to F’s ectodomain (Extended Data Fig. 1). Most F mutants contained just a single amino acid mutation, although some contained no or multiple mutations (Extended Data Fig. 1). We quantified the effects of mutations as the log_2_ cell entry of each mutant relative to the unmutated F, using global-epistasis models^33,34^ to jointly analyse the single and multi-mutant data. Negative values indicate a mutation impairs cell entry, whereas positive values indicate it improves cell entry.

**Fig. 2:**
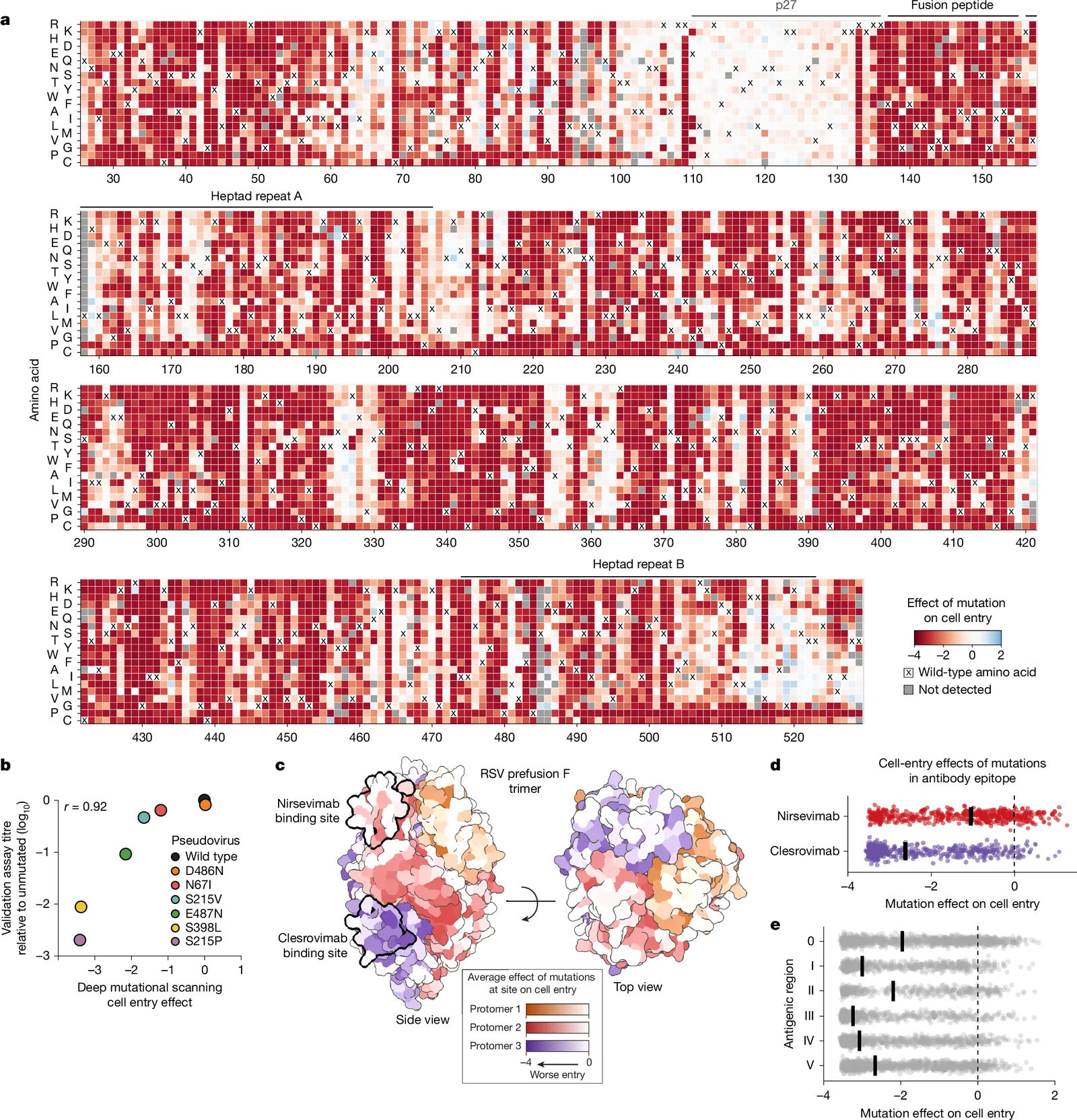
Effects of mutations to RSV F on its cell entry function. **a**, Effects of single amino acid mutations on pseudovirus entry in 293T-TIM1 cells as measured by deep mutational scanning. Each box represents the effect of a specific mutation, with colours indicating decreased (red), unchanged (white) or slightly increased (blue) cell entry relative to the unmutated F. Mutations not reliably measured in our experiments are indicated by grey boxes, and the wild-type amino acid in the parental subtype A Long strain at each site is indicated with an ‘x’. See https://dms-vep.org/RSV_Long_F_DMS/cell_entry.html for an interactive version of this heatmap. **b**, Correlation between mutation effects on cell entry measured by deep mutational scanning and pseudovirus titre in a validation assay using F single-mutation pseudoviruses. **c**, Average effect of mutations at each site mapped onto the prefusion F trimer structure (PDB 5UDC, ref. ^13^), viewed from the top and side. Each protomer is coloured with a separate colour with darker shading indicating more deleterious effects on cell entry. The nirsevimab and clesrovimab binding footprints are outlined on one protomer. **d**, Distribution of mutation effects on cell entry at sites in the epitopes of nirsevimab and clesrovimab (sites that bury 5 Å^2^ or more of surface area on Fab binding). Each point represents an individual mutation (negative values indicate worse cell entry), and the thick black lines indicate the median. **e**, Distribution of mutation effects on cell entry for mutations at sites that comprise broadly defined antigenic regions^36^. Thick black lines indicate the median. In **d** and **e** the numbers of mutations in each category are as follows: nirsevimab, 473; clesrovimab, 431; site 0, 1,274; site I, 752; site II, 451; site III, 780; site IV, 937; and site V, 1,324. The points are the mean effects across the three experimental replicates with each duplicate library.

Measurements of how all mutations affect F’s cell entry function are shown in Fig. 2a and the interactive plots at https://dms-vep.org/RSV_Long_F_DMS/cell_entry.html. Variants with only synonymous mutations had wild-type-like cell entry scores of zero, variants with stop codon mutations had highly negative scores and variants with amino acid mutations had scores ranging from wild-type-like to highly negative (Extended Data Fig. 2b). We performed three experimental replicates with each of the two independent libraries, and the measured cell entry effects were highly correlated across replicates and libraries (Extended Data Fig. 2c,d); we report the average across all replicates for both libraries. We validated that the deep mutational scanning measurements correlated with the infectious titre of pseudoviruses expressing individual F mutations spanning a range of effects (*r* = 0.92; Fig. 2b).

Many regions of F including the fusion peptide are highly constrained with most mutations deleterious for cell entry (Fig. 2a). The p27 fragment is cleaved from the mature protein to yield the active form of F; the basic residues comprising the cleavage motifs at each end of p27 are intolerant to mutations whereas the cleaved p27 fragment itself is tolerant to mutations (Fig. 2a). The epitope targeted by nirsevimab at the apex of the trimer is more mutationally tolerant than the epitope targeted by clesrovimab on the lateral face of the trimer (Fig. 2c,d). Of the broadly defined antigenic regions^35,36^, region 0 is the most mutationally tolerant, whereas regions III and IV are more constrained (Fig. 2e).

**Extended Data Fig. 3:**
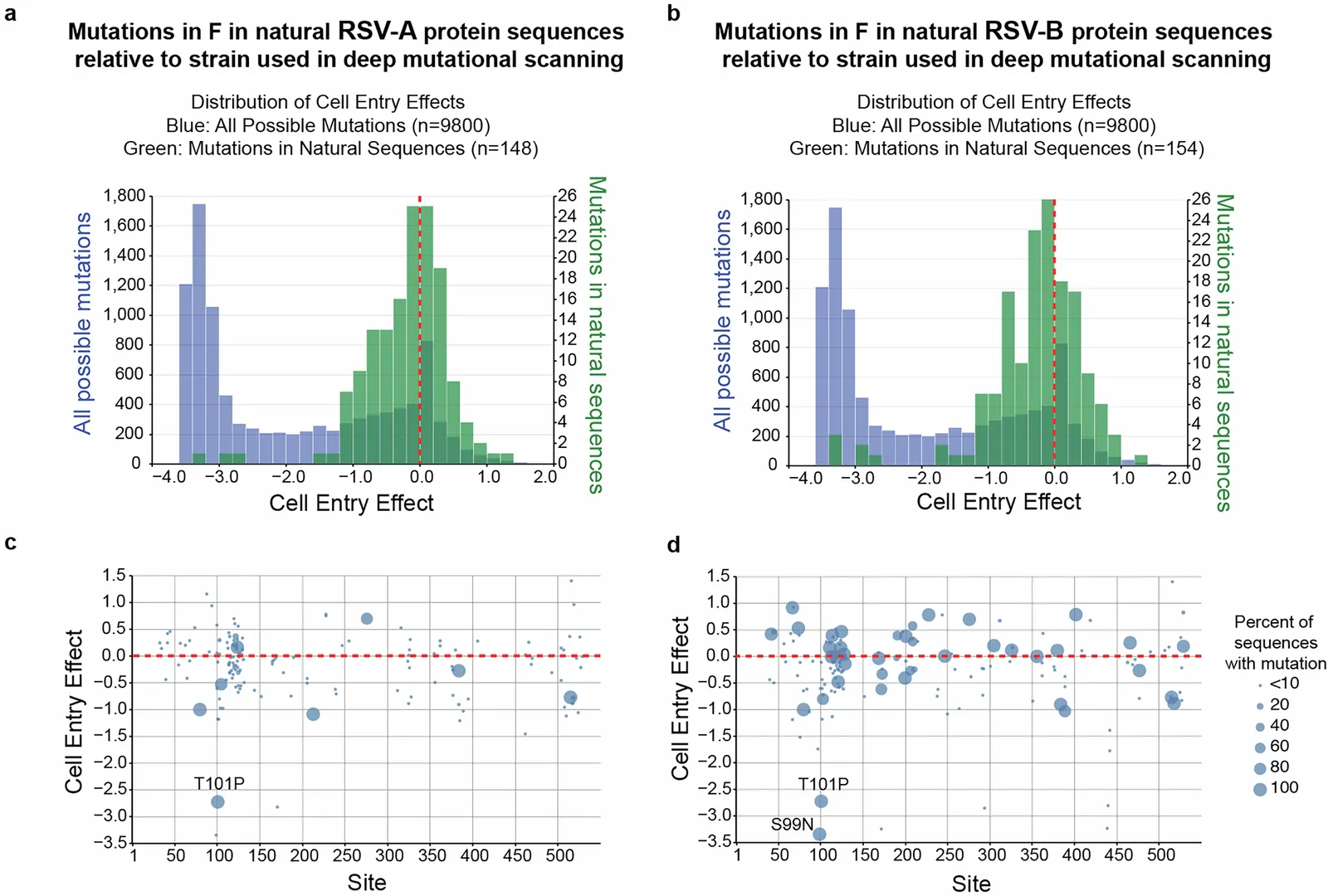
Amino acid differences in natural RSV sequences relative to the strain used for deep mutational scanning mostly have minimal impact on cell entry. (**a**,**b**) Distribution of effects on cell entry (as measured by deep mutational scanning) for all possible F protein mutations (blue) and mutations observed in natural RSV F sequences (green) for subtypes A and B. Mutations are defined relative to the lab-adapted subtype A Long strain used in the deep mutational scanning. The red vertical dashed line indicates an effect of zero, corresponding to no impact on cell entry. (**c**,**d**) Cell entry effects for mutations in natural subtype A and B F sequences relative to the strain used in deep mutational scanning. Each circle represents a mutation, with its x-axis location indicating the site in the protein and its size indicating the percent of F sequences with that mutation. The red dashed line indicates zero effect (neutral). Only two mutations measured to be deleterious to cell entry are at high frequency in natural sequences, T101P and S99N. T101P is found in nearly all natural sequences and S99N is found in nearly all natural subtype B sequences. S99N is likely deleterious in the lab-adapted strain as it creates an N-linked glycosylation site near the F cleavage site. However, this glycosylation site is not formed in natural sequences due to no S/T residues at site 101. T101P is a determinant of human metapneumovirus growth in cell culture^72^, suggesting the difference between the lab-adapted strain and natural sequences may be a lab-adaptation.

Most F mutations observed in natural RSV sequences relative to the laboratory-adapted parental strain used in our deep mutational scanning have minimal impact on cell entry (Extended Data Fig. 3), as expected because there is strong natural selection for F to retain this function. The main exception is that cell entry is impaired by two mutations (S99N and T101P) that revert sites near an F proteolytic cleavage motif from their identities in the laboratory-adapted strain to amino acids commonly observed in natural sequences (Extended Data Fig. 3). This exception highlights the fact that there can be discrepancies between how mutations affect pseudovirus entry in cell culture versus real-world viral fitness: presumably the N99 and P101 identities in natural sequences are better for authentic viral infection of human airway cells even if S99 and T101 give better pseudovirus entry into a cell line.

## Nirsevimab neutralization of F mutants

**Fig. 3:**
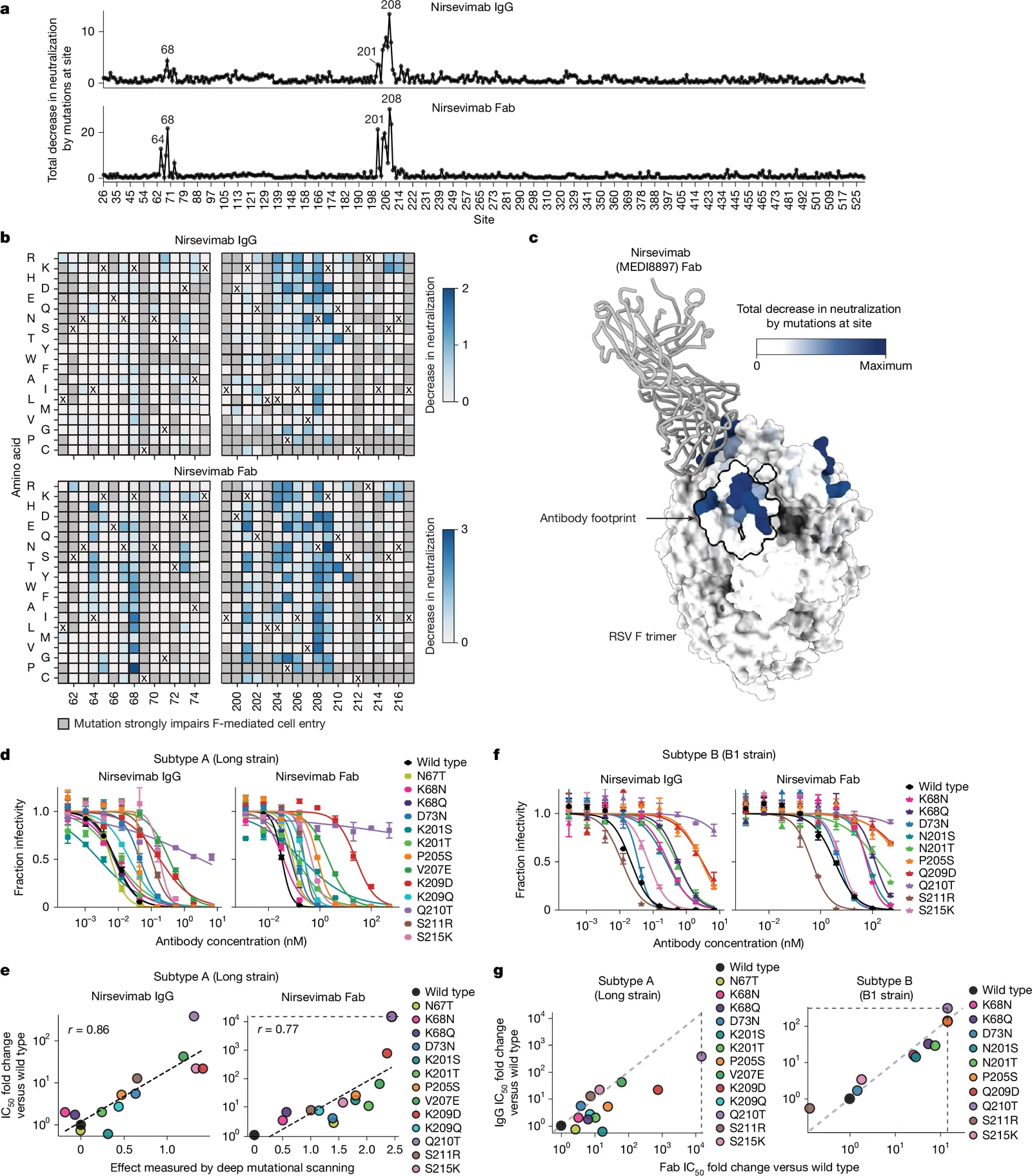
Effects of mutations to RSV F on neutralization by nirsevimab IgG and Fab. **a**, Total decrease in neutralization from all mutations at each site for nirsevimab IgG or Fab. See https://dms-vep.org/RSV_Long_F_DMS/nirsevimab_neutralization.html for interactive plots that show all mutations. **b**, Heatmaps showing effects of mutations within the epitope of nirsevimab. Each box represents a single mutation, with positive values indicating decreased neutralization. Dark grey indicates mutations that are too deleterious for cell entry to measure their effect on neutralization. The wild-type amino acid in the subtype A Long strain used for the deep mutational scanning is indicated with an ‘x’. **c**, Prefusion RSV F trimer (PDB 5UDC, ref. ^13^) in complex with nirsevimab Fab (grey cartoon) coloured by the total effect of mutations at each site on neutralization by nirsevimab Fab. **d**, Neutralization curves for nirsevimab IgG or Fab versus pseudovirus expressing the indicated Long strain F mutants. **e**, Correlation of mutation effects on neutralization by nirsevimab in the neutralization assays versus deep mutational scanning for IgG or Fab for mutants of the subtype A Long strain F. Horizontal dashed line indicates limit of detection. **f**, Neutralization curves for nirsevimab IgG or Fab versus pseudovirus expressing the indicated mutants of F from the subtype B strain B1. **g**, Comparison of fold change in IC_50_ between nirsevimab IgG and Fab for mutations in subtype A background (Long strain) and subtype B background (B1 strain). In **d** and **f**, curves for K68Q and K/N201T are duplicated from Fig. 1.

**Extended Data Fig. 4:**
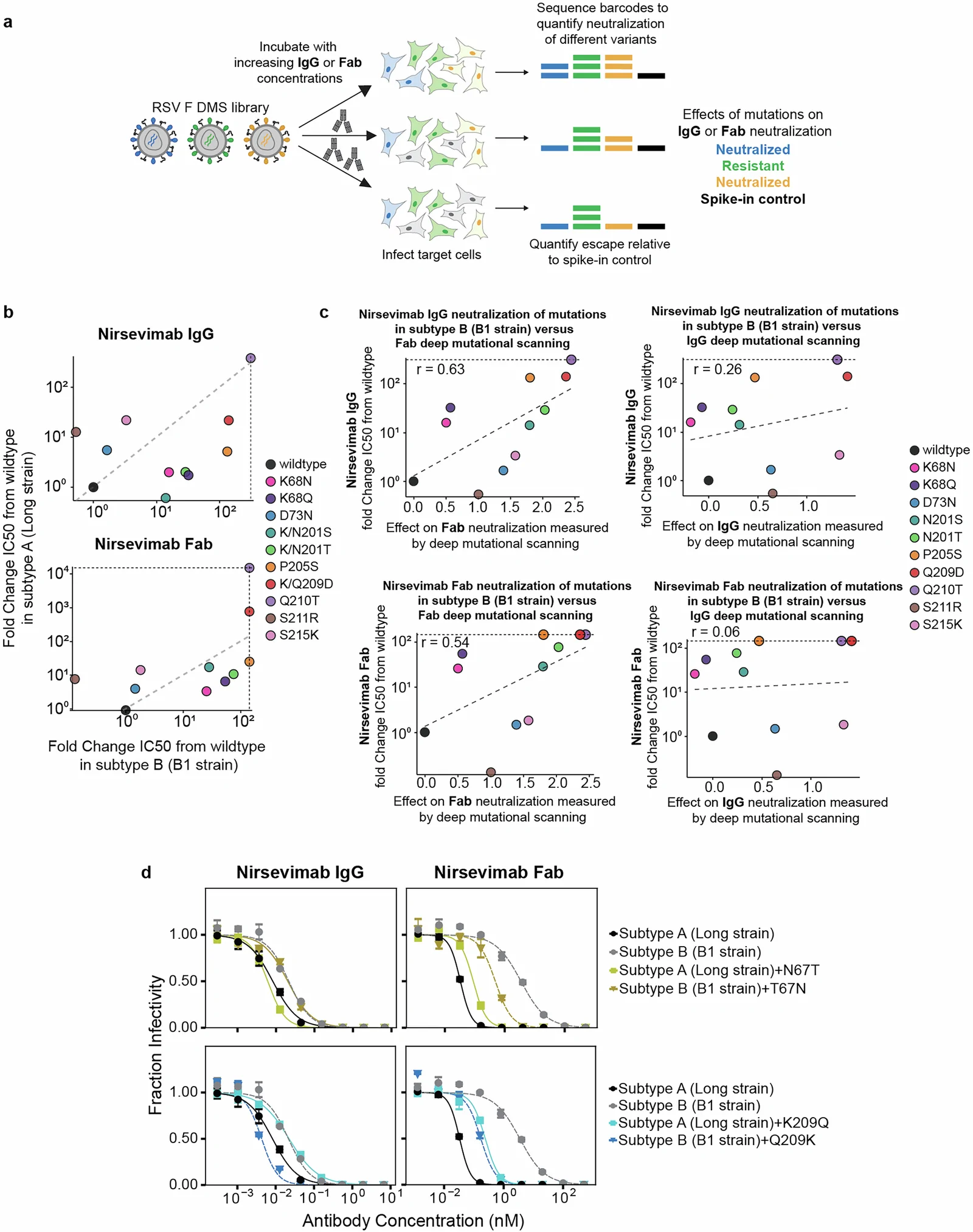
Deep mutational scanning measurements versus effects of mutations on subtype B as measured in validation neutralization assays. (**a**) Workflow for measuring effects of mutations on antibody neutralization using deep mutational scanning. Pseudovirus libraries expressing RSV F and G are used to infect 293T-TIM1 cells after incubation with increasing concentrations of antibody (IgG or Fab). After infection, lentivirus genomes are isolated from infected cells and combined with equal amounts of a DNA spike-in standard containing known barcodes. The barcodes are sequenced to quantify the ability of each F variant to infect cells in each antibody concentration, with barcode sequencing counts converted to fraction infectivity by normalizing them to the counts for the spike-in standard (see Methods). Details including all data for experimental replicates are shown here https://github.com/dms-vep/RSV_Long_F_DMS. (**b**) Correlation between effects of mutations on neutralization IC50 of subtype A (Long strain) or subtype B (B1 strain) F pseudoviruses in validation neutralization assays with nirsevimab IgG and Fab. Dashed gray line shows 1:1. (**c**) Correlation between effects of mutations in the deep mutational scanning of subtype A F with nirsevimab Fab or IgG versus effects on IC50 in validation neutralization assays using a subtype B (B1 strain) F. As predicted by the biophysical model, deep mutational scanning with the Fab is more predictive of effects of mutations in subtype B. Horizontal and vertical dashed lines indicate limits of detection. (**d**) Differences between subtype A and B at F sites 67 and 209 contribute to reduced potency of nirsevimab against subtype B. These neutralization curves are duplicated from Fig. 3, and the same curves for the unmutated subtype A (Long strain) and subtype B (B1 strain) are plotted in both the top and bottom rows.

We next measured how all functionally tolerated F mutations affect neutralization by nirsevimab, which was the first antibody widely approved for RSV prophylaxis for infants^37^. We incubated the pseudovirus libraries with a range of antibody concentrations and quantified the ability of each variant to infect cells, converting sequencing counts to fraction pseudovirus infectivity at each concentration using a spike-in standard^30^ (Extended Data Fig. 4a). We measured the effects of mutations on neutralization by both IgG and Fab forms of nirsevimab, as the biophysical model described above suggests that Fab measurements are more generalizable across genetic backgrounds due to the absence of affinity-dependent buffering of bivalent IgG neutralization. We made two replicate measurements for each of the two independent pseudovirus libraries; throughout we report the average across replicates. The mutations that affect neutralization by nirsevimab are at a subset of sites from 64 to 73 and 201 to 216 (Fig. 3a,b and interactive plots at https://dms-vep.org/RSV_Long_F_DMS/nirsevimab_neutralization.html). These sites are all in or near the structurally determined nirsevimab binding site (Fig. 3c). Some mutations at these sites (for example, 64T, 65E, 68Q/N/E, 201S/T, 203I, 208S/D/Y) have been previously shown to affect nirsevimab neutralization^3,5,16–20,22^ or fusion inhibition (for example, 64M/65R, 64V/65E, 68I, 204S, 205S, 208I/K/Y)^4,6^. However, our deep mutational scanning identified numerous mutations that reduce neutralization that have not previously been reported, including mutations to extra amino acid identities at sites 64, 65, 68, 201, 204, 205, 208 and 209 and mutations at sites not previously reported to affect nirsevimab neutralization, including 67, 73, 206–207, 210–211 and 215–216 (Fig. 3b).

To validate the deep mutational scanning, we generated pseudoviruses carrying key mutations in F from the subtype A Long strain and measured their impact in neutralization assays with nirsevimab IgG and Fab (Fig. 3d). The effects of mutations on neutralization half-maximum inhibitory concentration (IC_50_) were highly correlated with the deep mutational scanning (Fig. 3e). Note that these assays validate some of the resistance mutations newly identified by our deep mutational scanning (for example, 73N, 207E, 209D/Q, 210T, 211R, 215K).

To test whether our deep mutational scanning of how mutations affect nirsevimab Fab neutralization of F from a subtype A strain could be extrapolated to a subtype B strain, we generated pseudoviruses carrying key mutations in the F of a subtype B strain (B1, a laboratory-adapted strain from the 1980s, ref. ^38^). Most mutations that reduced Fab neutralization of the subtype A strain also reduced both Fab and IgG neutralization of the subtype B strain (Fig. 3f and Extended Data Fig. 4b,c). Consistent with the biophysical model, deep mutational scanning measurements of how subtype A mutations affect Fab neutralization correlate more strongly with both IgG and Fab neutralization of subtype B mutants than do deep mutational scanning measurements performed with IgG (Extended Data Fig. 4c). The biophysical model also predicts that F mutations will cause a greater reduction in Fab than IgG neutralization for subtype A, but affect IgG and Fab neutralization similarly for subtype B as there is bivalent IgG buffering for the higher-affinity subtype A binding but not the lower-affinity subtype B binding; our measurements validate this prediction (Fig. 3g). Note that S211R slightly increases subtype B neutralization despite decreasing subtype A neutralization for both Fab and IgG; for this mutation there must be more background-specific differences between subtypes.

Our deep mutational scanning also identifies the F sequence differences responsible for the lower nirsevimab Fab potency to subtype B than subtype A. Most subtype A strains have N at site 67 and K at 209, whereas most subtype B strains have T at 67 and Q or R at 209. In the deep mutational scanning, N67T and K209Q reduce neutralization by nirsevimab Fab. We confirmed that mutating the subtype A F to the subtype B identities at these sites (N67T and K209Q) reduces neutralization by nirsevimab Fab, whereas the reverse mutations in subtype B (T67N and Q209K) increase neutralization (Extended Data Fig. 4d). The effects of these swap mutations are much larger on Fab versus IgG neutralization, consistent with the biophysical model that posits that the nirsevimab affinity for F is sufficiently high that bivalent binding largely buffers mutation effects on IgG neutralization (Extended Data Fig. 4d).

**Extended Data Fig. 5:**
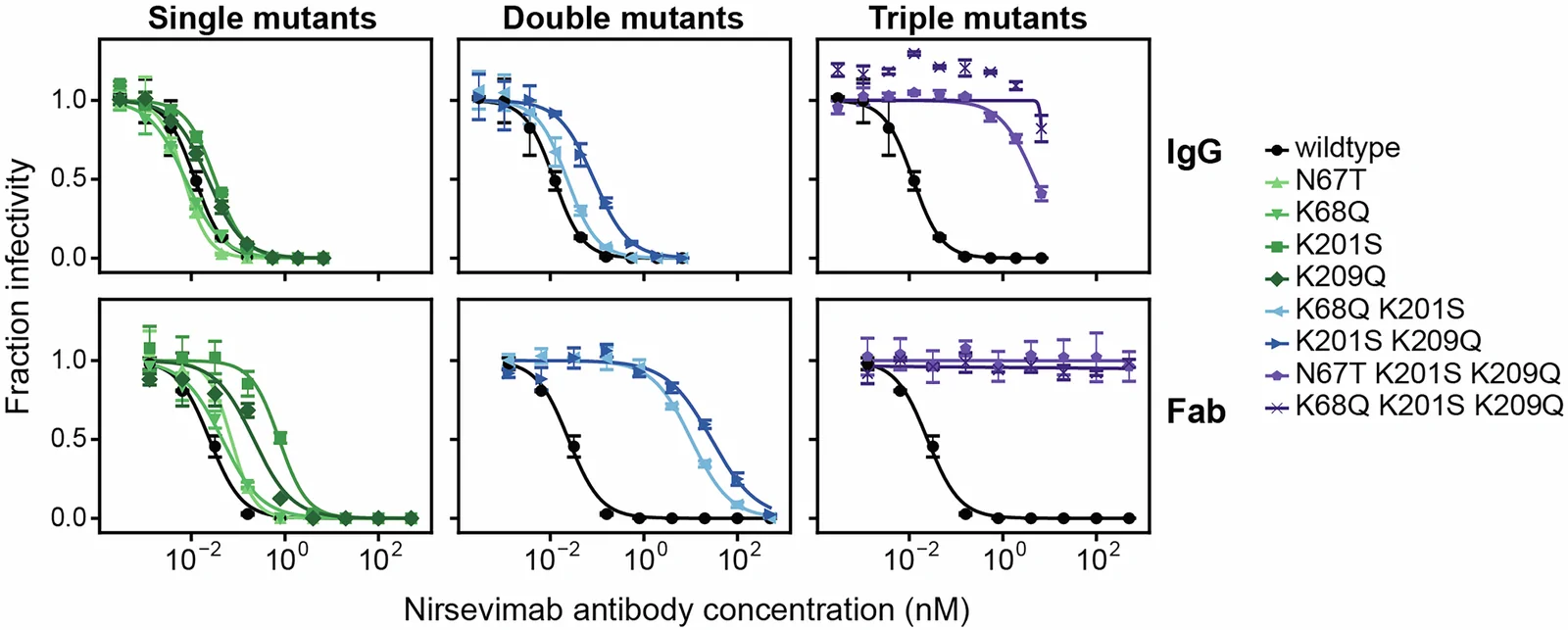
Mutations that individually reduce nirsevimab Fab but not IgG neutralization combine to also reduce IgG neutralization. Neutralization by nirsevimab IgG (top row) or Fab (bottom row) against pseudoviruses expressing RSV F from the subtype A Long strain with the indicated single, double, or triple mutations. Note that the same neutralization curve for the wildtype F from the Long strain against IgG or Fab is re-plotted in all three columns in each row to enable visual comparison of the mutants against wildtype.

Measuring mutation effects on nirsevimab Fab neutralization identifies F mutations to subtype A that affect IgG neutralization in combination even when they individually have little effect. Specifically, the F mutations N67T, K68Q, K201S and K209Q individually reduce nirsevimab Fab but not IgG neutralization of subtype A, but reduce IgG neutralization when combined as double or triple mutants (Extended Data Fig. 5). These results show the relevance of characterizing mutational impacts on Fab neutralization for understanding IgG neutralization of multiply mutated F variants.

## Clesrovimab neutralization of F mutants

Clesrovimab, which targets a different region of F from nirsevimab, was recently approved as another option for RSV prevention for infants^39,40^. We measured the effects of F mutations on neutralization by clesrovimab IgG and Fab, performing two replicate deep mutational scanning experiments for each of the two independent pseudovirus libraries.

**Fig. 4:**
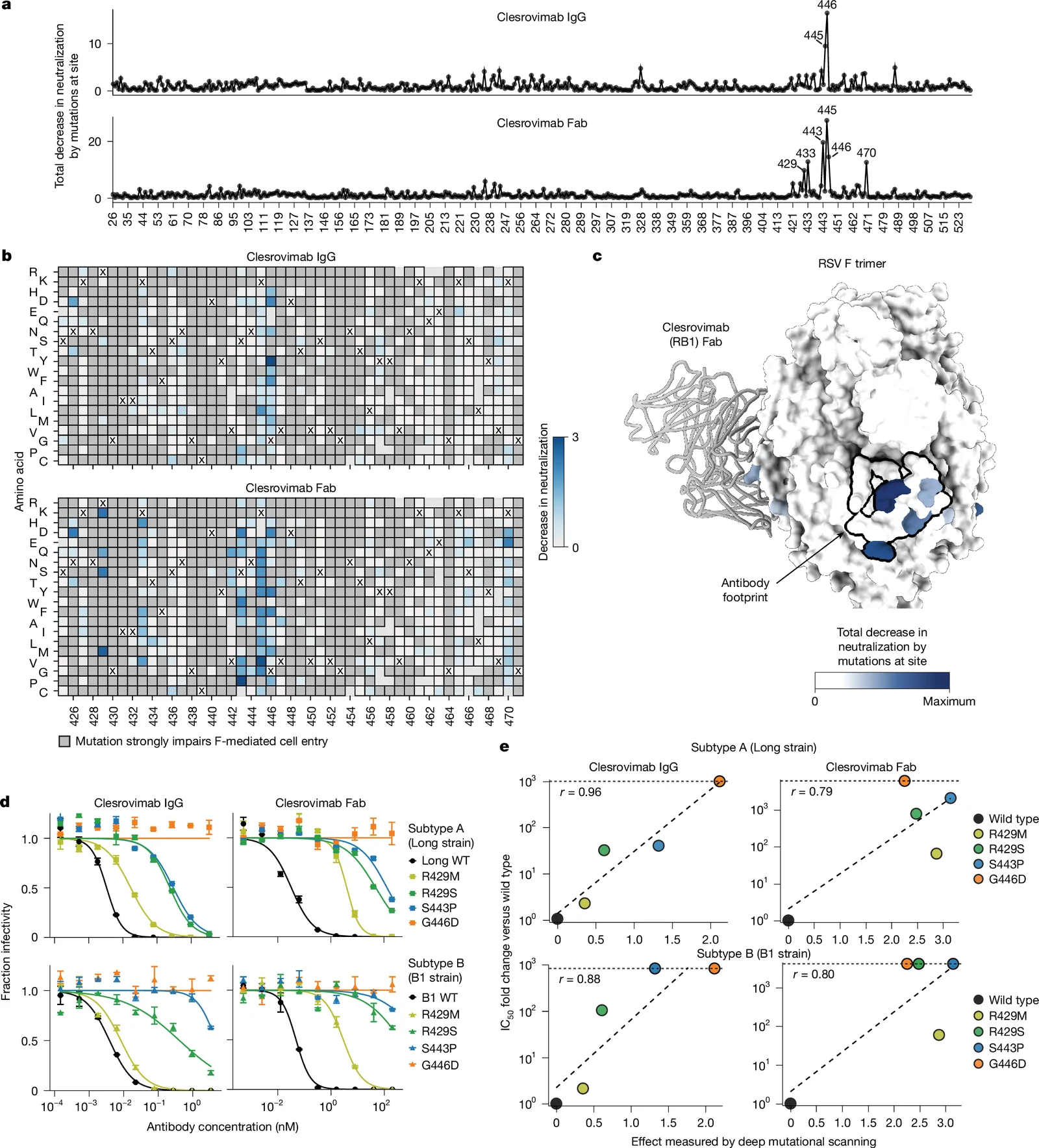
Effects of mutations to RSV F on neutralization by clesrovimab IgG and Fab. **a**, Total decrease in neutralization from all mutations at each site for clesrovimab IgG or Fab as measured by deep mutational scanning. See https://dms-vep.org/RSV_Long_F_DMS/clesrovimab_neutralization.html for interactive plots that show the effects of all mutations. **b**, Heatmaps showing effects of mutations within the epitope of clesrovimab. Each box represents a single mutation, with positive values indicating decreased neutralization. Dark grey indicates mutations that are too deleterious for cell entry to measure their effect on neutralization. Light grey shading indicates mutations that were not measured. The wild-type amino acid in the subtype A Long strain used for the deep mutational scanning at each site is indicated with an ‘x’. **c**, Prefusion RSV F trimer (PDB 6OUS, ref. ^14^) in complex with clesrovimab Fab (grey cartoon) coloured by the total effect of mutations at each site on neutralization by clesrovimab Fab. **d**, Neutralization curves for clesrovimab IgG and Fab of pseudovirus expressing F with point mutations in subtype A (Long) or subtype B (B1) background. Individual mutations were selected to span a range of effects on neutralization. **e**, Correlation of mutation effects on neutralization by clesrovimab IgG and Fab between deep mutational scanning and traditional pseudovirus neutralization assays with the indicated F mutants in subtype A (Long strain) or subtype B (B1 strain). Horizontal and vertical lines indicate limits of detection. WT, wild type.

The mutations that most affect clesrovimab neutralization occur at a subset of sites between 426–470 (Fig. 4a,b). These sites are within or near the structurally determined clesrovimab binding site (Fig. 4c and interactive plots at https://dms-vep.org/RSV_Long_F_DMS/clesrovimab_neutralization.html). Some mutations identified by our deep mutational scanning have been previously reported to affect clesrovimab (for example, 443P, 445N and 446E/R/W, refs. ^14,40^). But we also identified new mutations affecting clesrovimab, including extra mutations at sites 443, 445 and 446, as well as mutations at sites not previously associated with clesrovimab resistance (426, 429, 433 and 470) (Fig. 4b).

To validate the deep mutational scanning, we generated pseudoviruses carrying key mutations in F from both subtype A (Long strain) and B (B1 strain) and measured their neutralization by clesrovimab IgG and Fab (Fig. 4d,e). The effects of mutations on neutralization IC_50_ were highly correlated with the deep mutational scanning for both subtypes (Fig. 4e).

**Extended Data Fig. 6:**
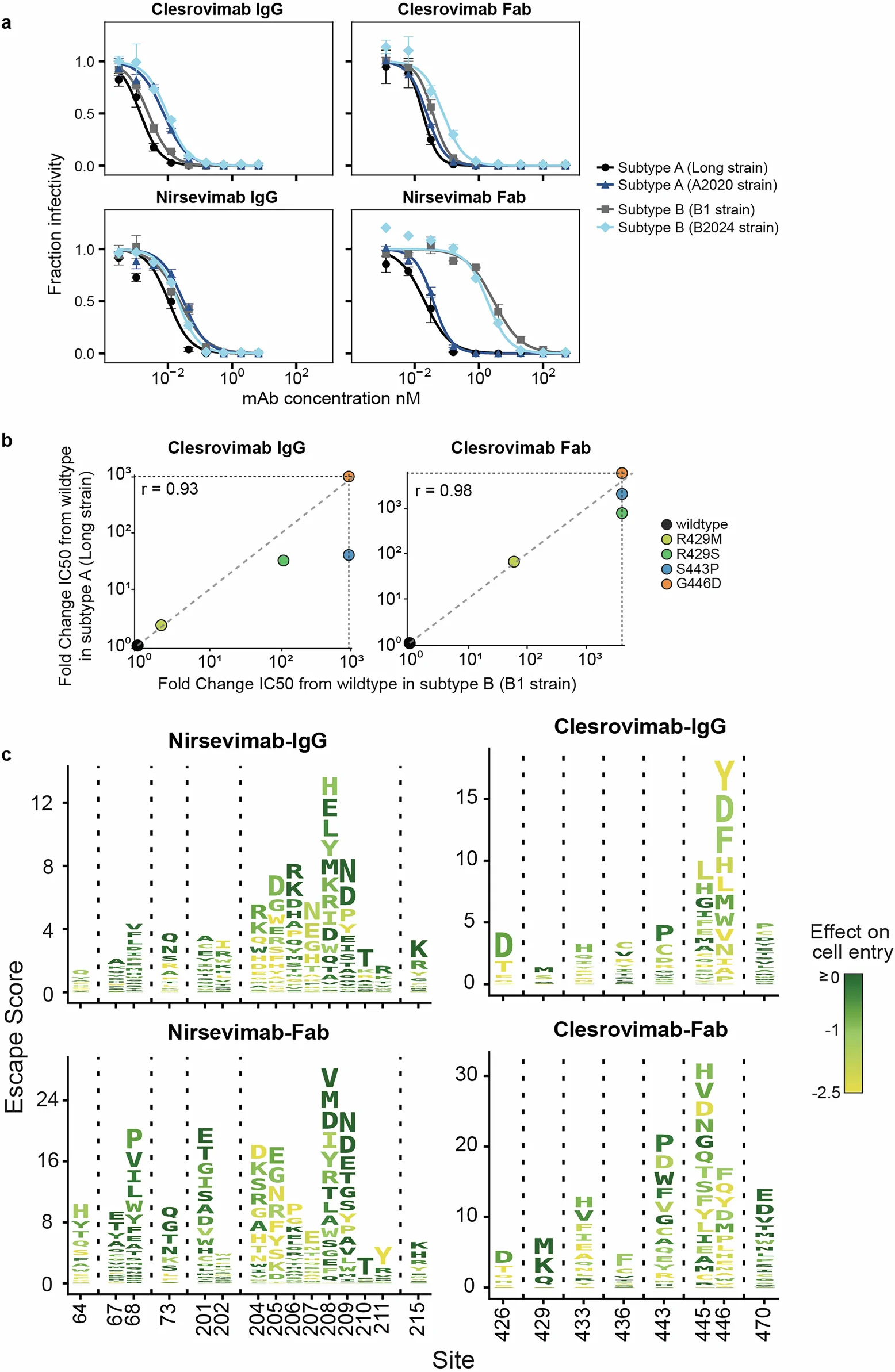
Mutations that affect clesrovimab neutralization have similar effects in subtypes A and B. (**a**) Neutralization curves for both the IgG and Fab forms of clesrovimab and nirsevimab IgG and Fab against pseudoviruses with F from two subtype A strains (the lab-adapted Long strain and the recent clinical strain A2020) and two subtype B strains (the lab-adapted B1 strain and the recent clinical B2024 strain). Points indicate the mean ± standard error of two technical replicates. (**b**) Correlation between effects of mutations on neutralization IC50 of subtype A (Long strain) or subtype B (B1 strain) F pseudoviruses in validation neutralization assays with clesrovimab IgG and Fab. Dashed gray line shows 1:1. (**c**) Mutations that reduce clesrovimab neutralization tend to be more deleterious for F’s cell entry function than mutations that reduce nirsevimab neutralization. Logo plots showing the effects of mutations at key sites on neutralization by nirsevimab or clesrovimab, measured by deep mutational scanning using IgG or Fab. The height of each letter is proportional to the reduction in neutralization mutation to that amino acid causes in the deep mutational scanning. Mutations are colored by their effect on F-mediated cell entry in the absence of antibody as measured by deep mutational scanning, with dark green indicating no effect on cell entry and yellow indicating reduced cell entry. Therefore, yellow letters represent mutations that are likely to be deleterious to viral fitness even if they reduce antibody neutralization. Note that it is impossible to measure the effect on neutralization of mutations that fully ablate cell entry, so these plots only show mutations that retain at least some moderate level of cell entry (a cell entry effect > −2.5 in our measurements). See Fig. 2 and associated hyperlinks to interactive plots for data on how all mutations at sites in the nirsevimab and clesrovimab epitopes affect cell entry function.

Mutations that moderately reduce clesrovimab neutralization had a greater impact on Fab than IgG neutralization for both subtypes (Fig. 4a,b,d; for example, R429M/S). This tendency for moderate-effect mutations to reduce Fab neutralization more than IgG neutralization is also observed for nirsevimab against subtype A but not subtype B (Fig. 3g). These observations are consistent with the biophysical model of how bivalent IgG buffering reduces the impact of mutations on IgG neutralization when the Fab affinity is sufficiently high (which is true for clesrovimab against both subtypes A and B, but for nirsevimab only against subtype A). Unlike for nirsevimab, clesrovimab Fab has similarly high neutralization potencies against both subtypes (Extended Data Fig. 6a), and the effects of mutations on clesrovimab neutralization are well correlated across subtypes (Extended Data Fig. 6b).

Our deep mutational scanning found fewer sites where mutations strongly reduced clesrovimab neutralization compared with nirsevimab. Part of the reason may be differences in functional constraint: it is only possible to measure the impact on neutralization of F mutations that retain at least some cell entry function, and mutations in the clesrovimab epitope tend to be more deleterious to cell entry than mutations in the nirsevimab epitope (Fig. 2c,d). Indeed, even among mutations that retain sufficient cell entry to measure their impact on neutralization, the ones that reduce clesrovimab neutralization tend to impair cell entry more than the ones that reduce nirsevimab neutralization (Extended Data Fig. 6c). However, there are still some mutations that reduce clesrovimab neutralization without impairing cell entry.

## Phenotypically informed RSV surveillance

**Extended Data Fig. 7:**
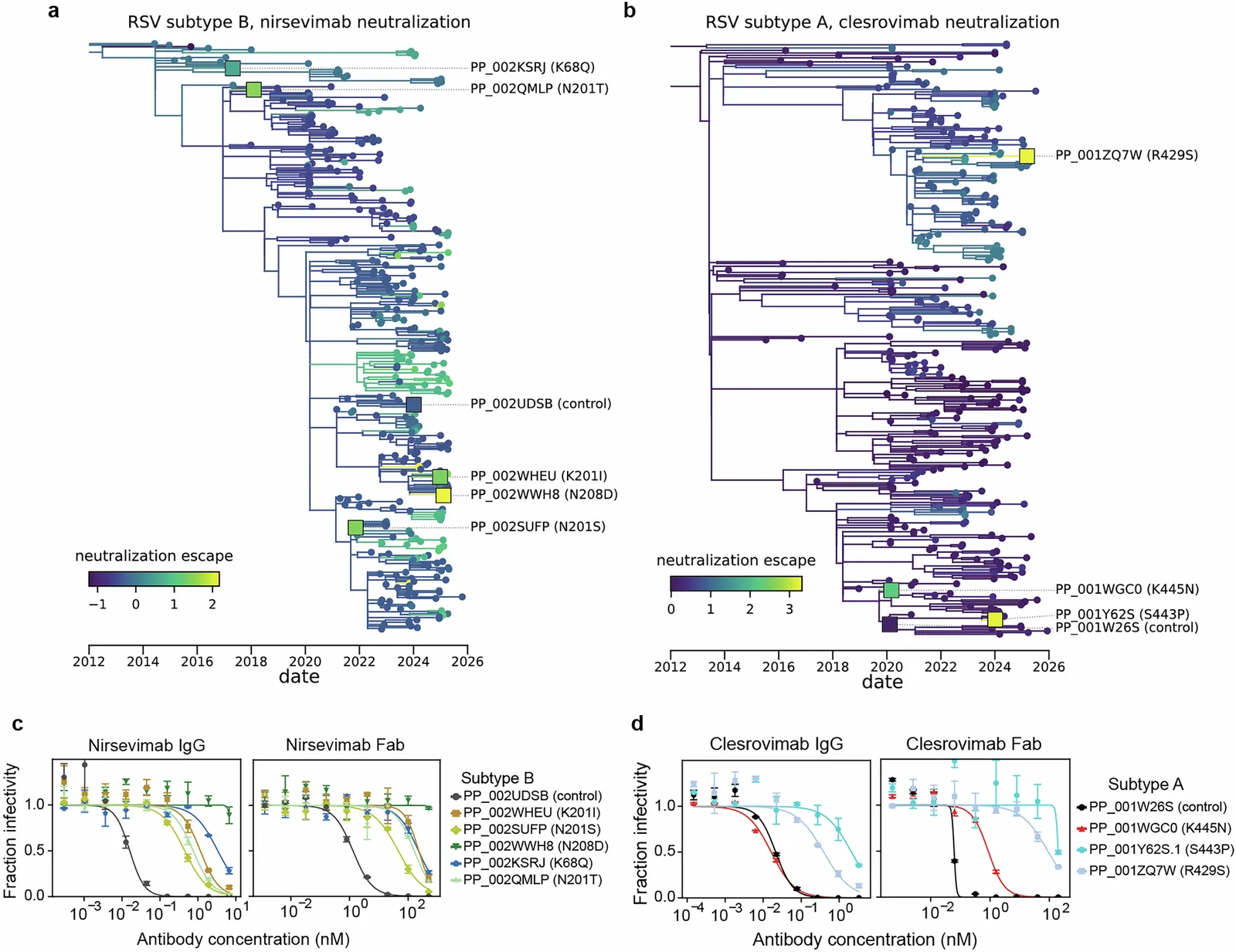
Sporadic natural RSV strains have reduced neutralization by nirsevimab or clesrovimab. (**a**) Phylogenetic trees of subtype B RSV F sequences colored by nirsevimab escape scores computed as the summed effects of all mutations as measured by the deep mutational scanning. Strains chosen for validation of nirsevimab neutralization are indicated by boxes and labeled with the Pathoplexus identifier and top resistance mutation. See https://nextstrain.org/rsv/b/F-antibody-escape/6y?c=Nirsevimab-Fab_total_escape for interactive Nextstrain trees that show more sequences and are updated in real time. (**b**) Tree of subtype A RSV F sequences colored by clesrovimab escape scores https://nextstrain.org/rsv/a/F-antibody-escape/6y?c=Clesrovimab-Fab_total_escape. (**c**) Neutralization curves showing nirsevimab IgG or Fab neutralization of pseudoviruses expressing F from the subtype B strains labeled on the tree. The strains with high escape scores all have reduced neutralization relative to a control strain with a low escape score. (**d**) Neutralization curves showing clesrovimab IgG or Fab neutralization of pseudoviruses expressing F from the subtype A strains labeled on the tree. Strains with high escape scores all have reduced neutralization relative to a control strain, except a strain (PP_001WGC0) with only a moderate escape score that shows reduced Fab not but not IgG. See Extended Data Fig. 7 for trees and validating neutralization assays for subtype A strains with nirsevimab resistance and subtype B strains with clesrovimab resistance.

**Extended Data Fig. 8:**
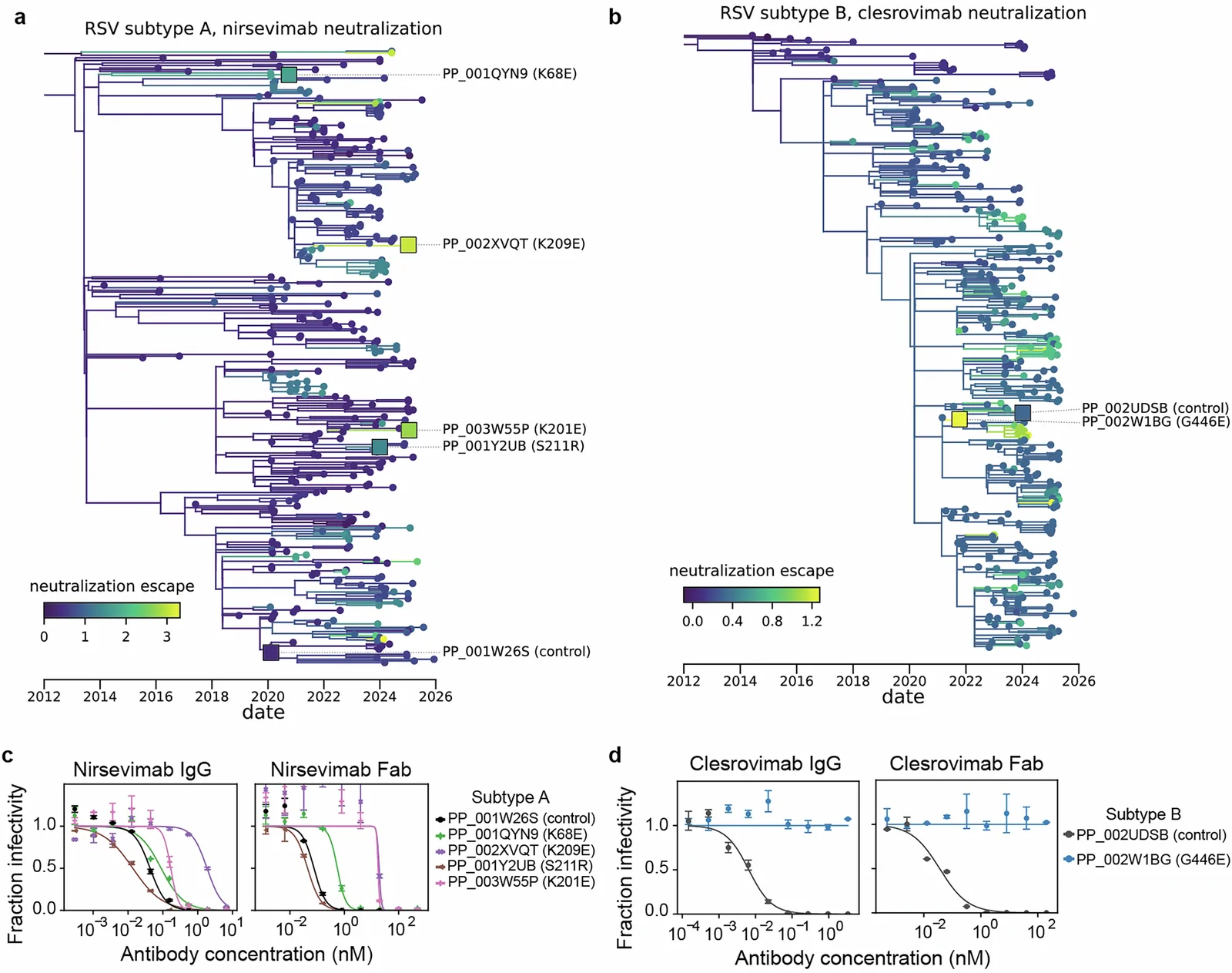
Additional phylogenetic trees and validation assays showing sporadic nirsevimab and clesrovimab resistance mutations in natural RSV sequences. This figure is like Fig. 5 except it shows (**a**) trees of subtype A RSV F with nirsevimab escape scores, (**b**) trees of subtype B RSV F with clesrovimab escape scores, (**c**) neutralization assays validating reduced neutralization of natural subtype A strains by nirsevimab, and (**d**) neutralization assays validating reduced neutralization of a natural subtype B strain by clesrovimab. Note that that subtype A strain PP_001Y2UB strain does not have reduced nirsevimab neutralization despite a high escape score driven the S211R resistance mutation (which Fig. 3d validates reduces nirsevimab neutralization in another subtype A strain); we speculate that neutralization of strain PP_001Y2UB might be affected by an epistatic interaction between S211R and a second R213S mutation it contains at a nearby site.

Now that nirsevimab and clesrovimab are in widespread use, surveillance for natural RSV strains with resistance to these antibodies is a public-health priority^3,4,6^. Although large numbers of human RSV infections are regularly being sequenced^3–6,20,41–44^, interpretation of these sequences has been limited by incomplete knowledge of which mutations affect antibody resistance. Our deep mutational scanning enables immediate assessment of all RSV sequences for antibody resistance. We calculated two escape scores for each RSV sequence: the summed effect on neutralization of all its F mutations relative to the subtype A Long strain used in the deep mutational scanning, and the effect of the single mutation that caused the greatest reduction in neutralization. We integrated these scores into Nextstrain^45^ phylogenetic trees of RSV showing the latest sequence data from Pathoplexus^46^. As Nextstrain subsamples the many available F sequences for effective visualization, we also created new builds that ensure the subsampling includes sequences with high escape scores. Interactive phylogenetic trees that can be coloured by the escape scores and are updated to include the latest available sequences are at https://nextstrain.org/rsv/b/F-antibody-escape/6y?c=Nirsevimab-Fab_total_escape. Static images based on further subsampled versions of these trees are in Extended Data Figs. 7a,b and 8a,b.

Sequences with high nirsevimab or clesrovimab escape scores were rare (less than 1% of all sequences) and distributed across the phylogenetic trees, suggesting resistance mutations have arisen sporadically and not undergone sustained spread (Extended Data Figs. 7a,b and 8a,b). However, the fact that strains with resistance have emerged and transmitted in humans to a limited extent underscores the importance of continued surveillance.

To validate that natural sequences with high escape scores had reduced neutralization, we generated RSV pseudoviruses expressing the F proteins from strains with high escape scores for nirsevimab or clesrovimab. Nearly all strains with high escape scores had reduced neutralization relative to control recent strains with low escape scores (Extended Data Figs. 7c,d and 8c,d). The natural strains with reduced neutralization include ones with resistance mutations newly identified by our deep mutational scanning (for example, K201I and K201E for nirsevimab, and R429S for clesrovimab). A caveat is that the escape scores assume resistance can be predicted from the additive effects of a strain’s mutations; our validation assays show that assumption usually holds, but we did identify one subtype A strain with a high nirsevimab escape score that did not have reduced neutralization, possibly due to an epistatic interaction of its S211R resistance mutation with a nearby R213S mutation (Extended Data Fig. 8c).

## Historical and candidate antibodies

We next used deep mutational scanning to characterize mutations that affect neutralization by five more antibodies of historical or potential future clinical relevance.

**Fig. 5:**
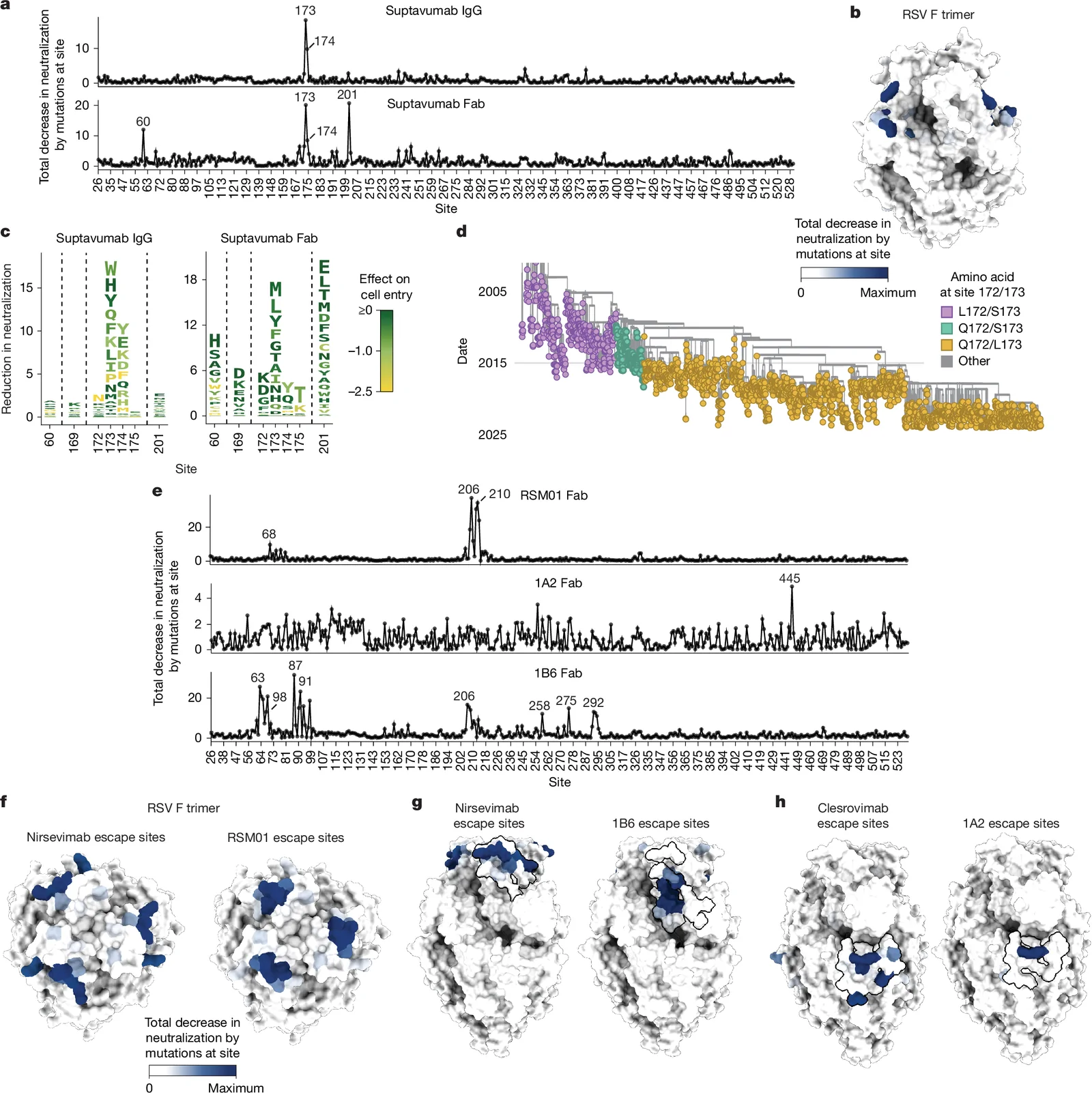
Effects of mutations to RSV F on neutralization by the historical failed antibody suptavumab and three candidate future clinical antibodies (RSM01, 1A2, 1B6). **a**, Total decrease in neutralization from all mutations at each site for suptavumab IgG or Fab as measured by deep mutational scanning. See https://dms-vep.org/RSV_Long_F_DMS/suptavumab_neutralization.html for interactive plots that show the effects of all mutations. **b**, Prefusion RSV F trimer (PDB 5UDC, ref. ^13^) coloured by the total effect of mutations at each site on neutralization by suptavumab Fab. **c**, Effects on suptavumab neutralization of mutations at key sites. The height of each letter is proportional to the reduction in neutralization caused by a mutation to that amino acid. Mutations are coloured by their effect on F-mediated cell entry in the absence of antibody (dark green indicates no effect on cell entry and yellow indicates reduced cell entry). **d**, Phylogenetic trees of subtype B RSV F sequences coloured by amino acid identities at sites 172 and 173. **e**, Total decrease in neutralization from all mutations at each site for RSM01, 1A2 or 1B6 Fabs. See https://dms-vep.org/RSV_Long_F_DMS/ for interactive plots that show effects of all mutations. **f**, Prefusion RSV F trimer (PDB 5UDC, ref. ^13^) coloured by the total effect of mutations at each site on neutralization by RSM01 or nirsevimab Fab. **g**, Prefusion RSV F trimer (PDB 9LLY, ref. ^52^) coloured by the total effect of mutations at each site on neutralization by 1B6 or nirsevimab Fab. The structurally determined antibody-binding footprint is outlined. **h**, Prefusion RSV F trimer (PDB 9LLY, ref. ^52^) coloured by the total effect of mutations at each site on neutralization by clesrovimab or 1A2.

Suptavumab failed a phase 3 trial run from 2015 to 2017 due to lack of efficacy against subtype B^2^. Our deep mutational scanning shows that the mutations that most reduce suptavumab neutralization are at sites 173 and 174 (Fig. 5a–c). Starting in 2015, L172Q and S173L became prevalent in subtype B sequences (Fig. 5d), and the reduced ability of suptavumab to neutralize strains with these two mutations led to the failed clinical trial^2^. Our deep mutational scanning shows that S173L reduces suptavumab neutralization while having a minimal adverse impact on F’s cell entry function (Fig. 5c). Notably, S173L had already spread to roughly half of subtype B sequences by the time the suptavumab phase 3 clinical trial began in November 2015 (ref. ^2^), so suptavumab’s failure could have been anticipated if our deep mutational scanning had been available in advance.

**Extended Data Fig. 9:**
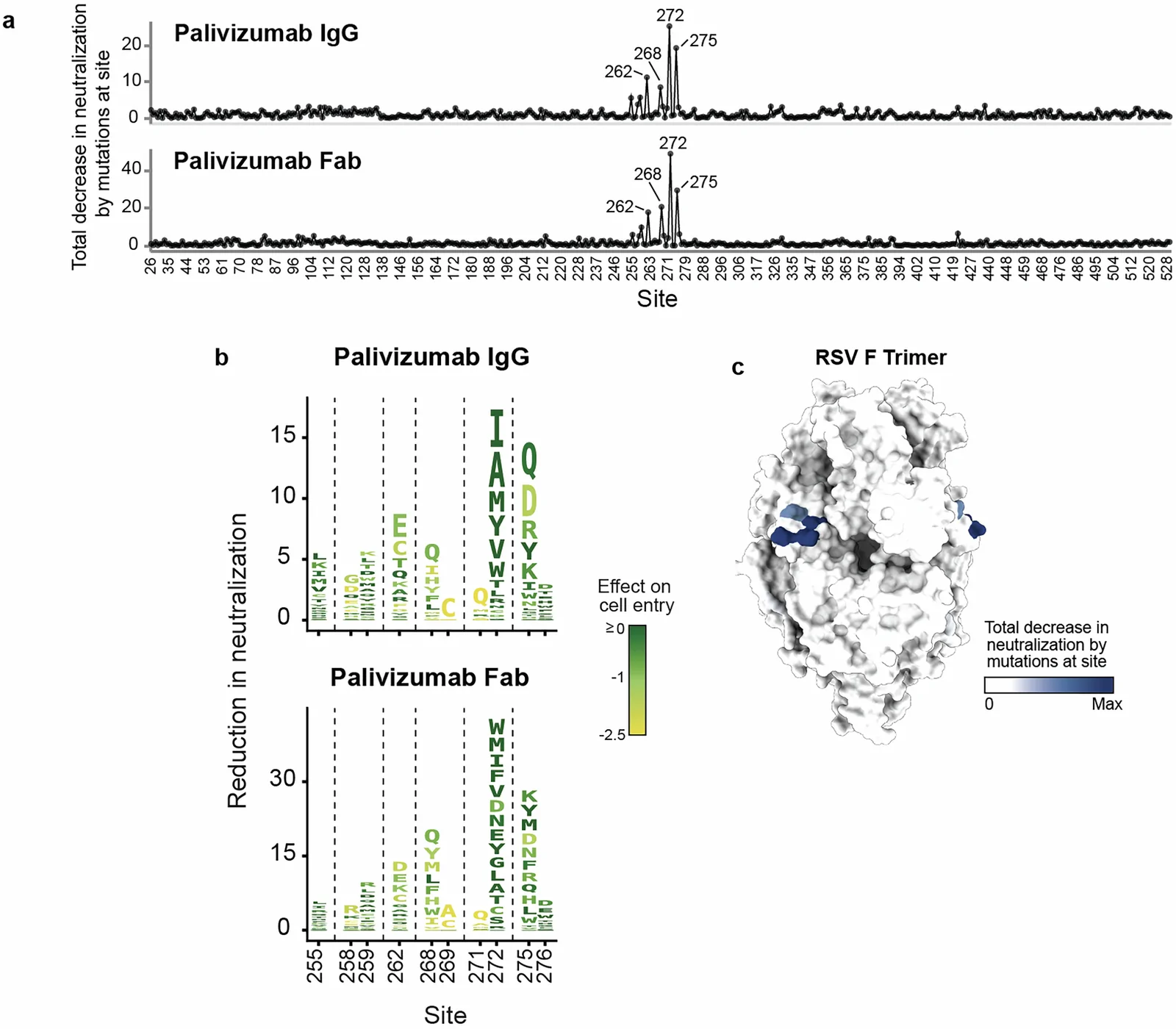
Effects of F mutations on palivizumab neutralization. (**a**) Total decrease in neutralization from all mutations at each site for palivizumab IgG or Fab as measured by deep mutational scanning. See https://dms-vep.org/RSV_Long_F_DMS/palivizumab_neutralization.html for interactive plots that show the effects of all mutations. (**b**) Logo plots showing the effects of mutations at key sites on neutralization by palivizumab measured by deep mutational scanning using IgG or Fab. The height of each letter is proportional to the reduction in neutralization a mutation to that amino acid causes in the deep mutational scanning. Mutations are colored by their effect on F-mediated cell entry in the absence of antibody as measured by deep mutational scanning, with dark green indicating no effect on cell entry and yellow indicating reduced cell entry. Therefore, yellow letters represent mutations that are likely to be deleterious to viral fitness even if they reduce antibody neutralization. It is impossible to measure the effect on neutralization of mutations that fully ablate cell entry, so these plots only show mutations that retain at least some moderate level of cell entry (a cell entry effect > −2.5 in our measurements). (**c**) Pre-fusion RSV F trimer (PDB 5UDC^13^) colored by the total effect of mutations at each site on neutralization by palivizumab Fab.

Palivizumab is an older monoclonal antibody previously recommended only for high-risk (for example, preterm) infants^47^ that has now largely been replaced by nirsevimab and clesrovimab as they have a longer half-life and are more potent. Our deep mutational scanning showed palivizumab neutralization is strongly reduced by mutations at a subset of sites from 255 to 277 (Extended Data Fig. 9), consistent with previous work showing mutations at some of these sites cause palivizumab resistance^48–51^.

**Extended Data Fig. 10:**
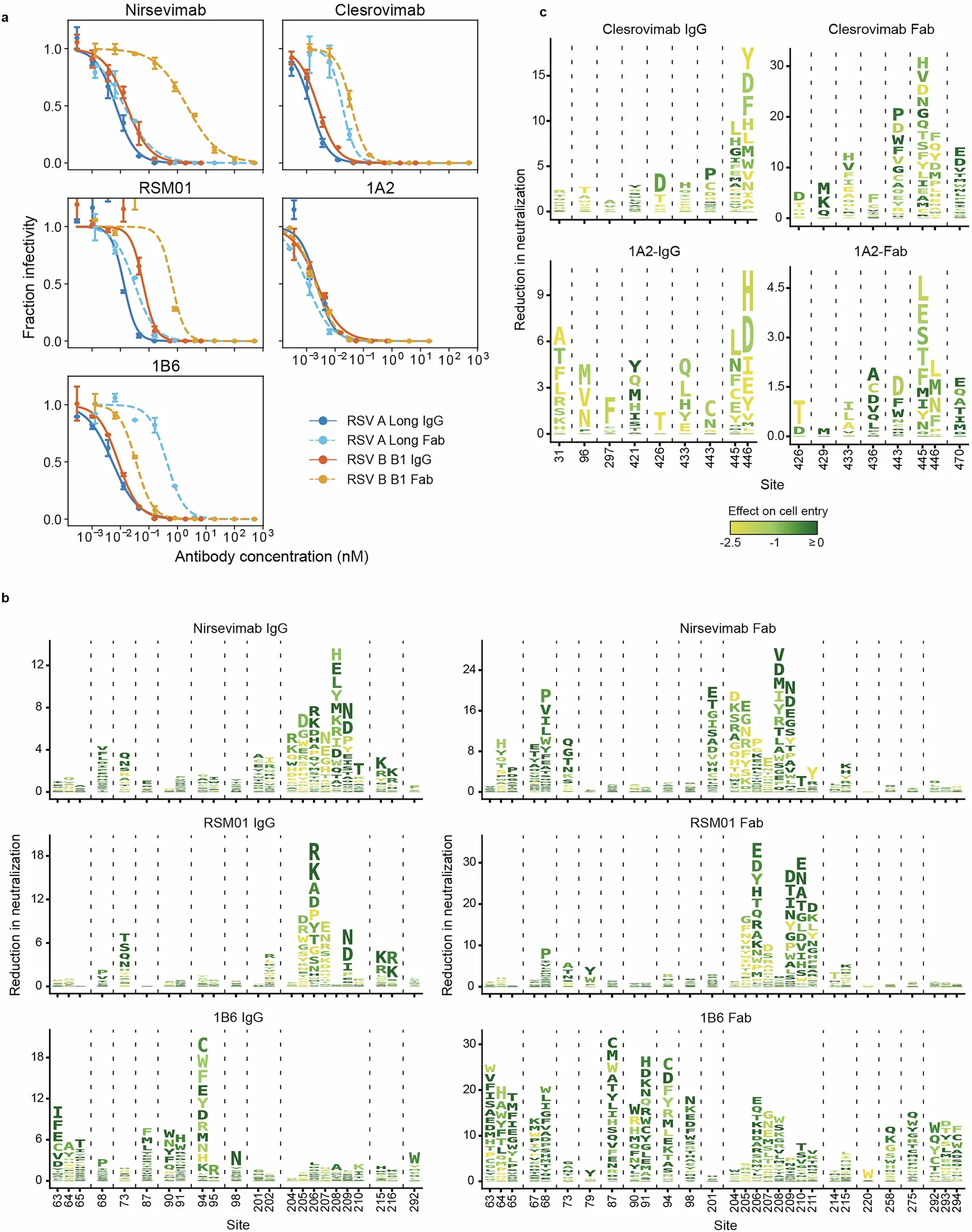
Comparison of mutational effects on neutralization by current (nirsevimab, clesrovimab) or candidate future (RSM01, 1A2, 1B6) clinical antibodies. (**a**) Neutralization curves for both the IgG and Fab forms of nirsevimab, RSM01, 1B6, clesrovimab, and 1A2 against pseudoviruses with F from the subtype A Long strain or subtype B B1 strain. Data for clesrovimab IgG and Fab are re-plotted from Extended Data Fig. 6a to facilitate comparison to other antibodies plotted here. (**b**) Logo plots showing the effects of mutations at key sites on neutralization by apex-targeting antibodies (nirsevimab, RSM01 and 1B6), or (**c**) lateral face-targeting antibodies (clesrovimab and 1A2) as measured by deep mutational scanning using IgG or Fab. The height of each letter is proportional to the reduction in neutralization a mutation to that amino acid causes in the deep mutational scanning. Mutations are colored by their effect on F-mediated cell entry in the absence of antibody as measured by deep mutational scanning, with dark green indicating no effect on cell entry and yellow indicating reduced cell entry. Therefore, yellow letters represent mutations that are likely to be deleterious to viral fitness even if they reduce antibody neutralization. It is impossible to measure the effect on neutralization of mutations that fully ablate cell entry, so these plots only show mutations that retain at least some moderate level of cell entry (a cell entry effect > −2.5 in our measurements).

We next characterized three anti-RSV antibodies that are candidates for future clinical use. RSM01 has been suggested for use in low-income and middle-income countries where RSV is a leading cause of infant mortality^11,22^, whereas 1A2 and 1B6 target different epitopes on F and have been proposed as components of an antibody cocktail^52^. The IgGs of all three antibodies potently neutralized pseudoviruses with either a subtype A or B F with IC_50_values less than 0.1 nM, as do the existing clinical antibodies clesrovimab and nirsevimab (Extended Data Fig. 10a). However, the Fab of the apex-targeting antibodies (nirsevimab, RSM01 and 1B6) had substantially differing potencies between subtypes: nirsevimab and RSM01 Fabs were more potent against subtype A than B, whereas 1B6 Fab was more potent against subtype B than A (Extended Data Fig. 10a). The Fabs of the lateral-face-targeting antibodies clesrovimab and 1A2 were similarly potent against both subtypes (Extended Data Fig. 10a). Notably, Fab and IgG of 1A2 had similarly high neutralization potencies (Extended Data Fig. 10a); probably because this antibody’s exceptionally high affinity for F^52^, bivalent IgG buffering confers no further neutralization advantage over the Fab, although under the biophysical model presented earlier in this paper it is still expected to confer increased IgG robustness to F mutations.

Deep mutational scanning showed that mutations reducing RSM01 neutralization occur at a subset of sites spanning residues 68–80 and 202–216 (Fig. 5e,f). Although RSM01 targets a similar region of prefusion F to nirsevimab, there is only partial overlap of the mutations that affect neutralization by these antibodies. For example, mutations at sites 201, 204 and 208 have substantially larger effects on nirsevimab than RSM01 neutralization (Figs. 3a,b and 5e,f and Extended Data Fig. 10b). Consistent with these results, a recent study that serially passaged replication-competent RSV in the presence of RSM01 selected an I206T/N262Y double mutant with partial antibody resistance^22^, and our deep mutational scanning identifies I206T as reducing RSM01 neutralization.

The two antibodies comprising the candidate antibody cocktail were affected by largely distinct sets of mutations. The mutations that reduce neutralization by 1B6 are near the apex of F, but at largely distinct sites from where mutations affect the other apex-targeting antibodies nirsevimab and RSM01 (Fig. 5e,g and Extended Data Fig. 10b). The sites where mutations affect 1A2 neutralization substantially overlap with sites that affect clesrovimab neutralization, although there are few single mutations that strongly reduce 1A2 neutralization probably because its exceptionally high affinity for F confers substantial robustness to mutations (Fig. 5e,h and Extended Data Fig. 10c). The mutations that reduce neutralization by all three apex-targeting antibodies (nirsevimab, RSM01 and 1B6) are often better tolerated for F’s cell entry function than mutations that reduce neutralization by the lateral-face-targeting antibodies (clesrovimab and 1A2) (Extended Data Fig. 10b,c).

## Discussion

We have used pseudovirus deep mutational scanning to measure how nearly all mutations to RSV F affect neutralization by clinically relevant antibodies. Previous work to identify resistance mutations, including for FDA-required resistance analyses^19,40,53^, has serially passaged authentic RSV in the presence of antibody^5,14,22^. Our strategy has two advantages over this classical approach: it characterizes all mutations rather than just identifying whichever ones stochastically arise during viral passage, and it does not generate mutants of actual replicative virus (pseudoviruses can only undergo a single round of cell entry). On the other hand, serial passaging can uncover resistance mediated by combinations of mutations, whereas deep mutational scanning only measures the effects of single mutations. However, in practice, our deep mutational scanning identified all resistance mutations previously reported by serial-passage studies as well as several new mutations, some of which are present in natural RSV sequences.

At first blush, the fact that we measured effects of mutations to F from only a single subtype A strain might seem a major limitation, especially given the perplexing previous observation that nirsevimab resistance is more prevalent in subtype B^3–6,16–20^. But a key insight of our work is that subtype-dependent effects of mutations on nirsevimab neutralization are largely explained by biophysically modelling how bivalent IgG binding buffers mutations when Fab potency is sufficiently high. Because nirsevimab Fab has higher affinity for subtype A than subtype B RSV F^19,26^, mutations that moderately reduce Fab neutralization of both subtypes only reduce IgG neutralization of subtype B. Therefore, we measured how F mutations affected both IgG and Fab neutralization, and showed that the Fab measurements identified mutations that reduced IgG neutralization of subtype B despite having minimal impact on IgG neutralization of subtype A. This biophysical principle extends beyond RSV and nirsevimab, and implies that mutations that modulate Fab potency without affecting IgG neutralization can nonetheless alter the impact of subsequent mutations on IgG neutralization. However, other more specific mechanisms can also shape how a mutation affects different strains, and so the fact that our measurements were performed in a single genetic background remains a caveat.

An immediate benefit of our work is enabling real-time assessment of natural RSV sequences for antibody resistance. Because anti-RSV antibodies are of such public-health importance, genomic surveillance of RSV has become a main priority^4,6,20,21^: there are now more than 60,000 sequences in public databases^46^ and the number is rapidly growing. However, interpretation of these sequences has been limited by the fact that previous experimental work only characterized how a small fraction of all F mutations affect antibody neutralization^3–6,16–20,22,40^. We used our deep mutational scanning data to score all available sequences and identify sporadic nirsevimab or clesrovimab resistance, in some cases mediated by mutations not previously reported to affect those antibodies. We implemented this resistance-scoring system into real-time Nextstrain phylogenetic trees and created a web interface with our experimental data (https://dms-vep.org/RSV_Long_F_DMS). These resources will enable continued assessment for resistance to current clinical antibodies as well as new ones under development such as RSM01, 1B6 and 1A2 (refs. ^11,22,52^).

Fortunately, resistance to nirsevimab and clesrovimab is rare at present. Resistance could remain rare, or could eventually spread, as happened with suptavumab resistance in RSV subtype B in 2015–2016 (ref. ^2^) or oseltamivir (Tamiflu) resistance in H1N1 influenza in 2008 (ref. ^54^). Resistance can spread incidentally if resistance mutations also enhance viral fitness by an unrelated mechanism such as increasing transmissibility or reducing recognition by population immunity^55,56^; such incidental spread probably explains why suptavumab resistance emerged before phase 3 clinical trials of that antibody. We previously showed that some nirsevimab resistance mutations mildly reduce neutralization of RSV by polyclonal human sera^31^, raising the possibility that they could confer a small incidental benefit to the virus if they do not adversely affect any other aspects of fitness. In addition, now that nirsevimab and clesrovimab are in clinical use, there is also direct evolutionary pressure for viral resistance that enables infection of treated individuals. However the infants receiving these antibodies are just a small fraction (less than 1%) of all people^57,58^. Resistance would therefore confer a real but small advantage, and so such a strain would spread only if the resistance mutations do not impair other aspects of transmissibility by even a small amount^56^. It is impossible to experimentally measure RSV transmissibility with such precision; although our deep mutational scanning measured how mutations affect F’s cell entry function, these experiments have a precision far worse than 1% and cell entry is only one of several F properties that contribute to transmissibility. Therefore, the best that can be done at present is to monitor for the real-world spread of resistance while continuing to develop new antibodies with distinct resistance profiles: goals that our study helps advance.

## Methods

### Biosafety and biosecurity

We performed all experiments with pseudotyped lentiviral particles at biosafety level 2 (ref. ^59^). These pseudoviruses do not encode any other viral proteins other than RSV F and therefore can only undergo a single round of cell entry, and so they are not fully replicative infectious agents capable of causing disease. The other viral proteins needed for the formation of pseudotyped lentiviral particles (RSV G, and the lentiviral Gag/Pol, Tat and Rev) were provided during pseudovirus production by transfection of four separate helper plasmids and are not encoded in the viral genome. Therefore, this study did not generate any mutants of fully replicative biological agents capable of causing disease.

Our study quantifies how all single mutations to F affect neutralization by antibodies, including those in clinical use. However, RSV is already evolving under widespread pressure from these antibodies in the human population, with resistant strains regularly identified in breakthrough infections^3,4,6^. In the past, escape mutations have been identified from these breakthrough infections or by passaging authentic RSV virus in the presence of antibodies^3–6,14,22,26^. Our experiments systematically measure the effects of mutations outside the context of pathogenic virus, and therefore enable informed surveillance of already ongoing evolution on clinical antibodies, as well as informing the design of new antibodies more resilient to viral resistance.

### Antibodies

The RSV monoclonal antibodies nirsevimab^13,19^, clesrovimab^14,40^, palizumab^47^, suptavumab^2^, RSM01 (ref. ^11^), 1A2 (ref. ^52^) and 1B6 (ref. ^52^) were produced by GenScript as human IgG1 kappa isotypes and Fabs. Sequences were obtained from the referenced publications, structures in the Protein Data Bank (PDB) or original patents. Palivizumab originated from patent US6955717B2. See https://github.com/dms-vep/RSV_Long_F_DMS/tree/main/supplemental_data/antibody-sequences for antibody amino acid sequences. The sequence of RSM01 was shared by the Gates Medical Research Institute.

### Plasmids and primers

All plasmid and primer sequences used in this study are available via GitHub at https://github.com/dms-vep/RSV_Long_F_DMS/tree/main/supplemental_data. All primers were obtained from Integrated DNA Technologies.

### Cell line handling

All cell lines (293T, sourced from the American Type Culture Collection; 293T-TIM1, ref. ^31^ and Takara Lenti-X 293T) were cultured in D10 media (Dulbecco’s Modified Eagle Medium supplemented with 10% heat-inactivated fetal bovine serum, 2 mM l-glutamine, 100 U ml^−1^ penicillin and 100 mg ml^−1^ streptomycin) and cultured at 37 °C with 5% CO_2_.

### Design of deep mutational scanning libraries of RSV F

We created pseudovirus libraries containing nearly all possible single amino acid mutations to the ectodomain of the RSV F protein. We used unmutated parental F from the Long strain of RSV, which is a subtype A, laboratory-adapted strain isolated in the 1950s (ref. ^32^). We codon optimized this sequence using the GenSmart codon optimization tool offered by GenScript and removed four amino acids from the cytoplasmic tail to increase pseudovirus titres^31^. We used a lentiviral backbone that contains an extended Gag sequence to enhance packaging of lentiviral genomes as previously described in refs. ^60,61^. The plasmid map for the lentiviral backbone with codon-optimized RSV F sequence is available from GitHub at https://github.com/dms-vep/RSV_Long_F_DMS/blob/main/supplemental_data/plasmids/4821_v5lp_phru3_forind-extgag_RSV_Long_F_GS4Opt_4aaCTdel.gb.

We aimed to include all single amino acid mutations in the RSV F ectodomain (residues 26–529; 504 × 19 = 9,576 mutations). We also included 30 stop codons located at alternating positions from the start of the ectodomain as a negative control for cell entry measurements. We ordered a site-saturation variant library with these criteria from Twist Biosciences. The final Twist quality control report for the library is available through GitHub at https://github.com/dms-vep/RSV_Long_F_DMS/blob/main/supplemental_data/Final_QC_Report_Q-392996_VariantProportion.csv.

### Cloning of deep mutational scanning plasmid libraries of RSV F

The RSV F library was designed to have all mutations to the ectodomain of F. However, 268 mutations were missing from the library produced by Twist. We added the missing mutations and also over-represented mutations in the nirsevimab binding site (amino acid residues 62–69 and 196–212) because we wanted to ensure inclusion and measurement of effects of all possible mutations in the epitope. We aimed to clone a ‘spike-in’ plasmid library that contains these missing mutations and over-representation of mutations in the nirsevimab binding site using a mutagenesis PCR protocol^62–64^. We designed NNS (where N is any of the four nucleotide bases and S is cytosine or guanine, representing 32 codons that encode all 20 amino acids and a stop codon) primers for the nirsevimab-targeting sites with CodonTilingPrimers (https://github.com/jbloomlab/CodonTilingPrimers) and primers for missing mutations with TargetedTilingPrimers (https://github.com/jbloomlab/TargetedTilingPrimers). Forward and reverse primer pools were created by combining either forward or reverse NNS and targeted mutation primers at an equal molar ratio per codon for a final concentration of 5 µM. Linear template of RSV F was made by digesting the lentiviral backbone with codon-optimized RSV F sequence with NotI-HF and NdeI. PCR mutagenesis was then performed as described previously in ref. ^30^ with the only difference being that seven PCR cycles were used for the mutagenesis PCR to reduce the resulting number of multi-mutants. After the PCR, the product was digested using DpnI for 20 min at 37 °C to remove any leftover template. The PCR mutagenesis to generate the ‘spike-in’ was performed in duplicate, once for library A and once for library B.

We then barcoded the library pools made by Twist Biosciences independently in two separate reactions to make library A and B, respectively. These two biological replicates were handled separately for all subsequent experimental steps. The barcoding was performed as in ref. ^30^ using primers containing a random 16 nucleotide sequence downstream of the RSV F stop codon. The only difference from ref. ^30^ is that only 5 ng of template was used. The two pools of mutagenized RSV F for the spike-in were also barcoded separately. In all there were four separate barcoding PCR reactions. The lentiviral backbone (4016_V5LP_pHrU3_ForInd-Extgag_mcherry)^61^ was digested with MluI-HF and XbaI at 37 °C for 45 min then gel purified and purified using AMPure XP beads (Beckman Coulter, A63881). The barcoded libraries were cloned into the lentiviral backbone at a 1:2 insert to vector ratio in a HiFi assembly for 1 h at 50 °C. The HiFi product was purified with AMPure XP beads and eluted in molecular grade water. The purified products were transformed into 10-beta electrocompetent cells (New England Biolabs, C3020K) using a BioRad MicroPulser Electroporator, shocking at 2 kV for 5 ms. Ten reactions of electroporation were performed per barcoded Twist library and two reactions per mutagenized spike-in library. Following the 1 h recovery at 37 °C, transformed cells were spun out of SOC (super optimal broth) and pooled and cultured in 150 ml of Luria-Bertani medium for each Twist library and 100 ml of Luria-Bertani medium for each spike-in library with ampicillin overnight at 37 °C in a shaking incubator. Plasmids were extracted using the QIAGEN HiSpeed Plasmid Maxi Kit (QIAGEN, 12662).

Corresponding replicates of the Twist plasmid libraries and spike-in plasmid libraries were combined at a 1:1.25 Twist to spike-in molar ratio per codon because long-read PacBio sequencing of the plasmid libraries revealed this ratio results in the most even distribution of mutants in the combined libraries.

### Production of cell-stored deep mutational scanning libraries

Cells storing the deep mutational scanning libraries as single integrated RSV F containing genomes per cell were produced as described in ref. ^30^ with a few changes (Extended Data Fig. 1b). VSV-G pseudotyped lentiviruses were produced by transfecting four 10-cm dishes of 293T cells with the lentiviral backbone containing the barcoded RSV F libraries per library (5 μg per dish), lentiviral Gag/Pol, Tat and Rev helper plasmids (1.25 μg per dish, AddGene: HDM-tat1b product ID 204154, pRC-CMV-Rev1b product ID 20413, HDM-Hgpm2 product ID 204152)^30^ and a VSV-G expression plasmid (1.25 μg per dish, AddGene pMD2.G product ID 12259) using BioT (Bioland Scientific, B01-02).

Whereas previous deep mutational scanning studies have integrated into a specific clone of 293T-rtTA cells that were previously found to yield good pseudovirus titres for other viral entry proteins^30^, we found that this clone was unable to generate high titre RSV pseudovirus. We tested many clones of 293T-rtTA cells but found that the highest titre RSV pseudovirus produced from singly integrated cells was produced from Takara’s Lenti-X 293T Cell Line (632180). This clonal 293T cell line does not overexpress rtTA. Our lentiviral backbone includes a doxycycline-inducible promoter; in other deep mutational scanning studies that use 293T-rtTA cells and a similar backbone, doxycycline is added to induce expression from the lentiviral backbone^30^. We found that for production from RSV F cell-stored libraries, pseudovirus titres were sufficiently high when expression was induced only from Tat at transfection. We found no further benefit with transfection of rtTA and addition of doxycycline when producing pseudovirus. Therefore, we used Takara’s Lenti-X 293T Cell Line for the cell-stored library. To make the cell-stored library, the VSV-G pseudotyped viruses were used to infect Takara’s Lenti-X 293T Cell Line at an infection rate of less than 1% so that most transduced cells would receive only a single integrated genome. Transduced cells were selected using puromycin so that the final population of cells contained an integrated genome encoding a single barcoded variant of RSV F. These cells were expanded and frozen with at least 2 × 10^7^ cells per aliquot and stored in liquid nitrogen for later use.

### Rescue of F and VSV-G expressing pseudovirus libraries

To rescue F-expressing pseudoviruses from the integrated cells, 100 million cells were plated in 5-layer flasks. The following day, each flask was transfected with 118.75 μg of each helper plasmid (AddGene: HDM-tat1b product ID 204154, pRC-CMV-Rev1b product ID 20413, HDM-Hgpm2 product ID 204152) and 18.75 μg of RSV G expression plasmid (AddGene: HDM_RSV_Long_G_31AACTdel product ID 237350). Xfect transfection reagent (631318) was used according to the manufacturer’s instructions (112.5 μl of Xfect transfection reagent and 7.5 ml of buffer per 5-layer flask). At 48 h after transfection, the supernatant was filtered through a 0.45-μm SFCA Nalgene 500-ml Rapid-Flow filter unit (09-740-44B). Filtered supernatant was then concentrated by ultracentrifugation with a 20% sucrose cushion in Hank’s buffered saline solution (Fisher, 14025092) at 100,000*g* for 1 h at above 20 °C. The pseudoviruses pelleted to the bottom of the cushion, after disposal of the supernatant, pellets were resuspended in D10. Aliquots of concentrated F-expressing pseudoviruses were flash frozen as previously described in ref. ^31^ and stored at −80 °C for use in downstream selection experiments.

To rescue VSV-G expressing pseudoviruses from integrated cells, 100 million cells were plated in 5-layer flasks. The following day, each flask was transfected with 37.5 μg of each helper (AddGene: HDM-tat1b product ID 204154, pRC-CMV-Rev1b product ID 20413, HDM-Hgpm2 product ID 204152) and VSV-G expression plasmid (AddGene pMD2.G product ID 12259). BioT transfection reagent was used according to the manufacturer’s instructions. At 48 h after transfection, the supernatant was filtered through a 0.45-μm SFCA Nalgene 500-ml Rapid-Flow filter unit and concentrated using Lenti-X concentrator (Takara, 631232) at a 1:3 virus to concentrator ratio, incubating at 4 °C for 3 h and spinning at 1,500*g* and 4 °C for 45 min. Following centrifugation, supernatant was discarded and viral pellets resuspended in D10. Aliquots of concentrated VSV-G expressing pseudoviruses were frozen at −80 °C for later use.

### Long-read PacBio sequencing for variant-barcode linkage

To link the barcode sequences with the mutations found in RSV F we used long-read PacBio sequencing of the lentiviral genomes in pseudoviruses made from the cell-stored libraries. We performed PacBio barcode-mutation linking after generating the singly integrated cell libraries because template switching of the pseudodiploid lentiviral genome during reverse transcription can alter barcode-variant pairings relative to the original plasmid pool. Sequencing the integrated library ensures that barcode-variant linkages reflect those present in the actual integrated provirus. This process has been described in ref. ^30^. For this library we made a few alterations. A total of 1 × 10^6^ 293T-TIM1 cells were plated in each well of six-well plates coated with poly-l-lysine. The next day, 30 million transducing units (TU) of VSV-G expressing library pseudoviruses were used to infect cells (six wells for each library at 5 million TU per well). At 12 h following infection, the non-integrated reverse-transcribed lentiviral genomes were recovered by miniprepping the 293T-TIM1 cells as described in ref. ^30^. Amplicons for long-read sequencing of the miniprepped genomes were prepared by following a previously described approach in refs. ^30,64^. The PCR reactions for each library were combined and amplicon length was verified by TapeStation before sequencing. Libraries were each sequenced on a single-molecule real-time sequencing cell with a video time length of 30 h on a PacBio Sequel IIe sequencer. To maximize identification of all variants present, each library was sequenced a second time.

We used the dms-vep-pipeline-3 package (https://github.com/dms-vep/dms-vep-pipeline-3), to process sequencing data. To link specific mutations with each barcode, PacBio circular consensus sequences were aligned to the unmutated RSV F reference sequence using the alignparse package^65^. Reads were filtered out if they aligned poorly, had a higher than expected number of mutations in the unmutated regions, did not contain a barcode or were the result of strand exchange. Consensus sequences for each barcode and/or variant sequence were constructed using alignparse, while requiring a minimum of three circular consensus sequence (CCS) reads and a maximum cut off of 0.2 for any minor variants within the consensus. The final barcode and/or variant lookup tables were used as a reference for all downstream analyses of short-read Illumina sequencing of the barcodes only.

The final barcode-variant tables are available from GitHub at https://github.com/dms-vep/RSV_Long_F_DMS/blob/main/results/variants/codon_variants.csv.

For full details on the analysis, see these notebooks for the following:

- Analysing the PacBio CCSs: https://dms-vep.org/RSV_Long_F_DMS/notebooks/analyze_pacbio_ccs.html
- Building PacBio consensus sequences: https://dms-vep.org/RSV_Long_F_DMS/notebooks/build_pacbio_consensus.html
- Building the final barcode-variant table: https://dms-vep.org/RSV_Long_F_DMS/notebooks/build_codon_variants.html.

The final libraries contained 57,888 and 64,078 unique barcoded variants for libraries LibA and LibB, respectively, that covered 99% and 99.4% of all amino acid mutations (Extended Data Fig. 1c). More than half of the variants contain a single amino acid mutation, whereas the others have zero or many mutations (Extended Data Fig. 1d).

### Measuring effects of F mutations on cell entry

To measure effects of F mutations on cell entry, we generally followed the approach described in ref. ^30^ with the following modifications. We infected 293T-TIM1 cells with the pseudovirus libraries showing the RSV F-protein mutants alongside parallel infection of 293T-TIM1 cells with control pseudovirus showing VSV-G to make their cell entry independent of RSV F-protein function (Extended Data Fig. 2a). Next, 2 × 10^6^ 293T-TIM1 cells were plated in each well of six-well plates coated with poly-l-lysine. The next day, we infected cells with roughly 12 × 10^6^ TU total (3 × 10^6^ TU per well) of F pseudovirus library or roughly 3 × 10^7^ TU total (5 × 10^6^ TU per well) of VSV-G pseudovirus library. After addition of pseudovirus, cells infected with the F pseudovirus library were spun at 900*g* for 3 h at 30 °C. For some selections, 20 μg ml^−1^ diethylaminoethyl (DEAE)-dextran was added to the media at the time of infection as this increases pseudovirus titres. However, poor cell health was found to contribute to reduced recovery of infecting barcodes, so DEAE was not used for follow-up selections. At 12 h following infection, the non-integrated reverse-transcribed lentiviral genomes were recovered by miniprepping the cells. To prepare the amplicons for Illumina sequencing with dual indexing, PCR was performed as described in ref. ^64^. Samples were pooled in equal DNA amounts and run on a 1% agarose gel. The correct size band was excised, purified with AMPure XP beads, diluted to a concentration of 5 nM and sequenced on an Illumina NextSeq 2000 (with P3 reagent kit) or NovaSeq X Plus system.

We quantified the effects of mutations as the log_2_ cell entry of each mutant relative to the unmutated F, using global-epistasis models^33,34^ to jointly analyse the single and multi-mutant data. Briefly, Illumina sequencing reads were aligned to the barcode-variant table generated from the PacBio CCS described above. We next compared the frequency of barcodes between the VSV-G and *F*_mutant_ selections using the package dms_variants (https://github.com/jbloomlab/dms_variants) as previously described in ref. ^30^. Cell entry scores for each variant were calculated using log enrichment ratio: log_2_ [(*n*^*v*^_post_/*n*^wt^_post_)/(*n*^v^_pre_/*n*^wt^_pre_)], where *n*^*v*^_post_ is the count of variant *v* in the F-pseudotyped infection (post-selection condition), *n*^*v*^_pre_ is the count of variant *v* in the VSV-G-pseudotyped infection (pre-selection condition), and *n*^wt^_post_ and *n*^wt^_pre_ are the counts for wild-type variants. Positive cell entry scores indicate that a variant is better at entering the cells compared with the unmutated parental F, and negative scores indicate entry worse than the parental F. As expected, variants with only synonymous mutations had wild-type-like cell entry scores of zero, variants with stop codon mutations had highly negative scores and variants with amino acid mutations had scores ranging from wild-type-like to highly negative (Extended Data Fig. 2b). To calculate the mutation-level cell entry effects, a sigmoid global-epistasis function was fitted to variant entry scores after truncating the values at a lower bound of the median functional score of all variants with stop codons, using the multi-dms software package. For the mutation effects, values of zero mean no effect on cell entry, negative values mean impaired cell entry and positive values mean improved cell entry. To generate the final cell entry effect values for each mutation, we performed a total of three technical replicates for each library, for a total of six functional selections (three each from LibA and LibB). The effects for each mutation were then averaged from the six functional selections. To filter out low quality or noisy data, we required each mutation to occur with two unique barcodes and removed any mutation that had a high standard deviation between replicates. The measured cell entry effects were highly correlated both between replicates and libraries (Extended Data Fig. 2c,d). The final cell entry effect values reported in the figures correspond to the average effect across libraries and replicates.

### Measuring effects of F mutations on antibody neutralization

To measure effects of F mutations on antibody neutralization, we used a previously described method from ref. ^30^ with a few modifications detailed here. A total of 2 × 10^6^ 293T-TIM1 cells were plated in individual wells of poly-l-lysine coated six-well plates. The next day, roughly 1.5–2 × 10^6^ TU of the F pseudovirus library were incubated with D10 media (no-antibody control) or antibody for 1 hour before adding to cells. For monoclonal antibodies, these incubations were performed in a total volume of 2 ml to reduce the possibility of ligand depletion given that these antibodies have very high potency. Antibody concentrations were selected that generally corresponded to a range at which 50% of variants were neutralized, up to 99.5%. During DNA template extraction, we spiked in DNA plasmid containing eight known barcodes that would correspond to roughly 1% of the reads in the no-antibody control. This DNA plasmid spike-in allowed us to estimate the amount of neutralization each antibody condition had relative to the no-antibody control, as previously described in refs. ^30,64^ (Extended Data Fig. 4a).

Following Illumina sequencing of the barcodes, the data were analysed as described previously. Briefly, the fraction infectivity retained at each antibody concentration was calculated from the barcode counts of the DNA standard. Then, the polyclonal (https://jbloomlab.github.io/polyclonal/)^66^ software was used to fit neutralization curves and estimates of mutation effects on neutralization. We filtered the effects of mutations on antibody neutralization to retain only mutations with at least two unique barcodes, excluded mutations with very low cell entry scores and excluded mutations that had high standard deviations in their effect on neutralization across replicates. The reported effects of mutations on antibody neutralization are the average across all replicates (always at least two different experimental selections with each of the two independent libraries, LibA and LibB). For example notebooks showing these analyses, see https://dms-vep.org/RSV_Long_F_DMS/notebooks/fit_escape_antibody_escape_RSV-F-LibA-250402-Nir.html for analysis of an individual selection experiment and https://dms-vep.org/RSV_Long_F_DMS/notebooks/avg_escape_antibody_escape_Nirsevimab-IgG.html for average effects across experiments for an antibody.

### Validation of cell entry effects using individual pseudoviruses

To validate the effects of mutations on cell entry, we generated a set of plasmids expressing RSV F within a lentiviral backbone that each contained different single F mutations. The parental RSV F amino acid sequence is identical to the unmutated RSV F sequence used in the lentiviral vector described above for deep mutational scanning. We selected mutations spanning a range of entry effects, which included N67I, S215V, S215P, S398L, E87N and D486N. We transfected them into 293T cells, along with helper plasmids (AddGene: HDM-tat1b product ID 204154, pRC-CMV-Rev1b product ID 20413, HDM-Hgpm2 product ID 204152)^30^ and RSV G in an expression plasmid (AddGene: HDM_RSV_Long_G_31AACTdel Product ID237350). After 48 h, supernatants were filtered through 0.45-μM filters to remove cell debris. The pseudoviruses were titrated on 293T-TIM1 cells using flow cytometry to measure TU per millilitre as previously described in ref. ^31^. Average titres for two replicates of each pseudovirus were then compared to the average unmutated RSV F titre.

### Cell entry effects of amino acid differences in natural sequences

An alignment of RSV F-protein sequences for subtype A and B were generated on 27 October 2025 from the RSV Nextstrain workflow^45^. We identified amino acid differences relative to the laboratory-adapted subtype A Long strain used in the deep mutational scanning (for more details see https://github.com/dms-vep/RSV_Long_F_DMS/tree/main/notebooks/sequence_variation). This was used to compare the distribution of cell entry effects for mutations observed in natural sequences to the distribution of cell entry effects for all mutations measured by deep mutational scanning and calculate the percentage of natural sequences with each mutation as shown in Extended Data Fig. 3.

### Measuring effects of mutations on antibody neutralization

We chose previously known and newly identified resistance mutations that had a range of effects on antibody neutralization for nirsevimab and clesrovimab. These mutants were chosen to evaluate the impact of mutations in subtype A and B backgrounds on neutralization by antibody IgG and Fab and to validate the effects of mutations measured by deep mutational scanning on antibody neutralization. The single amino acid mutations were made in a subtype A background (Long strain, also used for deep mutational scanning) and a subtype B background (B1 strain) in an expression vector. The plasmids were generated by Twist Biosciences. Pseudoviruses were produced and neutralization was measured as previously described in ref. ^31^. All F constructs included the full cytoplasmic tail and were paired with RSV G in an expression plasmid (AddGene: HDM_RSV_Long_G_31AACTdel Product ID 237350) for these transfections. All plasmid maps can be found through GitHub at https://github.com/dms-vep/RSV_Long_F_DMS/tree/main/supplemental_data/plasmids/. For all plotted neutralization curves, points represent the mean and standard error of at least two replicate measurements.

Mutations affecting nirsevimab neutralization made in subtype A included (N67T, K68N, K68Q, D73N, K201S, K201T, P205S, V207E, K209D, K209Q, Q210T, S211R, S215K) and subtype B (K68N, K68Q, D73N, N201S, N201T, P205S, Q209D, Q210T, S211R, S215K). V207E did not produce usable pseudovirus titres in the B1 background. These are shown in Fig. 3 and Extended Data Fig. 4.

Mutations T67N and Q209K were also made in subtype B, which are mutations to the amino acid residue found in the subtype A Long strain. Data for these mutants along with the corresponding mutations in subtype A (N67T and K209Q) are shown in Extended Data Fig. 4. Subtype A K68Q and K201S and subtype B K68Q and N201S are shown in Figs. 1 and 3 and Extended Data Fig. 4.

The Long and B1 wild-type neutralization curves shown in Fig. 3d,f are representative: all replicates can be found through GitHub at https://github.com/dms-vep/RSV_Long_F_DMS/tree/main/non-pipeline_analyses/validations. The correlation plots in Figs. 3 and 4 show fold-change IC_50_ from wild type using a matched wild type from the specific experimental date for the mutations and the wild-type point plotted in the correlation plots is a geometric mean IC_50_ of all wild-type replicates.

Mutations affecting clesrovimab made in subtype A and B included R429M, R429S, S443P and G446D and are shown in Fig. 4 and Extended Data Fig. 6. Pseudoviruses expressing F called ‘A2020’ and ‘B2024’ in Extended Data Fig. 6a are F sequences from natural strains that are broadly representative of recent subtype A and B strains^31^, and have the GenBank accession numbers PP495954.1 and PP660445.1, respectively.

### Interactive Nextstrain trees with antibody-escape scores

We integrated computer code into the Nextstrain^45^ RSV view to enable scoring of natural RSV sequences for antibody resistance and visualization of the results on phylogenetic trees. A repository with the computer code implementing this scoring is available from GitHub at http://github.com/nextstrain/rsv. Briefly, the pre-existing Nextstrain view downloaded all RSV sequences from Pathoplexus^46^ (at present, more than 60,000), and built separate subtype A and B phylogenetic trees subsampled to roughly 3,000 sequences designed to be representative across time and countries. We added computer code that assigns each of these sequences an ‘escape score’ calculated from the deep mutational scanning data as either the sum of the effect of all of its constituent mutations or the max effect of any of its mutations on neutralization by the Fab or IgG form of nirsevimab or clesrovimab. The interactive phylogenetic trees can then be coloured by these escape scores using the dropdown ‘Color By’ option on the left toolbar (for example, https://nextstrain.org/rsv/b/F-antibody-escape/6y?c=Nirsevimab-Fab_total_escape); there is also an option to label sequences by their top escape mutation. We integrated this scoring into the Nextstrain RSV builds for the F sequences and the full genome; note that there are also builds that emphasize different timeframes: all-time, the past 6 years and the past 3 years. The builds are updated to include the latest available sequences, so going to views linked above will show the latest sequence data.

We also created new builds called ‘F-antibody-escape’ in the Nextstrain RSV views that shows trees subsampled to include all sequences with high nirsevimab or clesrovimab escape scores. Unlike the ‘F’ builds, these ‘F-antibody-escape’ builds over-represent the frequency of resistant strains. The ‘F-antibody-escape’ trees do not provide an accurate view of the prevalence of resistance mutations; however, when you want to identify all of the top resistant strains (which otherwise could be dropped during subsampling) then these builds should be preferred. Which build is shown can be selected using the ‘change dataset’ option on the left toolbar.

### Validation of strains with predicted neutralization resistance

A subset of natural RSV F sequences with predicted resistance to nirsevimab or clesrovimab neutralization identified using the ‘Interactive Nextstrain phylogenetic trees with antibody-escape scores’ were codon optimized and subsequently cloned into an expression vector by Twist Biosciences. F sequences with predicted resistance to nirsevimab Fab or IgG included (PP_002XVQT, PP_002KSRJ, PP_002QMLP, PP_002WHEU, PP_002WWH8, PP_002SUFP, PP_001QYN9, PP_001Y2UB, PP_003W55P). Sequences with predicted resistance to clesrovimab Fab or IgG included (PP_002W1BG, PP_001WGC0, PP_001Y62S, PP_001ZQ7W). PP_001W26S and PP_002UDSB correspond to subtype A and B controls also referred to in our previous study as A2020 and B2024, respectively^31^. A complete list of these key sequences are available in Pathoplexus under SeqSet PP_SS_628.1 (ref. ^67^) and all sequences shown in the trees in Fig. 5 and Extended Data Fig. 8 are available in Pathoplexus under SeqSet PP_SS_661.1 (ref. ^68^) for subtype A and SeqSet PP_SS_662.1 (ref. ^69^) for subtype B. All plasmid maps can be found through GitHub (https://github.com/dms-vep/RSV_Long_F_DMS/tree/main/supplemental_data/plasmids/strain%20validations). These were used to generate RSV pseudoviruses expressing the F from the natural sequences paired with RSV G expression plasmid (AddGene: HDM_RSV_Long_G_31AACTdel Product ID237350) as previously described in ref. ^31^. Producing RSV pseudoviruses with F from natural sequences paired with all the same G ensures we do not introduce any variability from G. These pseudoviruses were then used in neutralization assays with nirsevimab or clesrovimab IgG and Fab^31^. To ensure reliable neutralization curves, we established a cut off of 400,000 relative light units per well for the no-antibody, virus-only control. For strains with titres near the cut off, extra controls were included to ensure escape was specific to the monoclonal antibody and not attributable to experimental artefacts.

### Structural analysis

UCSF ChimaX^70^ was used for structural visualizations. All PDB accession IDs used are included in figure legends.

### Reporting summary

Further information on research design is available in the Nature Portfolio Reporting Summary linked to this article.

## Online content

Any methods, additional references, Nature Portfolio reporting summaries, source data, extended data, supplementary information, acknowledgements, peer review information; details of author contributions and competing interests; and statements of data and code availability are available at https://doi.org/10.1038/s41586-026-11030-4.

## Supplementary information

Supplementary Table 1 Effects of mutations on cell entry and monoclonal antibody neutralization. This CSV file is also available through GitHub at https://github.com/dms-vep/RSV_Long_F_DMS/blob/main/results/summaries/all_antibodies.csv.

Reporting Summary

Supplementary Appendix Biophysical model of how bivalent binding shapes affect viral mutations on IgG versus Fab neutralization.

Peer Review File

## Data Availability

All data and interactive figures are publicly available. The homepage for exploring interactive visualizations of deep mutational scanning data is available from DMS-VEP (https://dms-vep.org/RSV_Long_F_DMS/). The repository comprising processed data for all sequencing counts and inferred mutation effects is available at GitHub (https://github.com/dms-vep/RSV_Long_F_DMS) and archived on Zenodo (https://doi.org/10.5281/zenodo.21049419)^71^. Final measured effects of mutations on cell entry and antibody neutralization after data quality control are shown in Supplementary Table 1 and are available at GitHub (https://github.com/dms-vep/RSV_Long_F_DMS/blob/main/results/summaries/all_antibodies.csv). Viral genome sequences and associated metadata are available from Pathoplexus (https://doi.org/10.62599/PP_SS_628.1, https://doi.org/10.62599/PP_SS_661.1 and https://doi.org/10.62599/PP_SS_662.1)^67–69^. Previously published structures used for data visualization are PDB 5UDC (ref. ^13^), PDB 6OUS (ref. ^14^) and PDB 9LLY (ref. ^52^).

## Code availability

A repository with all code used to analyse and visualize deep mutational scanning data is available at GitHub (https://github.com/dms-vep/RSV_Long_F_DMS) and archived on Zenodo (https://doi.org/10.5281/zenodo.21049419)^71^.
