# Supplementary material for: Mutational constraints on RSV F and its neutralization by antibodies
Source: Nature. 2026 Sep 16;658(8137):998–1007. doi: 10.1038/s41586-026-11030-4 (PMC13587686; doi:10.1038/s41586-026-11030-4)
Supplement: Supplementary file 1 [file 41586_2026_11030_MOESM2_ESM.pdf]

nature portfolio

Corresponding author(s): Bloom, Jesse D

Last updated by author(s): Aug 6, 2026

Reporting Summary

Nature Portfolio wishes to improve the reproducibility of the work that we publish. This form provides structure for consistency and transparency in reporting. For further information on Nature Portfolio policies, see our Editorial Policies and the Editorial Policy Checklist.

Statistics

For all statistical analyses, confirm that the following items are present in the figure legend, table legend, main text, or Methods section.

n/a	Confirmed	
☐	☒	The exact sample size (n) for each experimental group/condition, given as a discrete number and unit of measurement
☐	☒	A statement on whether measurements were taken from distinct samples or whether the same sample was measured repeatedly
☒	☐	The statistical test(s) used AND whether they are one- or two-sided<br>*Only common tests should be described solely by name; describe more complex techniques in the Methods section.*
☐	☒	A description of all covariates tested
☒	☐	A description of any assumptions or corrections, such as tests of normality and adjustment for multiple comparisons
☐	☒	A full description of the statistical parameters including central tendency (e.g. means) or other basic estimates (e.g. regression coefficient) AND variation (e.g. standard deviation) or associated estimates of uncertainty (e.g. confidence intervals)
☒	☐	For null hypothesis testing, the test statistic (e.g. F, t, r) with confidence intervals, effect sizes, degrees of freedom and P value noted<br>*Give P values as exact values whenever suitable.*
☒	☐	For Bayesian analysis, information on the choice of priors and Markov chain Monte Carlo settings
☒	☐	For hierarchical and complex designs, identification of the appropriate level for tests and full reporting of outcomes
☐	☒	Estimates of effect sizes (e.g. Cohen's d, Pearson's r), indicating how they were calculated

Our web collection on statistics for biologists contains articles on many of the points above.

Software and code

Policy information about availability of computer code

Data collection

The analyses of the deep mutational scanning were performed with the dms-vep-pipeline-3 version 3.31.0, which is publicly available on GitHub at https://github.com/dms-vep/dms-vep-pipeline-3. The GitHub repository that runs this pipeline and contains all the sequencing data and configuration for the analysis is publicly available on GitHub at https://github.com/dms-vep/RSV_Long_F_DMS and archived on Zenodo at DOI 10.5281/zenodo.21049419.

The phylogenetic analyses were performed using Nextstrain, with the full code available at https://github.com/nextstrain/rsv. The Nextstrain trees at the time of manuscript acceptance used the latest command-line release (11.0); however the trees are updated continuously to include newer RSV sequences and to use newer software versions.

Sequences were codon optimized using the GenSmart codon optimization tool offered by GenScript available at https://www.genscript.com/tools/gensmart-codon-optimization. This online tool provided by GenScript does not list a version number.

Data analysis

The data analysis was performed using the code in https://github.com/dms-vep/RSV_Long_F_DMS; and the environment.yml file in this repository (https://github.com/dms-vep/dms-vep-pipeline-3/blob/0d9c4933d7c29765833d31976f83c1ddc44793b9/environment.yml) provides the exact version of all software used. The analyses of the deep mutational scanning were performed with the dms-vep-pipeline-3 version 3.31.0, which is publicly available on GitHub at https://github.com/dms-vep/dms-vep-pipeline-3.

A GitHub repository with all code used to analyze and visualize deep mutational scanning data, as well as processed data giving all sequencing counts and inferred mutation effects is available at https://github.com/dms-vep/RSV_Long_F_DMS and archived on Zenodo at DOI 10.5281/zenodo.21049419.

For manuscripts utilizing custom algorithms or software that are central to the research but not yet described in published literature, software must be made available to editors and reviewers. We strongly encourage code deposition in a community repository (e.g. GitHub). See the Nature Portfolio guidelines for submitting code & software for further information.

Data

Policy information about availability of data

All manuscripts must include a data availability statement. This statement should provide the following information, where applicable:

- Accession codes, unique identifiers, or web links for publicly available datasets
- A description of any restrictions on data availability
- For clinical datasets or third party data, please ensure that the statement adheres to our policy

All data and interactive figures are publicly available:

Homepage for exploring interactive visualizations of deep mutational scanning data: https://dms-vep.org/RSV_Long_F_DMS/
GitHub repository of all code used to analyze and visualize deep mutational scanning data, as well as processed data giving all sequencing counts and inferred mutation effects: https://github.com/dms-vep/RSV_Long_F_DMS
Final measured effects of mutations on cell entry and antibody neutralization after data QC: Supplementary File 1 and https://github.com/dms-vep/RSV_Long_F_DMS/blob/main/results/summaries/all_antibodies.csv
The underlying data for the neutralization curves is available at https://github.com/dms-vep/RSV_Long_F_DMS/blob/main/non-pipeline_analyses/validations/01_data/combined_frac_infect.csv
Viral genome sequences and associated metadata were obtained from Pathoplexus and can be found at https://doi.org/10.62599/PP_SS_628.1, https://doi.org/10.62599/PP_SS_661.1, and https://doi.org/10.62599/PP_SS_662.1
Previously published structures used for data visualization: PDB 5UDC13, PDB 6OUS14, PDB 9LLY52

Research involving human participants, their data, or biological material

Policy information about studies with human participants or human data. See also policy information about sex, gender (identity/presentation), and sexual orientation and race, ethnicity and racism.

Reporting on sex and gender	Not applicable
Reporting on race, ethnicity, or other socially relevant groupings	Not applicable
Population characteristics	Not applicable
Recruitment	Not applicable
Ethics oversight	Not applicable

Note that full information on the approval of the study protocol must also be provided in the manuscript.

Field-specific reporting

Please select the one below that is the best fit for your research. If you are not sure, read the appropriate sections before making your selection.

☒ Life sciences ☐ Behavioural & social sciences ☐ Ecological, evolutionary & environmental sciences

For a reference copy of the document with all sections, see nature.com/documents/nr-reporting-summary-flat.pdf

Life sciences study design

All studies must disclose on these points even when the disclosure is negative.

Sample size

We created two independent deep mutational scanning libraries and performed replicate measurements with each; an extended data figure contains a plot with replicate-to-replicate correlations and the interactive online plots allow per-replicate analysis of each measurement by mousing over the heat maps. This was the scale of experiment that was feasible to perform and is sufficient to allow quantification of replicate-to-replicate and library-to-library variation.

Data exclusions	We only report the effects of mutations measured in both libraries and seen in at least two independent barcodes per library. This allows us to exclude values that may be less accurate due to lower numbers of independent measurements.
Replication	As described in the paper, key mutations identified in the deep mutational scanning were validated by generating them independently and performing pseudovirus neutralization assays. As noted above, we created two independent deep mutational scanning libraries and performed replicate measurements with each. All replicates were highly correlated.
Randomization	Randomization was not applicable to this study because it did not involve allocation of samples to experimental groups. Experiments were performed using RSV F deep mutational scanning libraries and in vitro assays.
Blinding	The sequencing and analysis is done on the basis of barcodes, and only after all of the processing are the measured sequencing effects associated with specific mutants. There were no experimental groups or group allocation for blinding during data collection.

Reporting for specific materials, systems and methods

We require information from authors about some types of materials, experimental systems and methods used in many studies. Here, indicate whether each material, system or method listed is relevant to your study. If you are not sure if a list item applies to your research, read the appropriate section before selecting a response.

Materials & experimental systems

n/a	Involved in the study
☐	☒ Antibodies
☐	☒ Eukaryotic cell lines
☒	☐ Palaeontology and archaeology
☒	☐ Animals and other organisms
☒	☐ Clinical data
☒	☐ Dual use research of concern
☒	☐ Plants

Methods

n/a	Involved in the study
☒	☐ ChIP-seq
☒	☐ Flow cytometry
☒	☐ MRI-based neuroimaging

Antibodies

Antibodies used	The IgG and Fab forms of nirsevimab, clesrovimab, suptavumab, palivizumab, RSM01, 1A2 and 1B6 were produced commercially using the VH and VL sequences.
Validation	The antibodies were produced commercially from Genscript using the VH / VL sequences, ensuring the correct antibodies. They were validated for the expected neutralization of RSV pseudovirus.

Eukaryotic cell lines

Policy information about cell lines and Sex and Gender in Research

Cell line source(s)	293T cells were obtained from ATCC, and modified in the Bloom lab to express TIM1 as described in prior papers linked in the Methods. LentiX cells were purchased commercially from Takara.
Authentication	We confirmed the cells supported pseudovirus production and infection which were the only uses in this paper.
Mycoplasma contamination	The parental 293T cell line was validated to be negative for mycoplasma via testing at the Fred Hutch Genomics Core. The 293T-TIM1 cells were derived from the parental 293T cells. The LentiX cells were newly purchased from Takara and were not tested for mycoplasma contamination by us.
Commonly misidentified lines (See ICLAC register)	No commonly misidentified cell lines were used in this study.

Plants

Seed stocks

Report on the source of all seed stocks or other plant material used. If applicable, state the seed stock centre and catalogue number. If plant specimens were collected from the field, describe the collection location, date and sampling procedures.

Novel plant genotypes

Describe the methods by which all novel plant genotypes were produced. This includes those generated by transgenic approaches, gene editing, chemical/radiation-based mutagenesis and hybridization. For transgenic lines, describe the transformation method, the number of independent lines analyzed and the generation upon which experiments were performed. For gene-edited lines, describe the editor used, the endogenous sequence targeted for editing, the targeting guide RNA sequence (if applicable) and how the editor was applied.

Authentication

Describe any authentication procedures for each seed stock used or novel genotype generated. Describe any experiments used to assess the effect of a mutation and, where applicable, how potential secondary effects (e.g. second site T-DNA insertions, mosiacism, off-target gene editing) were examined.
