# Supplementary material for: Mutational constraints on RSV F and its neutralization by antibodies
Source: Nature. 2026 Sep 16;658(8137):998–1007. doi: 10.1038/s41586-026-11030-4 (PMC13587686; doi:10.1038/s41586-026-11030-4)
Supplement: Supplementary file 2 [file 41586_2026_11030_MOESM3_ESM.pdf]

# Supplementary Appendix: biophysical model of how bivalent binding shapes impact of viral mutations on IgG versus Fab neutralization

Here we describe a biophysical model of how the effect of a mutation to a viral antigen on neutralization by an antibody depends on the antibody's affinity for the antigen. The two key effects modeled here are the bivalent binding of the IgG (but not Fab) and the ligand depletion (titration) that can put a floor on the lowest measurable neutralization value. The model is illustrated conceptually in Figure 1a, and is used to generate the simulated data in Figure 1b that is compared to the actual experimental data in Figure 1c.

See https://github.com/jbloomlab/IgG-vs-Fab-neutralization for the computer code implementing the model, and https://jbloomlab.github.io/IgG-vs-Fab-neutralization/notebook.html for an interactive notebook describing the model.

### Fab Binding

Let the binding affinity of the Fab have dissociation constant $K_D$. There are two states, unbound ($U$) and bound ($B$). The Boltzmann weights for these two states as a function of the molar concentration $c$ of Fab are $w_U(c) = 1$ and $w_B(c) = \frac{c}{K_D}$. So the probability of the unbound state as a function of $c$ is $p_U^{Fab}(c) = \frac{w_U(c)}{w_U(c) + w_B(c)} = \frac{1}{1 + c/K_D}$ . This two-state partition-function formulation for monovalent binding follows classic multivalency treatments[1,2].

### IgG binding

For the IgG, we assume each arm of the bivalent IgG has the same monovalent affinity for the viral antigen as the Fab described above. However, once the first arm binds, the effective concentration $c_{eff}$ of the second arm is set by the density/spacing of viral antigens and the reach of the antibody. Here we do not consider those factors (antigen density, antibody reach, etc) separately, but just collapse them into the single effective concentration of the second arm after the first has bound, $c_{eff}$. The definition of an effective local concentration for the second arm, set by geometry/linker statistics rather than bulk concentration mirrors prior work[3–5]. In realistic scenarios, if bivalent binding is possible then $c_{eff} \gg c$ since the effective concentration of the second Fab arm in proximity to the viral antigen after the first arm has bound is much higher than the overall IgG concentration (note that concentrations should be in *molar* units rather than ng/ml in order to allow the same concentration scale to be used for Fab and IgG, since they have different molecular weights).

There are now three possible states, unbound ($U$), a single Fab arm bound ($B_1$), and both Fab arms bound ($B_2$). The Boltzmann weights for these three states as a function of the molecular concentration of IgG are $w_U(c) = 1$, $w_{B_1}(c) = \frac{c}{K_D}$, and $w_{B_2}(c) = \frac{c}{K_D} \times \frac{c_{eff}}{K_D}$. Further, note that state $B_1$ has a multiplicity of two since either of the Fab arms can bind[2]. So the probability of the unbound state as a function of $c$ is (accounting for the multiplicity of two for $B_1$) is

$p_U^{IgG}(c) = \frac{w_U(c)}{w_U(c) + 2w_{B_1}(c) + w_{B_2}(c)} = \frac{1}{1 + \frac{c}{K_D}\left(2 + \frac{c_{eff}}{K_D}\right)}$. Note that the effective dissociation constant of the IgG is $K_D^{IgG} = K_D / \left(2 + \frac{c_{eff}}{K_D}\right)$.

## Assumption: binding affinity equals neutralization potency

In what follows, we assume that binding affinity equals neutralization potency, such that the neutralization IC50 of the Fab is equivalent to $K_D$. In other words, we equate fraction infectivity with fraction of viral epitopes ($p_u$); as described immediately below, deviations can occur under multi-hit models. Specifically, how fraction bound (binding affinity) relates to neutralization potency depends on how many antibodies must bind to a virion in order to neutralize it. The quantitative relationship between binding affinity and neutralization potency as well as its impact on the neutralization curve slope can be modeled under assumptions about the number of spikes per virions and the number that must be bound to neutralize[6–10]. Here we are going to *ignore* this issue for now, and just conflate neutralization IC50 with Fab $K_D$. This is a quantitative simplification, but should not affect the main qualitative trends—and has the advantage of not requiring us to estimate difficult to determine quantities such as how many spikes there are per virion and what fraction of them must be bound for neutralization. Note that in the limiting case where this is just one spike per virion and binding one site on it is sufficient for neutralization, then ignoring this issue has no effect.

## Estimating $c_{eff}$ from the IgG versus Fab IC50

We can estimate $c_{eff}$ (the parameter that describes the extent of avidity from bivalent binding) from the IC50 (or midpoint on the neutralization curve) of the IgG versus Fab. In particular, let $m_{Fab}$ and $m_{IgG}$ be the molar concentrations at the midpoint (IC50) of the Fab versus IgG neutralization curves, respectively. Then $K_D = m_{Fab}$ and $1/2 = \frac{1}{1 + \frac{m_{IgG}}{m_{Fab}}\left(2 + \frac{c_{eff}}{m_{Fab}}\right)}$. Solving for $c_{eff}$ yields $c_{eff} = m_{Fab}\left(\frac{m_{Fab}}{m_{IgG}} - 2\right)$.

## Impact of mutation(s)

Consider one or more mutations that cause a fold change in Fab IC50 of $f_{mut}$. For instance, a mutation that increases the Fab IC50 by 20-fold would have $f_{mut} = 20$. Note that mutations that cause a fixed change in the free energy of binding are expected to cause the same fold change in Fab IC50 in any genetic background. In the presence of these mutation(s), the fraction of Fab not neutralized becomes $p_U^{Fab}(c) = \frac{1}{1 + \frac{c}{f_{mut} \times K_D}}$ and the fraction of IgG not neutralized becomes $p_U^{IgG}(c) = \frac{1}{1 + \frac{c}{f_{mut} \times K_D}\left(2 + \frac{c_{eff}}{f_{mut} \times K_D}\right)}$.

## Complication: accounting for ligand depletion

Everything above is written in terms of the antibody (Fab or IgG) concentration $c$. In fact, $c$ in these equations refers to the *free* concentration of antibody. But when the concentration of viral antigen is comparable to or exceeds the dissociation constant of the antibody ($K_D$ for the Fab and $K_D^{IgG}$ for the IgG), there can be "ligand depletion," which is the term we use here for when a large fraction of the antibody is bound to the viral antigen. When this occurs, much of the antibody gets "soaked up" by binding to viral antigen, and so the actual concentration of free antibody in the neutralization assay represented by $c$ in

the above equations is appreciably less than the total concentration added to the reaction. This phenomenon is nicely described in detail by Jarmoskaite et al (2020)[11], who refer to it as the "titration regime."

To quantitatively deal with this, we need to define some additional variables. Let $c_{total}$ be the total antibody concentration added to the assay volume, let $c_{free}$ be the concentration of antibody that is not bound to viral antigen (free), and let $c_{bound}$ be the concentration of antibody that is bound to viral antigen. Also, let $v_{total}$ be the total concentration of viral antigen (quantified in terms of number of the relevant epitope per unit volume), let $v_{free}$ be the concentration of free viral antigen (not bound by antibody), and note that concentration of bound viral antigen is $c_{bound}$. We have the following equations: $c_{total} = c_{free} + c_{bound}$ (mass balance for the antibody), $v_{total} = v_{free} + c_{bound}$ (mass balance for the viral antigen), and $c_{bound} \times K_D^{effective} = v_{free} \times c_{free}$ (there is an equilibrium with respect to the free antibody and antigen concentration). Note that for the Fab, $K_D^{effective} = K_D$ and for the IgG, $K_D^{effective} = K_D^{IgG}$.

Solving this system of three equations yields[11]

$$c_{bound} = \left(c_{total} + v_{total} + K_D^{effective} - \sqrt{\left(c_{total} + v_{total} + K_D^{effective}\right)^2 - 4c_{total} \times v_{total}}\right)/2 \text{ and so}$$

$$c_{free} = c_{total} - c_{bound} = \left(c_{total} - v_{total} - K_D^{effective} + \sqrt{\left(c_{total} + v_{total} + K_D^{effective}\right)^2 - 4c_{total} \times v_{total}}\right)/2.$$

Note that in the limit where the viral antigen concentration is limiting and so we are outside the ligand depletion regime (eg, $v_{total} \ll K_D^{effective}$), then we just have $c_{free} \approx c_{total}$. However, when the viral antigen concentration is comparable to or greater than $K_D^{effective}$, then it is important to substitute $c_{free}$ for $c$ in all of the equations above.

We can also calculate the actual observed IC50s accounting for ligand depletion. The IC50 (under our assumption above that this is when half of viral epitopes are bound) occurs when $1/2 = c_{bound} / v_{total}$. It turns out that this can be neatly solved to determine the actual observed IC50 (accounting for ligand depletion) is $c_{IC50,observed} = K_D^{effective} + v_{total}/2$, whereas the ideal IC50 if there is no ligand depletion (when $v_{total} \ll K_D^{effective}$), is just $c_{IC50,ideal} = K_D^{effective}$. The floor on the measurable IC50 (eg, the lowest value that can be measured) is therefore $v_{total}/2$.

## Realistic parameter estimates

We ballpark (these are rough estimates) realistic parameter values for nirsevimab and RSV F from our experiments (Figure 1c); the model-simulated data in Figure 1b use these parameters with the subtype A $K_D$ estimates for the high-affinity panel and the subtype B $K_D$ estimates for the low-affinity panel, and all other parameters shared between the panels as they are not expected to be subtype-specific. For the Fab IC50, which is represented by $m_{Fab}$ or $K_D$ in the above equations:

- subtype A (Long) strain: $K_D \sim 0.01$ nM
- subtype B (B1) strain: $K_D \sim 1$ nM

We also need to estimate $c_{eff}$; however, doing that is complicated by the fact that at least for subtype A, the IgG neutralization assays are likely in the ligand depletion range (what Jarmoskaite et al[11] call the

“titration regime”) where the IgG IC50 ($m_{IgG}$) cannot be measured accurately since the concentration of the viral antigen protein likely is comparable or greater to the true IgG IC50. So we will use neutralization measurements against the subtype B (B1) strain as any IgG ligand depletion should be less here due to the lower potency, and we assume that the actual avidity (potential for bivalent binding) captured in $c_{eff}$ should not be strain dependent. Against this strain, we have $m_{IgG}$ ~ 0.01 nM and $m_{Fab}$ ~ 1 nM. So this gives $c_{eff}$ ~ 100 nM. However, there is probably still ligand depletion in the measurement of $m_{IgG}$ since the subtype B IgG neutralization is just as good as subtype A, so we will add another factor of 10 and use $c_{eff}$ ~ 1000 nM (note this remains a very rough estimate and real value could be higher).

The hardest parameter to estimate is the number of viral epitopes $v_{total}$ which determines if there is ligand depletion. Roughly, we might estimate $10^6$ infectious particles per ml, with $10^2$ non-infectious particles for each infectious one, $10^2$ epitopes per particle. In that case, we have $10^{13}$ epitopes / liter, which gives $v_{total}$ ~ 0.01 nM. Note that this estimate, which was used in Figure 1b, is very much a rough estimate and could vary by an order of magnitude or more in either direction.

### Interactive visualization of IgG and Fab neutralization and how it is affected by mutations

Go to https://jbloomlab.github.io/IgG-vs-Fab-neutralization/chart.html for an interactive chart initialized with the ballpark-realistic parameter values above for the subtype A strain of how nirsevimab neutralization differs between the Fab and IgG, and how this is impacted by mutations. In the interactive chart, you can use the sliders to adjust the Fab affinity ($K_D$), the avidity ($c_{eff}$), the effect of the viral mutation(s) ($f_{mut}$), and the viral epitope concentration ($v_{total}$). As you do so, you can see how the neutralization curves and IC50s change. Note for the IC50s, the interactive chart shows both the actual observed IC50 (accounting for ligand depletion) as well as what would be the ideal IC50 in the absence of any ligand depletion.

**References**


1. Hlavacek, W. S., Posner, R. G. & Perelson, A. S. Steric Effects on Multivalent Ligand-Receptor Binding: Exclusion of Ligand Sites by Bound Cell Surface Receptors. *Biophys. J.* **76**, 3031–3043 (1999).
2. Perelson, A. S. & DeLisi, C. Receptor clustering on a cell surface. I. theory of receptor cross-linking by ligands bearing two chemically identical functional groups. *Math. Biosci.* **48**, 71–110 (1980).
3. Einav, T., Yazdi, S., Coey, A., Bjorkman, P. J. & Phillips, R. Harnessing Avidity: Quantifying the Entropic and Energetic Effects of Linker Length and Rigidity for Multivalent Binding of Antibodies to HIV-1. *Cell Syst.* **9**, 466-474.e7 (2019).
4. Mammen, M., Choi, S.-K. & Whitesides, G. M. Polyvalent Interactions in Biological Systems: Implications for Design and Use of Multivalent Ligands and Inhibitors. *Angew. Chem. Int. Ed.* **37**, 2754–2794 (1998).
5. Fasting, C. *et al.* Multivalency as a Chemical Organization and Action Principle. *Angew. Chem. Int. Ed.* **51**, 10472–10498 (2012).
6. Magnus, C. Virus Neutralisation: New Insights from Kinetic Neutralisation Curves. *PLoS Comput. Biol.* **9**, e1002900 (2013).
7. Brandenberg, O. F. *et al.* Predicting HIV-1 transmission and antibody neutralization efficacy in vivo from stoichiometric parameters. *PLOS Pathog.* **13**, e1006313 (2017).
8. Pierson, T. C. *et al.* The Stoichiometry of Antibody-Mediated Neutralization and Enhancement of West Nile Virus Infection. *Cell Host Microbe* **1**, 135–145 (2007).
9. Klasse, P. J. Neutralization of Virus Infectivity by Antibodies: Old Problems in New Perspectives. *Adv. Biol.* **2014**, 1–24 (2014).
10. Yang, X., Kurteva, S., Lee, S. & Sodroski, J. Stoichiometry of Antibody Neutralization of Human Immunodeficiency Virus Type 1. *J. Virol.* **79**, 3500–3508 (2005).
11. Jarmoskaite, I., AlSadhan, I., Vaidyanathan, P. P. & Herschlag, D. How to measure and evaluate binding affinities. *eLife* **9**, e57264 (2020).
