# Supplementary material for: Mutational constraints on RSV F and its neutralization by antibodies
Source: Nature. 2026 Sep 16;658(8137):998–1007. doi: 10.1038/s41586-026-11030-4 (PMC13587686; doi:10.1038/s41586-026-11030-4)
Supplement: Supplementary file 3 [file 41586_2026_11030_MOESM4_ESM.pdf]

nature portfolio

Peer Review File

Mutational constraints on RSV F and its neutralization by antibodies

Corresponding Author: Professor Jesse Bloom

Any redactions in this file are there to maintain patient confidentiality, the confidentiality of unpublished data, or to remove third-party material.

This file contains all reviewer reports in order by version, followed by all author rebuttals in order by version.

Version 0:

Reviewer comments:

Referee #1

(Remarks to the Author)
The authors built a pseudovirus-based DMS system for the RSV F protein and present a comprehensive DMS study to evaluate how mutations in the RSV F protein affect viral cell entry and neutralization by several key clinical mAbs, such as nirsevimab and clesrovimab. The study also proposes a biophysical model to explain subtype-specific differences in escape mutation prevalence based on the buffering effect of bivalent IgG binding. Furthermore, the authors integrate their DMS escape scores into the Nextstrain platform to facilitate real-time genomic surveillance of circulating RSV strains.
Overall, this study is technically sound, and the DMS results provide a critically detailed analysis that could explain or forecast viral escape from previous or existing therapeutic neutralizing antibodies. However, this study currently does not provide a substantially improved understanding of RSV evolution, nor does it give critical and direct insights into the development of vaccines or therapeutics against RSV, especially for neonates, at least in its current form. Below are my major concerns and suggestions:

Major Points
1. The biophysical model presented in Figure 1, which describes the buffering effect of bivalent IgG binding versus monovalent Fab binding, is based on well-established principles and lacks substantial conceptual novelty. While the authors provide extensive experimental comparisons between Fab and IgG affinities throughout the manuscript, these data feel somewhat disconnected from the core biological implications of the study (RSV antibody escape). I suggest moving this model and the related text to the supplemental materials. Otherwise, the authors are expected to clearly justify and provide direct evidence in the main text as to why Fab escape data are biologically necessary for understanding real-world, antibody-mediated immune pressure and viral evolution.
2. The DMS platform developed by the authors is robust and technically impressive. However, the study evaluates only a very limited number of specific therapeutic mAbs or candidates (e.g., nirsevimab, clesrovimab, suptavumab, palivizumab, RSM01). Consequently, the manuscript reads more like a high-quality product-testing report for existing drugs rather than a systematic exploration of RSV F protein mutational rules and broader antigenic evolution. While this is highly useful for monitoring resistance to current clinical therapies, it does not yield the comprehensive antigenic mapping required to directly inform the design of new vaccines or the discovery of novel therapeutic antibodies.
As a result, the core scientific problem addressed—mapping escape mutations for a few prophylactic mAbs—is somewhat narrow in scope for Nature. To substantially elevate the broad significance of this work, the authors need to establish a direct connection between RSV subtype evolution and broader, population-level immune selection pressures. I strongly recommend performing escape assays using human polyclonal sera (e.g., convalescent sera from natural infections or post-vaccination sera). Integrating polyclonal sera data would allow the authors to systematically link the evolutionary trajectories of RSV subtypes A and B to actual population-level immune pressures, thereby providing concrete and direct implications for future vaccine and antibody development.
3. Compared to proteins like the SARS-CoV-2 Spike, a notably larger number of sites on the RSV F protein severely impact cell entry. The authors should explain the underlying mechanism for this high functional constraint. For example, could the authors analyze their relationship to the structural features of the F protein? Additionally, in the validation assays, some individual mutations showed a negligible impact on cell entry, whereas they received notably low scores in the DMS dataset (Fig. 2B). The authors should address whether this discrepancy might lead to an overestimation of mutational impacts in the

DMS results.
4. The observation regarding mutations T101P and S99N highlights a known discrepancy between in vitro pseudovirus fitness in cell culture and authentic viral fitness in the human airway. The authors should expand upon this in the discussion to address how this limitation might affect the broader interpretation of the DMS cell entry scores for other uncharacterized mutations. Additional validation experiments or analyses may also be necessary to confirm the reliability of the cell entry DMS scores in reflecting true viral fitness.
5. The study discusses that the same amino acid mutations can escape mAbs differently depending on whether they are in a subtype A or subtype B background. However, the DMS library was constructed solely based on subtype A. Why did the authors not include a subtype B-based variant library in the DMS? Including this would facilitate a much more systematic analysis of the distinct antibody escape mechanisms between the two subtypes.
6. Although the analysis indicates that there is no immediate escape risk for nirsevimab and clesrovimab, could sequence analysis reveal other mutations under evolutionary pressure, and can these be mapped to specific antibody epitopes or functional DMS results? It would significantly strengthen the paper to explore whether historical RSV evolution demonstrates clear signatures of antibody-mediated selective pressure.

Minor Points
1. Please clearly define what the error bars represent in Fig. 2B, 3D, 3F, 4D, and 5C-D, as well as the black lines in Fig. 2E.
2. The numbers “2e6”, “12e6”, and “30e6” in the Methods section should be formatted using standard scientific notation.

(Remarks on code availability)
No major problem, since it was based on their previous well-recognized SARS-CoV-2 platform.

Referee #2

(Remarks to the Author)

This study addresses an important research question of how a comprehensive library of single RSV F mutations affects prophylactic mAbs. The manuscript presents a hypothesis-driven model that shows that the bivalency of IgG may retain neutralization activity against viral mutations in spite of a reduction in Fab affinity but depending on whether the initial Fab affinity was high or moderate. The model is combined with deep mutational scanning of the RSV F Long strain.The authors systematically measure the effects of amino acid substitutions on F phenotypes including cell entry and neutralization escape from Fab and IgG versions of the prophylactic mAbs. The work provides an unprecedented dataset that can be directly integrated into RSV surveillance pipelines, enabling rapid interpretation of newly observed mutations. However, this needs to be clarified in a short section explicitly outlining how surveillance might use these datasets. The data is presented in a clear and accessible manner, with figures that are easy to follow and that are well designed. Overall, this work adds valuable information to the field.

While the deep scanning techniques is not novel, application to the RSV field hasn’t been done before, and this is a valuable tool with public health utility. While the conceptual framework in figure 1 is similarly not unprecedented, I find the combination of the two powerful in thinking about viral escape, not just from RSV. This combining the mutational work with the mechanism behind the viral escape strengthens the paper.

The title is vague eg no mention of F. I also wonder if there is a way to incorporate the findings re fab/IgG somehow into the title.

The abstract summarizes the methods but does not give a summary of the main results, please add to the abstract.

The introduction is clear and concise
Recent and relevant references were used. However, in the introduction there was some over-citing, please carefully go through the references and select high quality focused sets. Do references 4 and 75 add any value? In addition there were some inconsistencies with the formatting of references 9, 32 and 88, please correct. If removing reference 4 please re-write the second half of 1st sentence. Defining the sites on pre-F that Nirsevimab and Clesrovimab recognise might improve readability. Please include evolutionary rates of F, HA and spike to add clarity. Please mention the year and location of the Suptavumab trial.

The method is very detailed and the approach used has been previously described by Dadonaite et al, in the context of deep mutational scanning for SARS-CoV-2. Therefore this could be written more concisely, focusing on modifications to protocol. A few sections require minor revisions such defining abbreviations upon first appearance and using terms in a consistent manner for example dox versus doxycycline.

The quality of the images is high including interactive figures. Some minor edits are suggested
Figure 1 in panel C- please clearly state Subtype A with high affinity and Subtype B with low affinity and in the legend provide clarity that A is the illustration of the biophysical model in which the hypothesis is…as the second part of the statement seems open-ended.

B orient the reader by defining the colours and shapes.

In general, the figure legends appear as interpretation of results instead of giving the reader direction on the figure.

Supplementary figure 1- define MOI. Supplementary figure 2- Specify correlation test used and add p-value (this should be done for all correlations reported).

For consistency and ease of reading can Figure 3 D and F be merged as done for Figure 4 D

It is not clear why Figure 3 E doesn't show the effects of mutations across Subtype B?

Supplementary Figure 7, Panel A, please re-arrange the mAb Fab and IgG plots so the order is consistent.

While our expertise is not in reviewing the code or formulas for analysis, the supplementary appendix describes in detail the modelling of how the biophysical properties of the antibodies affect neutralization activity to viral antigen mutations. Included here is an interactive plot in which one can adjust affinity and determine the impact on neutralization capacity of a Fab region versus IgG, this strengthens the models application as different conditions can be simulated as a proof of concept. We would defer to a reviewer more able to evaluate this section

(Remarks on code availability)

Referee #3

(Remarks to the Author)
I co-reviewed this manuscript with one of the reviewers who provided the listed reports.

(Remarks on code availability)

Referee #4

(Remarks to the Author)
Summary of the key results
This manuscript investigates how mutations in the RSV F protein influence neutralization by monoclonal antibodies currently used or under development for RSV prevention. The study is motivated by the increasing use of long-acting monoclonal antibodies (mAbs) which have substantially reduced RSV-related infant hospitalisations. The authors propose a biophysical explanation for observed differences in resistance mutation frequency between RSV-A and RSV-B strains, arguing that the buffering effect of bivalent IgG binding can partially compensate for reduced affinity caused by viral mutations. They have performed pseudovirus deep mutational scanning to evaluate the effects of nearly all possible amino-acid substitutions in the RSV F protein on viral entry and antibody neutralisation. This approach is used to quantify the neutralisation landscape for several anti-RSV mAbs, including nirsevimab and clesrovimab. Finally, the authors demonstrate how experimentally derived escape maps can be applied to surveillance datasets to identify circulating RSV strains carrying mutations associated with reduced antibody susceptibility. The work aims to support early detection of antibody resistance and guide development of future monoclonal antibodies with improved resilience to viral escape.

Originality and significance
The question addressed by the study is highly significant. As mAbs become a cornerstone of RSV prevention, particularly for infants, the emergence of viral escape mutations represents a major public health concern. The use of deep mutational scanning to generate a comprehensive functional map of RSV F protein mutations affecting antibody neutralisation represents an important technical and conceptual advance. While deep mutational scanning has previously been applied to other viral surface proteins, its application to RSV antibody escape remains relatively limited. Previous work has identified resistance mutations affecting monoclonal antibodies targeting RSV F, including escape mutations associated with palivizumab and suptavumab. However, these studies have generally relied on selection experiments or targeted mutagenesis rather than systematic mutational mapping. In that context, the comprehensive mutational landscape presented here appears novel and potentially valuable for surveillance and antibody design. The proposed biophysical model linking IgG bivalency and Fab affinity to subtype-specific resistance frequencies is conceptually interesting, although its broader significance will ultimately need stronger corroborative evidence. Overall, the manuscript addresses an important problem and provides data that could have substantial implications for RSV surveillance and therapeutic antibody development.

Data & methodology: validity of approach, quality of data, quality of presentation
The experimental approach combines biophysical modelling, pseudovirus assays, and deep mutational scanning, which together provide a powerful framework for mapping antibody escape mutations. The deep mutational scanning methodology appears appropriate, having been adapted from previous work. Generating a near-complete mutational dataset for the RSV

F protein is notable and provides a detailed view of the functional constraints governing antibody escape.
However, several methodological limitations should be noted, alongside those the authors already highlight:
1. Although pseudovirus assays are widely used for neutralisation studies, they may not fully capture the replication dynamics and structural constraints present in authentic RSV infection. Certain mutations identified as escape variants in pseudovirus systems may not be viable in replication-competent viruses.
2. The manuscript would be strengthened by experimental confirmation of key escape mutations in live RSV systems.
3. While the authors demonstrate how their mutational maps could be used to screen sequence databases, the practical implementation of this approach in real-time surveillance systems remains underdeveloped a this stage.
4. The RSV strain 'backbone' used for development of the pseudovirus was the Long strain from the 1950s, why not use a more contemporaneous strain?
5. It is already highlighted in the limitations section, but data on combinations of mutations would be extremely helpful for understanding this area.
6. As part of the cloning of the deep mutational scanning plasmid libraries, 268 mutations were 'missed' in the library produced by Twist. What is the explanation for this?
7. Why were the specific mutations that were chosen for the biophysical model (K68Q ad N201S), rather than others? How were thresholds for defining "resistance mutations" determined?
8. For clarity does "mutationally tolerant" mean it is less likely mutations will occur at the site or that if they do occur it is less likely to impact IgG/Fab binding?
9. The epitope for clesrovimab (same for palivizumab) is also on the postF form (compared with nirsevimab's which is only on the preF form), does that impact these results or the interpretation of the results?
10. It is unclear to me how the data described evidence the statement "…since the biophysical model described above suggests that Fab measurements are more generalizable across genetic backgrounds due to the absence of affinity-dependent buffering of bivalent IgG neutralization."
11. "Note that S211R slightly increases subtype B neutralization despite decreasing subtype A neutralization for both Fab and IgG; for this mutation there must be additional background-specific differences between subtypes". This is important for implications of overall generalisability of the results and should be highlighted clearly in the limitations section.
I note a couple of minor typos: page 35 "palizumab" and page 36 "with" is written twice.
Overall, the methodology appears appropriate and uses standard analytical approaches where feasible.

Conclusions: robustness, validity, reliability
The central conclusion, that systematic mapping of RSV F mutations can reveal escape pathways and support surveillance for antibody resistance, appears well supported by the experimental data. The finding that IgG bivalency may buffer the effects of reduced Fab affinity is plausible and consistent with established principles of antibody binding. However, the claim that this mechanism fully explains subtype-specific differences in resistance frequency requires additional supporting evidence, particularly from analysis of "live" strains, this could be made a bit clearer in the discussion. The proposed surveillance applications are promising but further demonstrating practical integration with existing RSV genomic surveillance efforts would increase confidence in the translational impact of the work. It would also be worth a comment in the discussion on the implications of this study in relation to the RSV vaccines that are now being used routinely (especially the maternal RSV vaccine). Overall, the conclusions are largely valid and provide a stepping stone for future work.
Suggested improvements: experiments, data for possible revision
Although there are additional experiments that could be done that would allow further analysis / interpretation of the concepts/ideas in this manuscript (e.g. some relating to the limitations noted above) and others e.g. clarification of clinical neutralisation thresholds, linking in vitro resistance to potential prophylaxis failure, I don't think there are any essential additional experiments required for this manuscript.

References: appropriate credit to previous work?
The manuscript appears to acknowledge previous studies describing monoclonal antibody resistance in RSV, including work on earlier antibodies such as palivizumab and suptavumab.
Clarity and context: lucidity of abstract/summary, appropriateness of abstract, introduction and conclusions
The abstract clearly outlines the key objectives and findings of the study and appropriately emphasises the relevance of antibody resistance to RSV prevention strategies. I have higher up commented on improvements that could be made to the discussion/conclusions. Overall, the manuscript is well written and logically structured.

Overall assessment:
This manuscript presents a potentially important analysis of RSV antibody resistance. The deep mutational scanning dataset represents a valuable resource, and the study addresses a timely question given the growing global use of monoclonal antibody prophylaxis. With additional validation and expanded discussion, the work could make a significant contribution to RSV research and surveillance.

(Remarks on code availability)

Version 1:

Reviewer comments:

Referee #4

(Remarks to the Author)

Thank you for addressing the points made by the reviewers I have no additional comments.

(Remarks on code availability)

Open Access This Peer Review File is licensed under a Creative Commons Attribution 4.0 International License, which permits use, sharing, adaptation, distribution and reproduction in any medium or format, as long as you give appropriate credit to the original author(s) and the source, provide a link to the Creative Commons license, and indicate if changes were made.
In cases where reviewers are anonymous, credit should be given to 'Anonymous Referee' and the source.
The images or other third party material in this Peer Review File are included in the article's Creative Commons license, unless indicated otherwise in a credit line to the material. If material is not included in the article's Creative Commons license and your intended use is not permitted by statutory regulation or exceeds the permitted use, you will need to obtain permission directly from the copyright holder.
To view a copy of this license, visit https://creativecommons.org/licenses/by/4.0/

Thanks for the careful and thoughtful reviews. We were glad that the reviewers were positive about our manuscript. We appreciate their helpful suggestions, which we have addressed in the revised manuscript. Below we provide a point-by-point response. The reviewer / editor comments are in **blue text** and our responses are in **black text**. In the revised manuscript itself, revisions and additions are indicated in **red text**.

Editor Comments

Your manuscript, "Complete definition of how mutations affect antibodies used to prevent RSV", has now been seen by 3 referees (and one co-reviewer), whose comments are attached below. While they find your work of potential interest, as do we, they have raised important concerns that in our view need to be addressed before we can consider publication in Nature.

Reviewer 1 feels the scope of the paper is somewhat narrow and misses an opportunity to provide insights into population-level immune pressures using human polyclonal sera. If this is possible, we would encourage you to add such insight. Reviewer 3 (whose expertise is more on the clinical side) feels the paper would be strengthened by experimental confirmation of key escape mutations in live RSV systems – we will overrule this request, considering the biosafety implications.

Should further experiments allow you to address the reviewers' criticisms, we would be happy to consider a revised manuscript (unless something similar has been accepted at Nature or appeared elsewhere in the meantime). We hope to receive your revised paper within four to six months. If you cannot complete the required revisions within this time frame, please let us know when you would anticipate being able to submit a revised manuscript.

Thanks for the summary of the reviews. In the revised version we have performed substantial new experiments to address all of the key reviewer points. Specifically, we have:

- Measured how mutations affect neutralization by polyclonal human sera, providing insights on how population immunity places pressure on RSV F, as well as the overlap from selection from serum antibodies and pressure from clinical monoclonal antibodies.
- Characterized how mutations affect two additional recently described antibodies (1A2 and 1B6) that are being developed as a potential clinical cocktail. This new work increases the relevance of our study for the development of new antibody prophylactics.
- Added new experiments showing how measuring mutation effects on Fab neutralization can help predict the impact of combinations of multiple mutations on IgG neutralization.
- Made additional clarifications as suggested by the reviewers.

Referee #1 (Remarks to the Author):

The authors built a pseudovirus-based DMS system for the RSV F protein and present a comprehensive DMS study to evaluate how mutations in the RSV F protein affect viral cell entry and neutralization by several key clinical mAbs, such as nirsevimab and clesrovimab. The study also proposes a biophysical model to explain subtype-specific differences in escape mutation prevalence based on the buffering effect of bivalent IgG binding. Furthermore, the authors integrate their DMS escape scores into the Nextstrain platform to facilitate real-time genomic surveillance of circulating RSV strains.

Overall, this study is technically sound, and the DMS results provide a critically detailed analysis that could explain or forecast viral escape from previous or existing therapeutic neutralizing antibodies. However, this study currently does not provide a substantially improved understanding of RSV evolution, nor does it give critical and direct insights into the development of vaccines or therapeutics against RSV, especially for neonates, at least in its current form. Below are my major concerns and suggestions:

As described in the point-by-point responses below we have added substantial new data that quantifies how mutations affect neutralization by human sera as well as analyzing more new antibodies being developed as potential RSV prophylactics. These new data expand the scope of our study and should address these concerns.

Major Points

1. The biophysical model presented in Figure 1, which describes the buffering effect of bivalent IgG binding versus monovalent Fab binding, is based on well-established principles and lacks substantial conceptual novelty. While the authors provide extensive experimental comparisons between Fab and IgG affinities throughout the manuscript, these data feel somewhat disconnected from the core biological implications of the study (RSV antibody escape). I suggest moving this model and the related text to the supplemental materials. Otherwise, the authors are expected to clearly justify and provide direct evidence in the main text as to why Fab escape data are biologically necessary for understanding real-world, antibody-mediated immune pressure and viral evolution.

We agree that IgG neutralization is the clinically relevant measurement. However, measuring mutation effects on Fab neutralization is evolutionarily relevant because mutations that reduce Fab neutralization also reduce IgG neutralization when they arise in a genetic background for which the antibody has lower affinity (eg, subtype B) or are combined by evolution. The first point was demonstrated in the original manuscript where we showed that for nirsevimab,

mutations that reduce nirsevimab Fab neutralization in subtype A reduce nirsevimab IgG neutralization in RSV B (Figure 3). To elaborate the second point, we have added new experimental data (shown below) demonstrating how naturally observed RSV-B mutations affect Fab and IgG neutralization of RSV-A alone and in combination. While these mutations individually reduce Fab but not IgG neutralization, in combination they reduce both Fab and IgG neutralization. Therefore, Fab measurements reveal mutations that combine to affect IgG neutralization. So from an evolutionary perspective, measurement of mutations on both Fab and IgG neutralization is relevant, as the real-world evolution of RSV involves accumulation of multiple mutations over time.

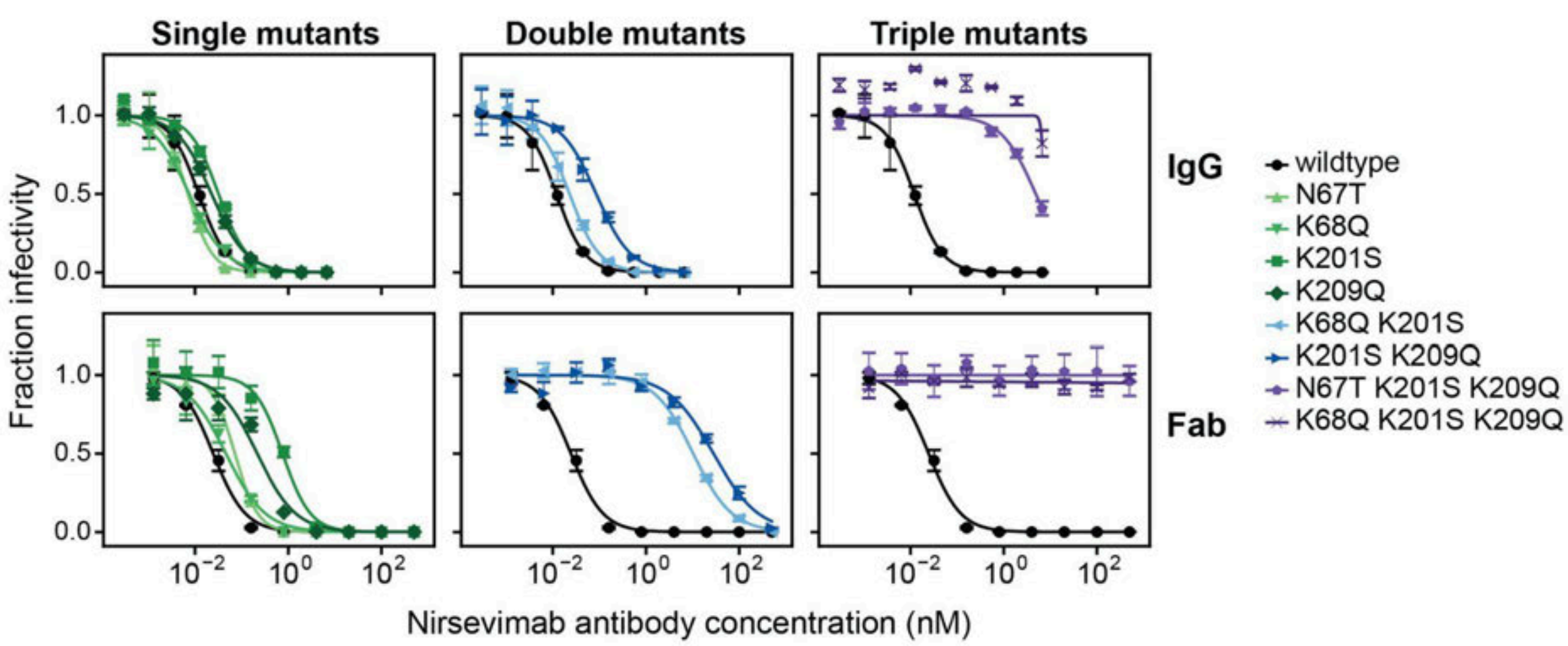

New data added as **Extended Data Figure 5** in the revised manuscript showing the relevance of characterizing Fab neutralization for understanding how combinations of mutations affect IgG neutralization. Individual mutations that reduce Fab but not IgG neutralization combine to reduce IgG neutralization, consistent with the biophysical model. Therefore, identifying Fab escape mutations can foreshadow changes that can combine to erode clinically relevant IgG neutralization.

2. The DMS platform developed by the authors is robust and technically impressive. However, the study evaluates only a very limited number of specific therapeutic mAbs or candidates (e.g., nirsevimab, clesrovimab, suptavumab, palivizumab, RSM01). Consequently, the manuscript reads more like a high-quality product-testing report for existing drugs rather than a systematic exploration of RSV F protein mutational rules and broader antigenic evolution. While this is highly useful for monitoring resistance to current clinical therapies, it does not yield the comprehensive antigenic mapping required to directly inform the design of new vaccines or the discovery of novel therapeutic antibodies.

To expand the scope of our study related to new candidate prophylactic antibodies, we have added deep mutational scanning data for 1A2 and 1B6, which are being considered as a possible

anti-RSV cocktail as described by Sun et al (2026, *Science Translational Medicine*, DOI: 10.1126/scitranslmed.ady2450). These new data are in Figure 6 and Extended Data Figure 9 of the revised manuscript. As shown in the reprinted Extended Data Figure 9 below, our new data enable direct comparison of these candidate new antibodies to existing clinical antibodies. Note that our paper already included data for RSM01, which is a lead candidate antibody that has been supported by the Gates Foundation. These new data and their implications for antibody development are discussed in the second-to-last Result section of the revised manuscript.

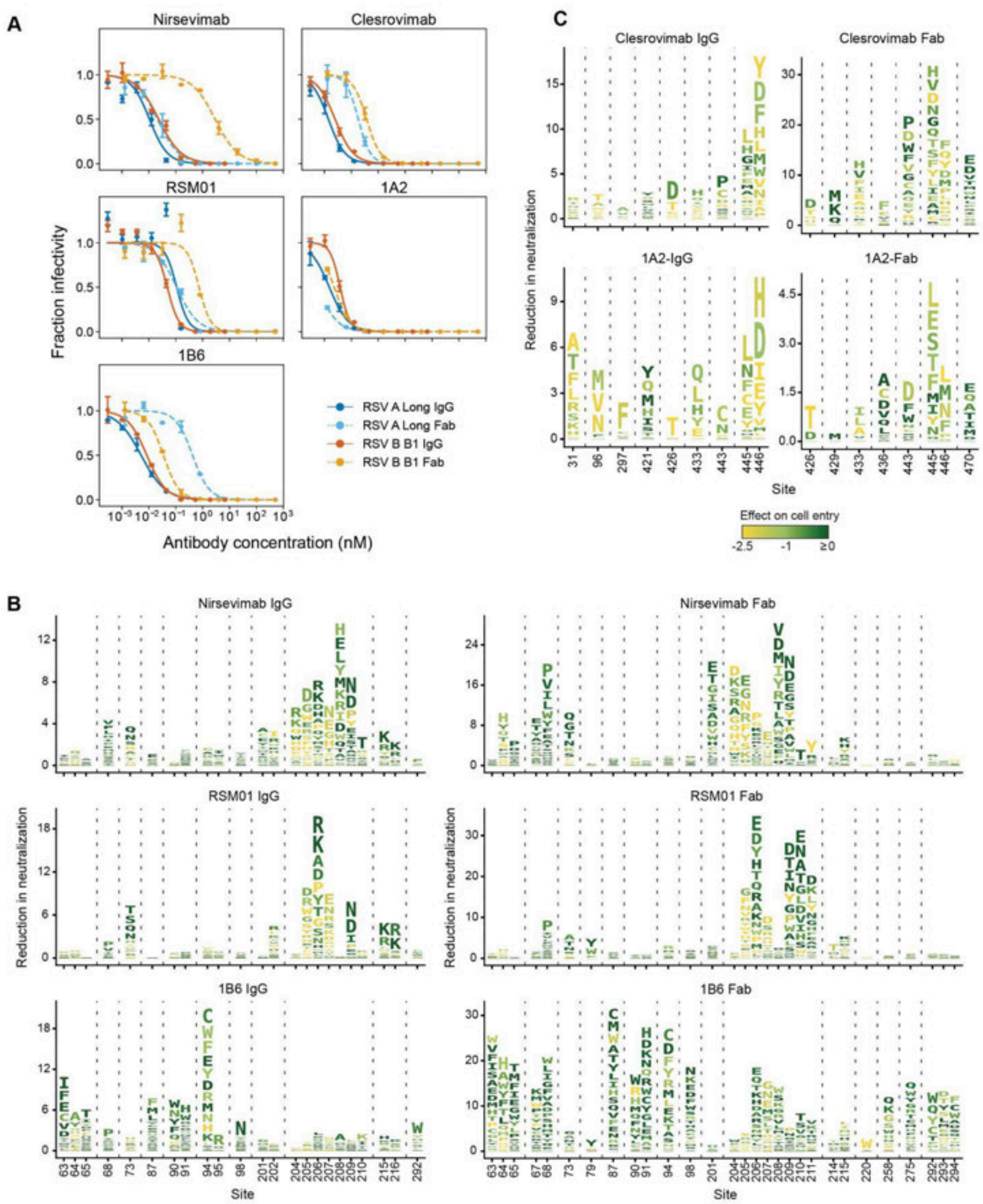

New data added as **Extended Data Figure 9** uses deep mutational scanning to characterize how mutations affect the candidate clinical antibodies 1A2 and 1B6, which were recently published as a potential antibody cocktail. These new data alongside the existing data for RSM01 (a lead candidate antibody supported by the Gates Foundation) prospectively assess the potential for resistance for these new candidate antibodies in comparison to the existing clinical antibodies nirsevimab and clesrovimab.

As a result, the core scientific problem addressed—mapping escape mutations for a few prophylactic mAbs—is somewhat narrow in scope for Nature. To substantially elevate the broad significance of this work, the authors need to establish a direct connection between RSV subtype evolution and broader, population-level immune selection pressures. I strongly recommend performing escape assays using human polyclonal sera (e.g., convalescent sera from natural infections or post-vaccination sera). Integrating polyclonal sera data would allow the authors to systematically link the evolutionary trajectories of RSV subtypes A and B to actual population-level immune pressures, thereby providing concrete and direct implications for future vaccine and antibody development.

We agree that understanding how population-level immune pressure might drive RSV F evolution is critical. For instance, for SARS-CoV-2, immune pressure from polyclonal antibodies elicited by prior infection and vaccination led to the rapid emergence of new viral variants with reduced neutralization. However, prior work shows that population-immune level pressure does **not** strongly drive the evolution of RSV F, as shown by both our group (Simonich et al., *J Virol*, 2025, https://doi.org/10.1128/jvi.00531-25) and a study by Pfizer (https://doi.org/10.1038/s41541-026-01418-8). For instance, the figure below excerpted from our prior study shows that historical sera collected in the 1980s neutralizes both old (1980s) and recent (2020s) RSV F variants similarly. This stands in sharp contrast to what is observed for viruses such as influenza and SARS-CoV-2. Therefore, measuring how mutations affect polyclonal serum neutralization may not be highly predictive of future RSV F evolution.

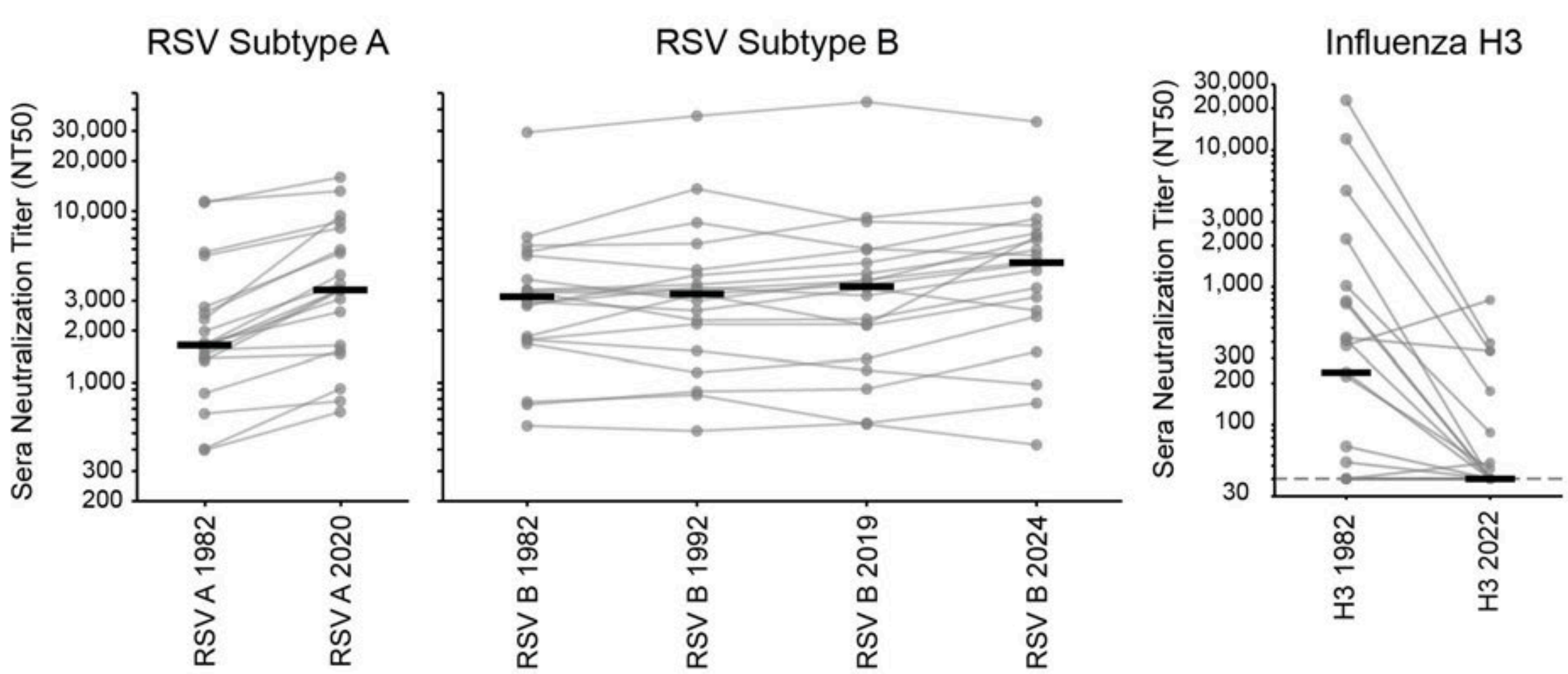

Figure 6 from our prior study (https://doi.org/10.1128/jvi.00531-25) showing similar neutralization by historical human sera (collected in 1980s) against historical and recent strains. In contrast, for influenza the historical sera titers are lower against a recent strain due to antigenic evolution of HA.

To further address this point, we have added substantial new experiments to the current manuscript that use deep mutational scanning to measure how mutations to RSV F affect neutralization by human polyclonal human sera. These new results are reported in a new final Results subsection of the revised manuscript and in Extended Data Figure 10, [redacted] These new experiments show that unlike for the monoclonal antibodies nirsevimab and clesrovimab, no mutations strongly reduce serum neutralization (see also Extended Data Figure 10B in the revised manuscript for further validation of this point).

[REDACTED]

[redacted]

As shown in the newly added data in Extended Data Figure 10 [redacted] polyclonal human antibodies target multiple antigenic regions of RSV F, including sites that overlap the epitopes of clinically relevant monoclonal antibodies as well as additional distinct regions. The mutations that did cause measurable decreases in serum neutralization tended to be at sites on the apex of F in a region more similar to the epitope of nirsevimab than clesrovimab, consistent with prior work showing that a substantial fraction of neutralizing anti-F antibodies encoded by human memory B cells target the apex (Gilman et al., *Sci Immuno,* 2016, DOI: 10.1126/sciimmunol.aaj1879) and that nirsevimab resistant strains have modestly reduced neutralization by a subset of human sera (Simonich et al., *J Virol,* 2025). Therefore, if RSV F does evolve under pressure from human sera in the future, that is more likely to lead to escape from nirsevimab than clesrovimab.

Note also that unlike for SARS-CoV-2 or influenza, a substantial fraction of the population (likely half of all infants or ~0.5% of all individuals in the United States) will be administered a monoclonal antibody to RSV in any given year. Therefore, monoclonal antibodies themselves could also start to put a meaningful selection pressure on the virus. We have extended the discussion of these topics in the last Result section and Discussion section of the paper.

3. Compared to proteins like the SARS-CoV-2 Spike, a notably larger number of sites on the RSV F protein severely impact cell entry. The authors should explain the underlying mechanism for this high functional constraint. For example, could the authors analyze their relationship to the structural features of the F protein? Additionally, in the validation assays, some individual mutations showed a negligible impact on cell entry, whereas they received notably low scores in the DMS dataset (Fig. 2B). The authors should address whether this discrepancy might lead to an overestimation of mutational impacts in the DMS results.

The reviewer is correct that RSV F appears to be more functionally constrained than the SARS-CoV-2 spike. We speculate that this is because most of F is involved in membrane fusion activity, which tends to be a more constrained protein function than receptor binding. In particular, RSV uses two separate proteins for cell entry, with G mediating attachment and F mediating membrane fusion, whereas SARS-CoV-2 spike combines receptor-binding and fusion functions within a single protein. The S2 subunit of spike, which contains the fusion machinery, is the closest functional analogue of RSV F and is more functionally constrained than the receptor-binding S1 subunit. The functional constraint on F is more similar to that on the S2 subunit of spike, but quantitative comparison across deep mutational scanning on entirely different proteins in different cell lines is challenging due to the lack of an internal standard.

With regard to the cell entry validation assay in Figure 2B, the results in that figure do show an excellent correlation with the deep mutational scanning. All tested mutations with substantially negative effects on the deep mutational scanning did have reduced titers in the validation assays. The decreased titers of the mutations with modestly negative effects in the deep mutational scanning (eg, N67I and S215V) may appear modest on the log10 scale, but the titers are reduced 2-fold or more. Therefore, there is no discrepancy between the DMS and the validation assays. The reason that the correlation in the full scatter plot is not precisely linear reflects differences in assay dynamic range: the dynamic range of the DMS is such that the lowest measurable values are around -2.5, whereas the validation assay can distinguish between even 100-fold versus 1000-fold decreased titers. Thus, while the DMS assay has reduced resolution to distinguish highly deleterious (eg, 100 fold decrease) versus extremely deleterious (eg, >1000-fold decrease) mutations, all mutations the DMS identifies as deleterious validate as such, with a near-linear correlation until the dynamic range saturates for highly deleterious mutations.

4. The observation regarding mutations T101P and S99N highlights a known discrepancy between in vitro pseudovirus fitness in cell culture and authentic viral fitness in the human airway. The authors should expand upon this in the discussion to address how this limitation might affect the broader interpretation of the DMS cell entry scores for other uncharacterized mutations. Additional validation experiments or analyses may also be necessary to confirm the reliability of the cell entry DMS scores in reflecting true viral fitness.

We agree with the reviewer that the deep mutational scanning measurements presented here quantify the effects of mutations on RSV F-mediated cell entry in a cell line, which is a property that will often correlate with real-world viral fitness but is not equivalent to real-world viral fitness. For this reason, we take care in the manuscript to always refer to mutation effects on F-mediated *cell entry* (in a cell line), and never refer to effects on real-world viral fitness. For instance, we note for the discrepancy for T101P and S99N: "This exception highlights the fact that there can be discrepancies between how mutations affect pseudovirus entry in cell culture versus real-world viral fitness: presumably the N99 and P101 identities in natural sequences are better for authentic viral infection of human airway cells even if S99 and T101 give better pseudovirus entry into a cell line in the lab." Also, the final paragraph of the Discussion discusses additional determinants of viral fitness that are not captured by our deep mutational scanning measurements.

One could imagine experiments that performed deep mutational scanning on authentic RSV in human primary airway cells to better measure mutation impacts on real fitness. However, such experiments would potentially run afoul of gain-of-function regulations. For this paper, the

editor specifically instructed us (see Editor's note at top of this document) ***not*** to test any mutations in replicative RSV for this reason. So overall, we agree with the reviewer about this caveat and have made sure to clearly note it. However, our data are still valuable because they provide information on aspects of F's functional constraint and more importantly on how mutations affect antibody neutralization, which is a well-established real-world correlate of protection in humans.

5. The study discusses that the same amino acid mutations can escape mAbs differently depending on whether they are in a subtype A or subtype B background. However, the DMS library was constructed solely based on subtype A. Why did the authors not include a subtype B-based variant library in the DMS? Including this would facilitate a much more systematic analysis of the distinct antibody escape mechanisms between the two subtypes.

We agree with the reviewer that a deep mutational scanning library constructed in a subtype B background would provide an even more comprehensive comparison of antibody escape across RSV subtypes, and is an important area for future work. However, our manuscript demonstrates that the current deep mutational scanning in a subtype A strain is sufficient to draw important insights about RSV evolution and the impact of mutations in both subtypes A and B. Repeating the entire workflow for RSV B would be an expensive new project outside the feasible scope of the current study.

Specifically, our results show that much of the subtype dependence of mutations on antibody neutralization can be explained by the biophysical model shown in Figure 1, whereby higher-affinity binding of nirsevimab Fab to subtype A results in more effective buffering of escape mutations by bivalent IgG. Consequently, mutations that similarly reduce Fab neutralization of both subtypes can have different effects on IgG neutralization. Consistent with this model, we found that DMS measurements using Fab were relatively generalizable across subtypes (Figure 3), allowing us to identify mutations that reduce IgG neutralization in subtype B despite having little effect on IgG neutralization in subtype A. We further show that we were able to use our deep mutational scanning to effectively identify natural strains of RSV B that are resistant to both nirsevimab and clesrovimab (Figure 5C and Extended Data Figure 7D), demonstrating the value of the current data for surveillance in both subtypes.

6. Although the analysis indicates that there is no immediate escape risk for nirsevimab and clesrovimab, could sequence analysis reveal other mutations under evolutionary pressure, and can these be mapped to specific antibody epitopes or functional DMS results? It would significantly strengthen the paper to explore whether historical RSV evolution demonstrates clear signatures of antibody-mediated selective pressure.

Prior studies, including our previous work (Simonich et al., J Virol 2025) and work by other groups (Kister and Bedford, Cell Host & Microbe 2023) have shown that RSV F evolves much more slowly than viral proteins such as influenza HA or SARS-CoV-2 Spike, with historical sequence evolution causing at most modest erosion of neutralization by human polyclonal sera as shown in the figure under our response to point (2) by Reviewer 1.

As discussed in our response to point (2) by Reviewer 1 above, to further address the relationship between monoclonal antibody escape and population immunity, we performed deep mutational scanning with human polyclonal sera. These experiments showed that mutations mildly reducing serum neutralization occur at multiple antigenic regions, including sites overlapping the regions targeted by clinically relevant monoclonal antibodies, particularly apex-directed antibodies such as nirsevimab. However, the effects of these mutations on serum neutralization are modest. Taken together, the sera DMS and limited historical evolution of RSV F indicate that antibody-mediated selection has not historically driven the strong antigenic evolution observed for viruses such as influenza or SARS-CoV-2. However, as we discuss in the Discussion section of our manuscript, for RSV the fact that use of clinical antibodies such as nirsevimab and clesrovimab is more widespread at a population level than the prior use of any other antiviral antibody (more than half of all infants receive these anti-RSV in some countries), it is quite possible that these antibodies themselves could start to help drive RSV evolution.

Minor Points

1. Please clearly define what the error bars represent in Fig. 2B, 3D, 3F, 4D, and 5C-D, as well as the black lines in Fig. 2E.

Text has been added to figure legends to define what these represent. For Fig. 2B each point represents the mean of two replicate measurements and error bars show ± 1 SD. For the neutralization curves shown in Fig. 3D, 3F, 4D and 5C-D, points indicate the mean ± standard error of technical replicates.

2. The numbers "2e6", "12e6", and "30e6" in the Methods section should be formatted using standard scientific notation.

The text has been revised and these numbers are now in standard scientific notation.

Referee #1 (Remarks on code availability):

No major problem, since it was based on their previous well-recognized SARS-CoV-2 platform.

We are glad that the authors were able to access the code, which uses a fully reproducible analysis pipeline.

Referee #2 (Remarks to the Author):

This study addresses an important research question of how a comprehensive library of single RSV F mutations affects prophylactic mAbs. The manuscript presents a hypothesis-driven model that shows that the bivalency of IgG may retain neutralization activity against viral mutations in spite of a reduction in Fab affinity but depending on whether the initial Fab affinity was high or moderate. The model is combined with deep mutational scanning of the RSV F Long strain.The authors systematically measure the effects of amino acid substitutions on F phenotypes including cell entry and neutralization escape from Fab and IgG versions of the prophylactic mAbs. The work provides an unprecedented dataset that can be directly integrated into RSV surveillance pipelines, enabling rapid interpretation of newly observed mutations. However, this needs to be clarified in a short section explicitly outlining how surveillance might use these datasets. The data is presented in a clear and accessible manner, with figures that are easy to follow and that are well designed. Overall, this work adds valuable information to the field.

We thank the reviewer for the nice summary of our study and the recognition of its value to the field.

While the deep scanning technique is not novel, application to the RSV field hasn't been done before, and this is a valuable tool with public health utility. While the conceptual framework in figure 1 is similarly not unprecedented, I find the combination of the two powerful in thinking about viral escape, not just from RSV. This combining the mutational work with the mechanism behind the viral escape strengthens the paper.

We agree that the combination of the biophysical model and deep mutational scanning increase the insight about viral evolution that can be drawn from the data presented in our study.

The title is vague eg no mention of F. I also wonder if there is a way to incorporate the findings re fab/IgG somehow into the title.

This is a good suggestion. We have changed the title to explicitly mention that we are studying mutations to RSV F (the new title is "Complete definition of how antibodies used to prevent RSV are affected by F mutations" compared to the old title of "Complete definition of how

mutations affect antibodies used to prevent RSV"). We agree that it would be nice to also mention the Fab and IgG results in the title, but were unable to find a sufficiently succinct way to do that given the word limits. So we have instead been sure to clearly mention the Fab and IgG results in the Abstract.

The abstract summarizes the methods but does not give a summary of the main results, please add to the abstract.

Thanks for the suggestion. We have revised the abstract to more clearly state the main results, including that nirsevimab resistance in subtype B is more common due to reduced Fab potency to this subtype, that resistance mutations are currently only sporadic and rare, and how potency and epitope-specificity shape escape.

The introduction is clear and concise
Recent and relevant references were used. However, in the introduction there was some over-citing, please carefully go through the references and select high quality focused sets. Do references 4 and 75 add any value? In addition there were some inconsistencies with the formatting of references 9, 32 and 88, please correct. If removing reference 4 please re-write the second half of 1st sentence.

Thanks for carefully checking the reference, these references have been corrected.

Defining the sites on pre-F that Nirsevimab and Clesrovimab recognise might improve readability.

We have added text to the Introduction noting that nirsevimab binds antigenic region Ø and clesrovimab binds antigenic region IV.

Please include evolutionary rates of F, HA and spike to add clarity

Due to the difficulty of precisely defining these rates (eg, the rates of evolution of H1 and H3 HA differ, and the rate of evolution of SARS-CoV-2 spike has modestly decreased over the last few years), we are not able to succinctly describe numerical rates in the Introduction given the length constraints. So we have instead included a qualitative description (HA and spike evolve faster than F), and added a citation to a detailed study by Kistler and Bedford (DOI: 10.1016/j.chom.2023.09.012) that quantitatively compares these rates.

Please mention the year and location of the Suptavumab trial.

We have added text to the introduction clarifying that the trial ran from 2015-2017. The study was performed across a wide range of locations (18 countries total) including multiple European countries, South Africa, the United States, Panama, and New Zealand. This location information is too complex for us to concisely summarize in the introduction, so we have included a link to the study reporting the clinical trial which contains all of this information.

The method is very detailed and the approach used has been previously described by Dadonaite et al, in the context of deep mutational scanning for SARS-CoV-2. Therefore this could be written more concisely, focusing on modifications to protocol. A few sections require minor revisions such defining abbreviations upon first appearance and using terms in a consistent manner for example dox versus doxycycline.

We thank the reviewer for this suggestion, and have attempted to make the methods as concise as possible while still retaining all critical details for readers who might want to reproduce the work. While the methods are still fairly lengthy, they will appear online in any final published version so when in doubt we erred on the side of completeness to enable reproducibility over succinctness. We have removed the dox abbreviation and made sure all other abbreviations are adequately defined.

The quality of the images is high including interactive figures. Some minor edits are suggested Figure 1 in panel C- please clearly state Subtype A with high affinity and Subtype B with low affinity and in the legend provide clarity that A is the illustration of the biophysical model in which the hypothesis is…as the second part of the statement seems open-ended. B orient the reader by defining the colours and shapes.

We have added the labels about affinity to subtypes A and B in Figure 1C, and have modified the figure legend to make clear that panel A is illustrating the biophysical model.

In general, the figure legends appear as interpretation of results instead of giving the reader direction on the figure.

We have revised the figure legends where appropriate to focus more on describing the contents of each panel and directing the reader through the figure. However, because several figures contain multiple panels and complex data visualizations, some of our legends do include some additional interpretation intended to guide the reader.

Supplementary figure 1- define MOI.

We have defined MOI as "multiplicity of infection" and clarified that we used an MOI of 0.01.

Supplementary figure 2- Specify correlation test used and add p-value (this should be done for all correlations reported).

We have specified that the reported values are Pearson correlation coefficients. Note that the replicate correlations quantify experimental reproducibility as a quality-control. Specifically, they represent the correlation in two independent measurements of the same biological property, and therefore they are expected to be correlated. These particular correlations do not test a hypothesis, and the p-values are all $\ll 10^{-10}$ due the very large number of measurements, so the p-value is not relevant for Supplementary Figure 2. We have been sure to add p-values to all the other figure legends where they are meaningful.

For consistency and ease of reading can Figure 3 D and F be merged as done for Figure 4 D. It is not clear why Figure 3 E doesn't show the effects of mutations across Subtype B?

We appreciate these suggestions, but prefer to keep the current layout. For the neutralization curves for nirsevimab (Figure 3D and 3F), there are many more tested mutations than for the corresponding curves for clesrovimab (Figure 4D). For that reason, the plots did not seem visually accessible when that many curves were stacked together in a single panel so we think the current layout of two panels is easier to visualize after examining both layouts. For Figure 3E, the corresponding data for subtype B is in Extended Data Figure 4. Due to space constraints we were unable to fit all of these panels in Figure 3 since we have more data to plot for nirsevimab and than clesrovimab. The reason we have more data to show and need additional plots for nirsevimab is to make clear that this antibody behaves somewhat differently versus subtype A and B, unlike clesrovimab.

Supplementary Figure 7, Panel A, please re-arrange the mAb Fab and IgG plots so the order is consistent.

This is a good suggestion. The extended data figures have been rearranged so that the order is consistent now.

While our expertise is not in reviewing the code or formulas for analysis, the supplementary appendix describes in detail the modelling of how the biophysical properties of the antibodies affect neutralization activity to viral antigen mutations. Included here is an interactive plot in which one can adjust affinity and determine the impact on neutralization capacity of a Fab

region versus IgG, this strengthens the models application as different conditions can be simulated as a proof of concept. We would defer to a reviewer more able to evaluate this section

We're glad the reviewer found the supplementary appendix and interactive plot useful; our hope was indeed that this plot would make it easy to examine the predictions of the biophysical model.

Referee #3/4 (Remarks to the Author):

Summary of the key results
This manuscript investigates how mutations in the RSV F protein influence neutralization by monoclonal antibodies currently used or under development for RSV prevention. The study is motivated by the increasing use of long-acting monoclonal antibodies (mAbs) which have substantially reduced RSV-related infant hospitalisations. The authors propose a biophysical explanation for observed differences in resistance mutation frequency between RSV-A and RSV-B strains, arguing that the buffering effect of bivalent IgG binding can partially compensate for reduced affinity caused by viral mutations. They have performed pseudovirus deep mutational scanning to evaluate the effects of nearly all possible amino-acid substitutions in the RSV F protein on viral entry and antibody neutralisation. This approach is used to quantify the neutralisation landscape for several anti-RSV mAbs, including nirsevimab and clesrovimab. Finally, the authors demonstrate how experimentally derived escape maps can be applied to surveillance datasets to identify circulating RSV strains carrying mutations associated with reduced antibody susceptibility. The work aims to support early detection of antibody resistance and guide development of future monoclonal antibodies with improved resilience to viral escape.

Originality and significance
The question addressed by the study is highly significant. As mAbs become a cornerstone of RSV prevention, particularly for infants, the emergence of viral escape mutations represents a major public health concern. The use of deep mutational scanning to generate a comprehensive functional map of RSV F protein mutations affecting antibody neutralisation represents an important technical and conceptual advance. While deep mutational scanning has previously been applied to other viral surface proteins, its application to RSV antibody escape remains relatively limited. Previous work has identified resistance mutations affecting monoclonal antibodies targeting RSV F, including escape mutations associated with palivizumab and suptavumab. However, these studies have generally relied on selection experiments or targeted mutagenesis rather than systematic mutational mapping. In that context, the comprehensive mutational landscape presented here appears novel and potentially valuable for surveillance

and antibody design. The proposed biophysical model linking IgG bivalency and Fab affinity to subtype-specific resistance frequencies is conceptually interesting, although its broader significance will ultimately need stronger corroborative evidence. Overall, the manuscript addresses an important problem and provides data that could have substantial implications for RSV surveillance and therapeutic antibody development.

We thank the reviewer for the nice summary and appreciation of how this work can advance understanding of RSV antibodies, surveillance, and future antibody development.

Data & methodology: validity of approach, quality of data, quality of presentation
The experimental approach combines biophysical modelling, pseudovirus assays, and deep mutational scanning, which together provide a powerful framework for mapping antibody escape mutations. The deep mutational scanning methodology appears appropriate, having been adapted from previous work. Generating a near-complete mutational dataset for the RSV F protein is notable and provides a detailed view of the functional constraints governing antibody escape.

However, several methodological limitations should be noted, alongside those the authors already highlight:

1. Although pseudovirus assays are widely used for neutralisation studies, they may not fully capture the replication dynamics and structural constraints present in authentic RSV infection. Certain mutations identified as escape variants in pseudovirus systems may not be viable in replication-competent viruses.

We agree that pseudovirus assays may not fully recapitulate all aspects of authentic RSV, and have included a clear statement of this caveat in the manuscript. However, at least for the RSV-A A2 strain, our pseudovirus neutralization measurements correlate well with measurements made using authentic RSV. Specifically, in previous work describing the pseudovirus system, we found strong agreement between measurements of neutralization by a panel of human sera against pseudovirus and authentic RSV (see Figure 3A below from Simonich et al, *J Virol*, 2025, (DOI: https://doi.org/10.1128/jvi.00531-25). We agree it is likely that the pseudovirus system is more tolerant of F mutations than authentic virus, and so some mutations identified in the pseudovirus deep mutational scanning may not be tolerated in authentic virus. However, we view the main value of our research as enabling immediate assessment of the impact of mutations observed in real-world RSV surveillance, and any mutations observed in such surveillance must have at least some capability of supporting replication of authentic virus.

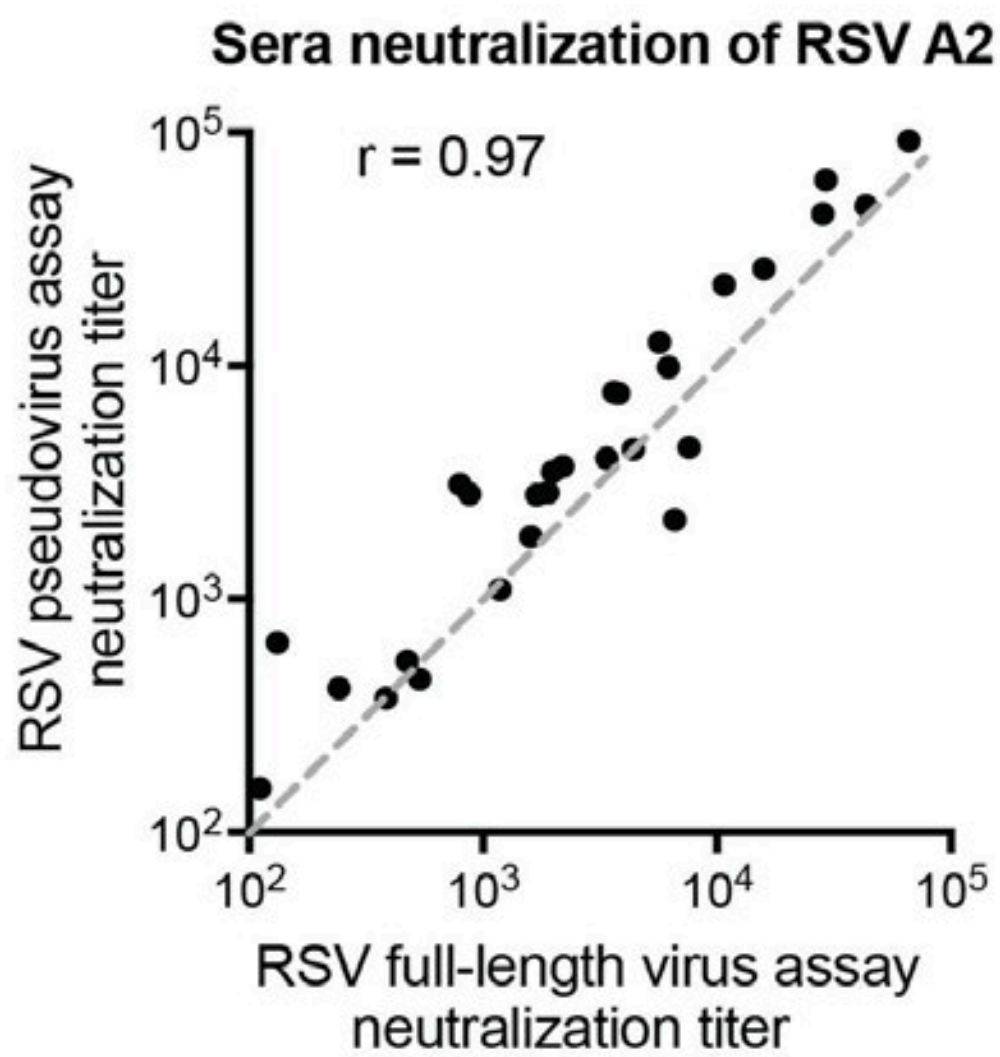

Figure 3 from our prior study (https://doi.org/10.1128/jvi.00531-25) showing excellent correlation for sera neutralization measured using RSV pseudovirus versus authentic virus.

2. The manuscript would be strengthened by experimental confirmation of key escape mutations in live RSV systems.

For this paper, the editor specifically instructed us (see Editor's note at top of this document) ***not*** to test any mutations in replicative RSV due to recent new gain of function regulations. However, we included comparisons of our deep mutational scanning to prior published measurements of mutation effects measured in authentic RSV. Specifically, mutations in authentic RSV that reduce susceptibility to nirsevimab (N208D, K68N, N201S and N201T), clesrovimab (S443P, G446E) and RSM01 (I206T) also reduced neutralization susceptibility to the corresponding antibody in our deep mutational scanning results. These findings corroborate our deep mutational scanning on at least a subset of mutations; see the following list of citations for the papers with the live-virus neutralization data for these antibodies: Chen et al., *npj Vaccines*, 2026 https://doi.org/10.1038/s41541-026-01418-8; Ahani et al., *Nature Communications,* 2023, https://doi.org/10.1038/s41467-023-40057-8; Terstappen et al., *bioRxiv,* 2026, https://doi.org/10.64898/2026.02.04.703721; U.S. Food and Drug Administration. *ENFLONSIA (Clesrovimab-Cfor) Prescribing Information*. https://www.accessdata.fda.gov/drugsatfda_docs/label/2025/761432s000lbl.pdf (2025).

3. While the authors demonstrate how their mutational maps could be used to screen sequence databases, the practical implementation of this approach in real-time surveillance systems remains underdeveloped at this stage.

The surveillance framework described in this study has already been implemented as a publicly available Nextstrain resource that automatically integrates newly deposited RSV sequences with the experimentally measured phenotypic effects reported here. The platform can be viewed at https://nextstrain.org/rsv/b/F-antibody-escape/6y. We regularly view this platform to assess if there have been new observations of strains with resistance mutations.

Additionally, since posting the manuscript we have been contacted by other groups who have also asked us to perform analyses similar to the aforementioned publicly available Nextstrain view on sets of sequences they have collected but not yet made publicly available. Because we do not control decisions about making those sequences public, we cannot report the results here, but do anticipate studies led by other groups that use our data for RSV surveillance will be published in the future.

In addition, when we posted the preprint we created an accompanying website at https://dms-vep.org/RSV_Long_F_DMS/ with interactive plots of the DMS data. That website has received more than 600 page views from users in 12 countries, with many users engaging with the interactive views of the data on the website for extended periods of time. Although we do not know exactly how the website visitors are using the data (since we only track page views but do not ask users to login or provide additional details), these statistics provide evidence that our data are already being used for interpretation of mutations observed in RSV F.

So overall, the approach of our manuscript is to share our experimental data and corresponding visualizations publicly so they can be used by any other group to help analyze public or currently private sequence data, and there is strong evidence that our data are indeed being used in that fashion.

4. The RSV strain 'backbone' used for development of the pseudovirus was the Long strain from the 1950s, why not use a more contemporaneous strain?

We selected the Long strain because it is a well-characterized reference strain that has been widely used for RSV neutralization studies and shares approximately 98% amino acid sequence identity with contemporary subtype A RSV F sequences. In addition, the Long strain consistently produces high pseudovirus titers, which is helpful for deep mutational scanning. However, we validated the deep mutational scanning measurements in contemporary natural sequences, such as in Figure 5 where we used the deep mutational scanning to identify naturally occurring RSV F sequences with reduced susceptibility to nirsevimab or clesrovimab.

5. It is already highlighted in the limitations section, but data on combinations of mutations would be extremely helpful for understanding this area.

We have added new experiments that directly address this point by showing how individual mutations that reduce neutralization by Fab but not IgG combine to reduce IgG neutralization. Specifically, we examined combinations of naturally observed RSV-B mutations that individually have little effect on nirsevimab IgG neutralization in the RSV-A background but measurably reduce Fab neutralization. As shown in the newly added Extended Data Figure 5 of the revised manuscript (pasted below), these mutations have minimal effects on IgG individually but combine to reduce nirsevimab IgG neutralization. These findings demonstrate that Fab measurements can identify mutations that may combine to affect IgG neutralization, supporting the utility of the Fab datasets for anticipating the effects of multi-mutant variants. We further agree that additional experimental characterization of combinations of mutations would be a valuable future extension of this work, and have discussed this limitation in the Discussion as mentioned by the reviewer.

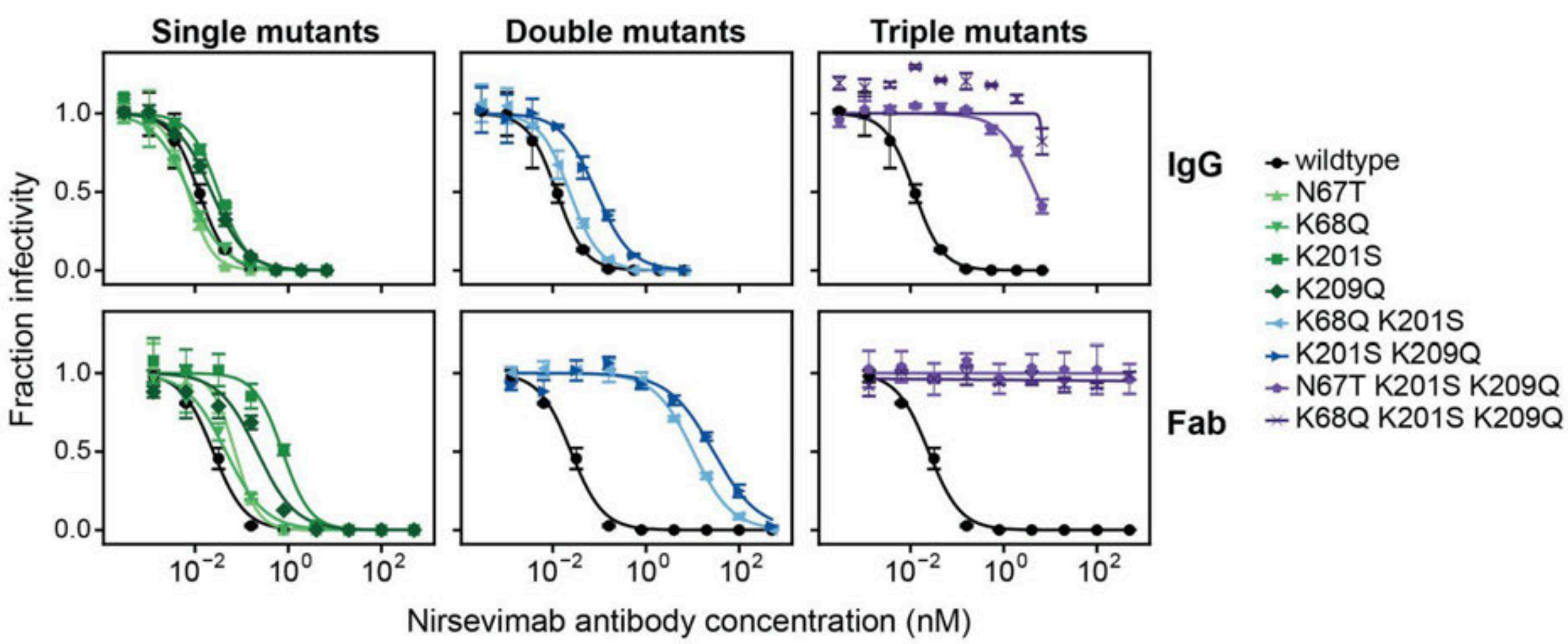

New data added as **Extended Data Figure 5** in the revised manuscript shows how individual mutations that reduce Fab but not IgG neutralization combine to reduce IgG neutralization, consistent with the biophysical model in the manuscript.

6. As part of the cloning of the deep mutational scanning plasmid libraries, 268 mutations were 'missed' in the library produced by Twist. What is the explanation for this?

The initial library was commercially synthesized by Twist, and although their synthesis approach attempts to include all mutations in the library, typically some mutations will be missing. The Twist synthesis method is proprietary so we do not know the details of what factors affect library quality, but we can use deep sequencing ourselves to independently

identify any missing mutations. To recover the mutations missing in the Twist-synthesized library, we generated a supplemental "spike-in" library by PCR mutagenesis in our lab that specifically incorporated the missing mutations. The spike-in library was combined with the original library prior to downstream experiments, which improved coverage of the missing mutations.

7. Why were the specific mutations that were chosen for the biophysical model (K68Q and N201S), rather than others? How were thresholds for defining “resistance mutations” determined?

K68Q and N201S were selected for the biophysical model validation because they are well-characterized nirsevimab resistance mutations. Our intent was to use representative, well-established resistance mutations to illustrate the biophysical mechanism by which differences in antibody affinity between RSV subtypes affect Fab and IgG neutralization. However, our paper also subsequently includes additional data for many other mutations that support the biophysical model. Figure 3 supports the model with additional mutations identified by deep mutational scanning that reduce nirsevimab Fab neutralization of subtype A and nirsevimab IgG neutralization in subtype B. The newly added Extended Data Figure 5 (reprinted in this response under point 5 by Reviewer 3) further supports the model by showing mutations that individually only affect Fab neutralization of subtype A can combine to reduce IgG neutralization.

Regarding the definition of resistance mutations, we intentionally did not apply a single threshold but rather used the fact that our experiments quantitatively measure the effect of each mutation. For instance, K68N modestly reduces neutralization, whereas Q210T causes a large drop in neutralization. These differences are quantified both in our deep mutational scanning (K68N has a modest positive escape value, whereas Q210T has a very large escape value) and in the validation neutralization assays, where K68N moderately shifts the neutralization curves whereas Q210T largely ablates measurable neutralization. We believe this approach of quantifying the effect of each mutation rather than creating a binary classification of “resistance” versus “not resistance” is more informative because even modest reductions in neutralization can be biologically relevant depending on antibody concentration, viral background, or the accumulation of additional mutations.

8. For clarity does “mutationally tolerant” mean it is less likely mutations will occur at the site or that if they do occur it is less likely to impact IgG/Fab binding?

We use the term "mutationally tolerant" to refer to mutations that are compatible with RSV F function, as measured by their ability to support pseudovirus entry, rather than their effects on

antibody binding or neutralization. In other words, a mutationally tolerated substitution is one that does not substantially impair F-mediated cell entry. Overall, we expect mutations that do not disrupt F's cell entry function to at least have a *possibility* of occurring during evolution (eg, they are tolerated), but the fact that a mutation is tolerated does not mean it will necessarily appear or have any impact on neutralization. We have revised the text to define this terminology more explicitly at its first use to avoid confusion.

9. The epitope for clesrovimab (same for palivizumab) is also on the postF form (compared with nirsevimab's which is only on the preF form), does that impact these results or the interpretation of the results?

The reviewer is correct that the clesrovimab and palivizumab epitopes are present on both preF and postF, whereas the nirsevimab epitope is specific to preF. However, this should not matter for our experiments or the interpretation of the results, since our experiments directly measure antibody neutralization of F (which is thought to typically require preF binding). This is an advantage of directly measuring neutralization compared to the binding-based measurements that have been used in some other studies; for binding based measurements, it is important to distinguish between preF and postF binding postF binding may not always translate to actual neutralization.

10. It is unclear to me how the data described evidence the statement "…since the biophysical model described above suggests that Fab measurements are more generalizable across genetic backgrounds due to the absence of affinity-dependent buffering of bivalent IgG neutralization."

We have revised the text to better explain the evidence supporting this conclusion. The statement is not based solely on the biophysical model. Rather, it is supported by multiple complementary observations. First, representative mutations that similarly reduce Fab neutralization in both subtypes can have markedly different effects on IgG neutralization because subtype-dependent differences in antibody affinity alter the extent of bivalent avidity buffering. Second, for nirsevimab, mutations identified by RSV-A Fab deep mutational scanning accurately predict mutations that confer escape from nirsevimab IgG in RSV-B (Figure 3), demonstrating that Fab measurements are more generalizable across genetic backgrounds than IgG measurements. Third, we have added new experiments examining combinations of naturally observed RSV-B mutations that individually have minimal effects on RSV-A IgG neutralization but measurably reduce Fab neutralization. While these mutations have little effect on IgG individually, combinations of two or three mutations reduce IgG neutralization while remaining compatible with viral entry (see the new Extended Data Figure 5).

Taken together, these results demonstrate that Fab escape measurements can reveal mutations with escape potential that becomes apparent only as mutations accumulate, supporting the relevance of the Fab measurements for understanding antibody escape during ongoing RSV evolution.

11. “Note that S211R slightly increases subtype B neutralization despite decreasing subtype A neutralization for both Fab and IgG; for this mutation there must be additional background-specific differences between subtypes”. This is important for implications of overall generalisability of the results and should be highlighted clearly in the limitations section.

The reviewer is correct that S211R demonstrates that there can be mechanisms of subtype-dependent mutation effects beyond those due to the bivalent IgG buffering captured by the biophysical model. We have specifically called out S211R to alert the reader to this caveat, and have included additional text highlight the limitation that there is always uncertainty in extrapolating measurements in one strain to another strain, even if (as we demonstrate in the paper) the deep mutational scanning has substantial utility for identifying variants with reduced neutralization across multiple strains of both subtypes.

I note a couple of minor typos: page 35 “palizumab” and page 36 “with” is written twice. Overall, the methodology appears appropriate and uses standard analytical approaches where feasible.

Thank you. These have been corrected.

Conclusions: robustness, validity, reliability

The central conclusion, that systematic mapping of RSV F mutations can reveal escape pathways and support surveillance for antibody resistance, appears well supported by the experimental data. The finding that IgG bivalency may buffer the effects of reduced Fab affinity is plausible and consistent with established principles of antibody binding. However, the claim that this mechanism fully explains subtype-specific differences in resistance frequency requires additional supporting evidence, particularly from analysis of “live” strains, this could be made a bit clearer in the discussion. The proposed surveillance applications are promising but further demonstrating practical integration with existing RSV genomic surveillance efforts would increase confidence in the translational impact of the work. It would also be worth a comment in the discussion on the implications of this study in relation to the RSV vaccines that are now being used routinely (especially the maternal RSV vaccine). Overall, the conclusions are largely valid and provide a stepping stone for future work.

We thank the reviewer for this thoughtful assessment and for recognizing the strengths of the study. As far as the caveats mentioned by the reviewer:

- We agree that the biophysical model provides a mechanistic explanation for much of the observed subtype dependence, but that there can be additional subtype-specific differences due to additional factors, including genetic background, epistasis, and other biophysical properties of RSV F. As discussed in response to specific comments (10) and (11) by the reviewer above, we have added additional data and discussion relative to this point.
- As discussed above, we have not added any live virus experiments as the editor specifically included a note about how this should not be done given current gain of function concerns. However, as discussed in response to specific comment (2) by the reviewer above, we have included detailed comparisons to prior published measurements on mutations made using live virus.
- As discussed in the response to specific comment (3) by the reviewer above, the data are now being used in some RSV surveillance mechanisms, and are publicly available to enable others to include them for additional uses.
- As suggested by the reviewer and described above (primarily in the response to Reviewer 1), we have performed new experiments measuring how mutations affect neutralization by polyclonal serum antibodies. This new work is in the last Result section of the revised manuscript, and elaborates on other recent findings indicating that RSV F evolution is unlikely to dramatically affect vaccine-elicited polyclonal antibody neutralization in the same way it does for more rapidly evolving viruses such as influenza and SARS-CoV-2.

Suggested improvements: experiments, data for possible revision
Although there are additional experiments that could be done that would allow further analysis / interpretation of the concepts/ideas in this manuscript (e.g. some relating to the limitations noted above) and others e.g. clarification of clinical neutralisation thresholds, linking in vitro resistance to potential prophylaxis failure, I don’t think there are any essential additional experiments required for this manuscript.

Thanks for assessing that additional experiments are not required. Due to requests by other reviewers, we have added additional data relating the effects of multiple mutations on Fab versus IgG neutralization, and on the impacts of mutations on neutralization by polyclonal serum antibodies. Even though the reviewer assessed these experiments as not essential, we think their inclusion has further strengthened the study.

References: appropriate credit to previous work?
The manuscript appears to acknowledge previous studies describing monoclonal antibody resistance in RSV, including work on earlier antibodies such as palivizumab and suptavumab.
Clarity and context: lucidity of abstract/summary, appropriateness of abstract, introduction and conclusions
The abstract clearly outlines the key objectives and findings of the study and appropriately emphasises the relevance of antibody resistance to RSV prevention strategies. I have higher up commented on improvements that could be made to the discussion/conclusions. Overall, the manuscript is well written and logically structured.

We are glad that the reviewer found the references to adequately contextualize prior work, and have made the suggested improvements to the discussion.

Overall assessment:
This manuscript presents a potentially important analysis of RSV antibody resistance. The deep mutational scanning dataset represents a valuable resource, and the study addresses a timely question given the growing global use of monoclonal antibody prophylaxis. With additional validation and expanded discussion, the work could make a significant contribution to RSV research and surveillance.

We thank the reviewer for the positive assessment, and think (s)he will find that the additional data and revisions we have made further strengthen the study.

Thanks for the careful and thoughtful reviews. We were glad that the reviewers were positive about our manuscript. We appreciate their helpful suggestions, which we have addressed in the revised manuscript. Below we provide a point-by-point response. The reviewer / editor comments are in **blue text** and our responses are in **black text**.

Your manuscript, "Complete definition of how antibodies used to prevent RSV are affected by F mutations", has now been seen by our referees, and in the light of their advice I am delighted to say that we can in principle offer to publish a further revised version of the paper, which addresses the concerns I have detailed below. We would also like you to make some editorial changes to your paper so that it is as brief as possible and complies with our Guide to Authors. No peer reviewed data should be removed altogether when making these changes.

As reviewer 2 did not have experimental requests last time we did not send the revision back to referee 2. Reviewer 3 is now satisfied with the revisions. Unfortunately reviewer 1 has raised concerns about the newly added data using the polyclonal sera. Although you added these data at the request of this reviewer, he/she does not feel these data support the conclusion that RSV-F evolution has only a limited effect on population-level serum neutralisation. The comments of this referee can be found below. Given the technical concerns raised about the resolution of the assay, it would seem that even the more limited conclusion suggested by the referee may not be supported.

Given the support from reviewers 2 and 3, however, have decided that we would be able to publish a paper that focuses only on the clinical antibodies (and therefore we would ask you to please remove the data with human polyclonal sera, which you added during revision), but that also makes it explicit that, for the most part, you are only looking at single mutations and that this may not reflect real world RSV-F evolution.

Thank you. We acknowledge the limitations of the human polyclonal sera DMS but agree that the manuscript focusing on the clinical and pre-clinical antibodies provides important insights about the mutational constraint of F and informs ongoing sequence surveillance at a critical time as these antibodies are more widely used. We have removed the data with the human polyclonal sera as requested.

Referee 1:

1. Based on historical sera and a pseudovirus DMS system, the authors conclude that RSV F evolution has only limited effects on neutralization by human polyclonal sera. In our view, their evidence supports a narrower conclusion: within a Long F pseudovirus single-mutant library, individual F mutations did not cause strong escape from the small number of human sera tested. This does not necessarily establish that real-world RSV F evolution has only a limited effect on population-level polyclonal serum neutralization.

We agree that our deep mutational scanning to measure effects of F mutations on neutralization by polyclonal sera has limitations as we included a small number of human sera and measured effects of mutations in the Long strain F. However, we also note that our prior study with

historical sera (1980s) included pseudoviruses expressing F from historical (1980s) and recent (2020s) clinical F sequences and therefore evaluated the effect of RSV evolution over decades on polyclonal sera neutralization (Simonich et al., *J Virol*, 2025, https://doi.org/10.1128/jvi.00531-25). However, given the limitations noted by the reviewer, and as recommended by the editor, we have removed the deep mutational scanning data for the human sera.

2. The authors' serum DMS conclusion relies on measuring the effects of single mutations against a limited number of human sera. For polyclonal sera, the effect of any individual mutation is inherently modest. If the pseudovirus serum DMS assay has a limited signal-to-noise ratio, or substantial variation in infection counts and functional scores across library variants, it may lack the power to detect moderate but biologically meaningful reductions in serum neutralization. Additionally, the failure to observe strong single-mutant serum escape does not exclude the possibility that circulating strains with multiple substitutions exhibit reduced neutralization sensitivity.

We agree that failure to observe strong single-mutant serum escape does not exclude the possibility that circulating strains with multiple substitutions exhibit reduced neutralization sensitivity. Given the limitations as noted by this reviewer and at the recommendation of the editor, we have removed the sera DMS data from the manuscript.

3. I believe the authors should further substantiate the reliability and biological relevance of the serum DMS component. In particular, it would be important to clarify whether the assay has sufficient resolution to detect moderate serum escape; whether serum dilution and virus input affect escape scores; whether the filtering of low-entry variants might underestimate potential escape sites; whether single-mutant escape scores can predict the combined effects of multiple substitutions in contemporary circulating strains; and whether the pseudovirus DMS results are consistent with authentic-virus neutralization data, especially for contemporary RSV-B strains.

These are good suggestions for future DMS studies of human serum.

Overall, the manuscript still presents an impressive pseudovirus DMS platform for defining intrinsic F-mediated cell-entry constraints, which is important. However, I am less convinced that the current antibody and serum DMS data are sufficiently representative to support broad conclusions about real-world RSV antigenic evolution or future epidemic trends, which undermines the effort of mapping all F mutations that escape current approved RSV antibodies.

We feel that the manuscript, particularly the DMS data for the monoclonal antibodies, enables phenotype-informed sequence surveillance as RSV evolves and acknowledge that, at this time, we are unable to robustly support broad conclusions about future RSV antigenic evolution or future epidemic trends. The best that can currently be done is to monitor for the real-world spread of resistance while continuing to develop new antibodies with distinct resistance profiles—both goals that our deep mutational scanning data help advance.

Referees' comments:
Referee #4 (Remarks to the Author):
Thank you for addressing the points made by the reviewers I have no additional comments.

We thank the reviewer for their helpful comments on the manuscript and are glad that we addressed the points raised in the initial round of review.
